# Phytochemical on-line screening and *in silico* study of *Helianthemum confertum*: antioxidant activity, DFT, MD simulation, ADME/T analysis, and xanthine oxidase binding†

**DOI:** 10.1039/d4ra02540g

**Published:** 2024-07-15

**Authors:** Yasmine Chemam, Samir Benayache, Abdeslem Bouzina, Eric Marchioni, Omar Sekiou, Houria Bentoumi, Minjie Zhao, Zihad Bouslama, Nour-Eddine Aouf, Fadila Benayache

**Affiliations:** a Unité de Recherche Valorisation des Ressources Naturelles, Molécules Bioactives et Analyses Physicochimiques et Biologiques, Université Frères Mentouri Constantine 1, Route d’Aïn El Bey 25000 Constantine Algeria yasmine.chemam@yahoo.fr; b Chimie Analytique des Molécules Bioactives, Institut Pluridisciplinaire Hubert Curien (UMR 7178 CNRS/UDS) 74 route du Rhin 67400 Illkirch France; c Laboratory of Applied Organic Chemistry, Bioorganic Chemistry Group, Department of Chemistry, Sciences Faculty, Badji Mokhtar Annaba University Box 12 23000 Annaba Algeria; d Environmental Research Center Alzon Castle, Boughazi Said Street, PB 2024 Annaba 23000 Algeria

## Abstract

Seven components from the methanol extract of the aerial part of the endemic species *Helianthemum confertum* were isolated and identified for the first time. Investigating this species and its separated components chemical make-up and radical scavenging capacity, was the main goal. Using an online HPLC-ABTS˙^+^ test, ORAC, and TEAC assays, the free radical scavenging capacity of the ethyl acetate extract was assessed. The fractionation of these extracts by CC, TLC, and reverse-phase HPLC was guided by the collected data, which was corroborated by TEAC and ORAC assays. Molecular docking studies, DFT at the B3LYP level, and an examination of the ADME/T predictions of all compounds helped to further clarify the phytochemicals' antioxidant potential. Isolation and identification of all components were confirmed through spectroscopy, which revealed a mixture (50–50%) of *para*-hydroxybenzoic acid 1 and methyl gallate 2, protocatechuic acid 3, astragalin 4, *trans*-tiliroside 5, *cis*-tiliroside 6, contaminated by *trans*-tiliroside and 3-oxo-α-ionol-β-d-glucopyranoside 7, as well as two new compounds for the genus *Helianthemum* (2 and 7). With a focus on compounds 1, 2, 3, and 4, the results clearly showed that the extract and the compounds tested from this species had a high antioxidant capacity. Within the xanthine oxidase enzyme's pocket, all of the components tested showed strong and stable binding. In light of these findings, the xanthine oxidase/methyl gallate 2 complex was simulated using the Desmond module of the Schrodinger suite molecular dynamics (MD) for 100 ns. Substantially stable receptor–ligand complexes were observed following 1 ns of MD simulation.

## Abstract 2

Seven components from the methanol extract of the aerial part of the endemic species *Helianthemum confertum* were isolated and identified for the first time.

## Graphical abstract

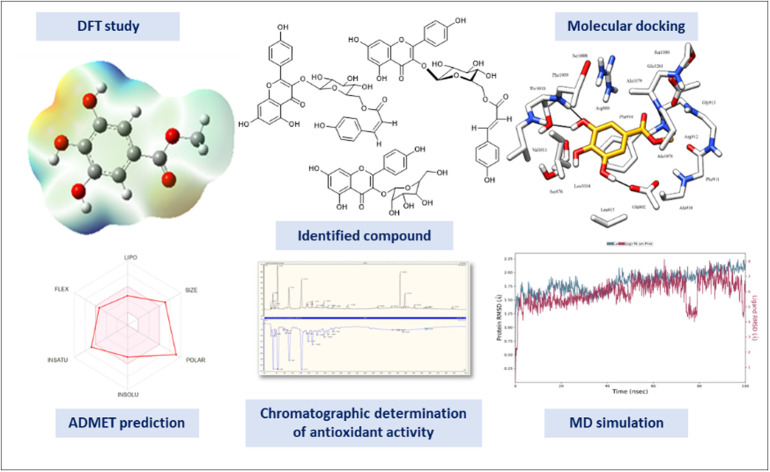

## Introduction

The 110 species that make up the genus *Helianthemum* are found mostly in the Northern Sahara, Central and Southern Europe, Russia, the Americas, and North Africa (including Algeria, Morocco, Tunisia, and Libya).^1^ They are members of the family Cistaceae. Not all species have the same range; for example, *H. songoricum* can be found in Central Asia, while *H. nummularium* and *H. oelandicum* can be found in Northern Europe.^2^ In recent decades, a number of these species have emerged as important medicinal plants with a wide range of uses in different nations. In Turkey, they treat conditions like constipation and gangrene.^3^ Traditional Mexican medicine uses them for gastrointestinal disorders like diarrhoea.^4^ Some countries use them for gastrointestinal issues, inflammation, ulcers, wound healing, parasites, microbes, analgesia, and vasodilation.^5,6^

According to earlier research on *Helianthemum* species, the polyphenolic chemicals contained in the plant structures are responsible for their high biological activity. Significant pharmacological effects have been demonstrated by scientific investigations involving these medications, which include analgesic,^7^ cytotoxic,^8^ antioxidant,^9,10^ antimicrobial,^6,9,11–14^ antiprotozoal,^15,16^ antiigiardial^4,17^ and ameobicidal.^16^

Ethnobotanical studies have shown that the genus *Helianthemum* has a long history of folk medicine usage for treating respiratory, hepatic, renal, arthritic, and inflammatory diseases, including pharyngeal inflammation and sore throats.^18–21^ These plants are considered useful therapies for a variety of gastrointestinal issues, including ulcers, dysentery, haemorrhoids, constipation, mucoid and bloody diarrhoea, intestinal worms, and microbial infections, these plants are thought to be useful therapies. Additionally, they alleviate stomach pain, serve as effective antiseptics and fever reducers, and receive high recommendations for treating skin conditions, disinfecting burns, wounds, and lesions, and treating snake and scorpion stings. They are also recommended for the treatment of cancer, allergies, gout, RA, anxiety, and other stressful mental and behavioural disorders.^22^

Little work has been devoted to the chemical composition of *Helianthemum* species and its essential oil.^23,24^ In the mid-1990s, a group led by F. Clazada conducted investigations that narrowed their emphasis to the species *H. glomeratum*.^15,25^

Some synonyms of *H. confertum* are: *H. ellipticum*, *H*. *confertum* Willk, and *Helianthemum confertum* Dunal.^26,27^ There have been no extensive reports on this species, which is native to the septentrional Sahara. Algeria has long used this plant to treat liver diseases,^28^ while Tunisia reports its effectivenses against skin infections.^29^

Our investigation into *H. confertum* uncovered a wealth of bioactive chemicals, including polyphenols and flavonoids, that possess antioxidant and antiproliferative effects.^30^ A chemoprotective action against doxorubicin-induced oxidative stress was also shown by this species.^28^

In order to evaluate the chemical makeup and biological activity of plants, numerous studies have shown that different extraction and solvent procedures are highly effective. Research into antioxidant activity have revealed that using methanol solvent in maceration and soxhlet procedures yields the best results for radical scavenging activities.^31^ In addition, a number of studies have shown that the most common solvents used to extract phenolic chemicals are methanol (or ethanol) and water, in various ratios of the two.^32–34^

We performed chemical tests on the ethyl acetate extract of *Helianthemum confertum* and isolated seven compounds. Liquid chromatography with a post-column reaction enabled the direct online determination of molecular species' radical scavenging capability. Using a variety of tests, such as ABTS˙^+^, TEAC, ORAC, and online HPLC-ABTS˙^+^, we tracked the progress of extracts and compounds with radical scavenging capacity.

To the best of our knowledge, this study represents the first phytochemical and *in silico* investigation of *Helianthemum confertum*. Elucidation of the isolated compound structures involved advanced techniques such as ESI-HRMS, molecular absorption spectroscopy, and extensive application of one- and two-dimensional NMR spectroscopy, along with comparison with literature data.

Therapeutic development requires an understanding of the toxicology, distribution, metabolism, absorption, and ADME/T characteristics of lead compounds. When it comes to *in silico* drug design, pharmacokinetic parameters and toxicity potential are invaluable tools.

Furthermore, molecular docking, frequently used in drug design, helps predict the binding sites of a ligand to a target protein. This study investigates the inhibitory effect of compounds against xanthine oxidase for the first time using *in silico* methods, contributing valuable insights to drug development. These computational approaches assist in understanding the chemical and toxic properties of lead compounds, predicting their interactions with specific receptors, and facilitating the selection of promising candidates for preclinical and clinical studies.

## Materials and methods

### Chemistry

#### General procedure

Ultraviolet spectra were recorded using a Shimadzu model UV-1700 spectrophotometer. Bruker model Avance 400 and AMX-500 spectrometers (Bruker BioSpin, Rheinstetten, Germany) obtained NMR spectra using standard pulse sequences, operating at 400 and 500 MHz for ^1^H and 100 and 125 MHz for ^13^C, respectively. MeOH-*d*_4_, DMSO-*d*_6_, or CDCl_3_ were used as solvents, with TMS as the internal standard. High resolution mass spectra (ESI-HRMS) were performed on an Agilent 6520 Accurate Mass Q-TOF (Agilent Corporation, Santa Clara, CA, USA) and a μ-QToF spectrometer (Bruker Daltonics, Wissembourg, France). Column chromatography (CC) was carried out with Si gel Fluka (cat. 60 737, 40–63 μm), and column fractions were monitored by TLC Si gel 60 F_254_, 0.2 mm, Macherey Nagel (cat. 818–333) by detection with a praying reagent (CH_3_CO_2_H/H_2_O/H_2_SO_4_; 80 : 16 : 4) followed by heating at 100 °C. Preparative TLC was carried out on Si gel 60 PF_254+366_ (20 cm × 20 cm, 1 mm thickness, Analtech cat. 02 014).

#### Plant material

M. Mohamed Benabdelhakem, director of the nature preservation agency, Bechar, collected the plant material from the Mougheul area in southwest Algeria and authenticated it.^35^ The Herbarium of the VERENBIOMOL research unit, University of Fréres Mentouri Constantine, has received a voucher specimen (HCC0512-MOG-ALG-60).

#### Extraction and isolation

Air dried area parts (2287.87 g) of *Helianthemum confertum* (Cistaceae) were macerated at room temperature with MeOH–H_2_O (80 : 20, v/v) for 72 hours, three times. After filtration, the filtrate was concentrated in vacuum (up to 35 °C) and dissolved in distilled H_2_O (900 mL) under magnetic stirring and then put in the refrigerator for one night. After filtration, the resulting solution was extracted successively with petroleum ether, chloroform, ethyl acetate, and *n*-butanol. The organic phases were dried with Na_2_SO_4_, filtered using common filter paper, and concentrated in a vacuum at 35 °C to obtain the following extracts: petroleum ether (0.01 g), chloroform (1.65 g), ethyl acetate (7.11 g), and *n*-butanol (35.07 g). A part of the ethyl acetate extract (6 g) was dissolved in 6 mL of MeOH and subjected to column chromatography on silica gel (60–200 mesh, 192 g) eluted with CHCl_3_/MeOH step gradients to yield 28 fractions (*F*_1_–*F*_28_) obtained by combining the eluates on the basis of TLC and analytical HPLC analysis. From the fractions *F*_9_ (217.7 mg), *F*_10_ (110.3 mg), *F*_13_ (202.2 mg), *F*_14_ (133.6 mg), and *F*_28_ (165 mg), aliquots were dissolved in methanol and submitted to a semi preparative HPLC separation using thermocolumn hypersil gold *C*_18_ (5 μm, 250 mm × 10 mm), with a mobile phase delivered at 5 mL min^−1^ consisting of a mixture of Milli-Q water containing 0.1% formic acid (solvent A) and acetonitrile containing 0.1% formic acid (solvent B). The gradient was as follows from *F*_9_ and *F*_10_: 0 min, 0% B; 30 min, 25% B; maintained during 7 min, to obtain: *para*-hydrobenzoic acid (1) and methyl gallate (2) (8 mg) as a mixture (50% and 50%, respectively), and protocatechuic acid (3) (1.8 mg). The gradient from *F*_13_, *F*_14_, and *F*_28_: 0 min, 0% B; 30 min, 25% B; maintained during 31 min. to obtain 4 compounds: astragalin (4) (1.2 mg), *trans*-tiliroside (5) (10 mg), and *cis*-tiliroside (6) (2.2 mg). This last compound was obtained in a mixture with *trans*-tiliroside (70% *trans*-tiliroside and 30% *cis*-tiliroside) and 3-oxo-α-ionol-β-d-glucopyranoside (7) (0.8 mg).

#### Solvents and chemicals

Ethyl acetate, formic acid, and methanol reagent grade were purchased from VWR (Fontenay-Sous-Bois, France); acetonitrile HPLC grade was purchased from fisher Scientific; and Milli-Q water (18.2 MΩ) was generated by the Millipore synergy system (Molsheim, France). Chemicals: 2,2′-azino-bis(3-ethylbenzthiazoline-6-sulfonic acid) diammonium salt (ABTS˙^+^), (C_18_H_24_N_6_O_6_S_4_) was purchased from Biochemica Applichem (Darmstadt, Germany); 2,2′-azobis(2 methylpropionamidine) dihydrochloride (AAPH), (C_8_H_18_N_6_ 2HCl); (+)-6-hydroxy-2,5,7,8 tetramethylchromane-2-carboxylic acid (trolox), (C_14_H_18_O_4_); fluorescein (C_20_H_12_O_5_), were purchased from Sigma-Aldrich (Steinheim, Germany); sodium chloride was purchased from VWR; potassium chloride and potassium persulfate were purchased from Prolabo (Paris, France); potassium dihydrogen phosphate, disodium hydrogen phosphate, and disodium dihydrogen phosphate were purchased from Merck (Darmstadt, Germany).

#### Determination of total phenolic content

We measured the total phenol content (TPC) of the ethyl acetate extract using the Folin–Ciocalteu method.^36^ Briefly, the extract was prepared at a concentration of 1 mg mL^−1^ in distilled water. 500 μL aliquots were transferred into a test tube, and 0.5 mL of Folin–Ciocalteu reagent (1 N) was added. The mixture was then allowed to stand for 4 min. Next, we added 1.5 mL of sodium carbonate solution (10%) to the mixture and used a UV-Vis spectrophotometer to read the absorbance at 765 nm after 90 minutes of dark incubation. All determinations were carried out in duplicates, and the TPC was expressed from a calibration curve as μg GAE mL^−1^ of extract.

### On-line HPLC-ABTS˙^+^ assay

The ABTS˙^+^ assay was based on the procedure described by Re^37^ and Siddhuraju.^38^

We dissolved ABTS (7 mM) in 20 mL of Milli-Q water, then added potassium persulfate (2.5 mM) to generate the radical cation ABTS˙^+^ overnight. The solution was left overnight at 4 °C protected from light exposure. This solution was used within 4 days and diluted in the phosphate buffer saline (PBS) solution (pH 7.4) in order to reach an absorbance of 1.2 at 412 nm. Phosphate Buffer Saline (PBS) was prepared by dissolving in Milli-Q water 80 g NaCl, 14.4 g Na_2_HPO_4_, 2.4 g KH_2_PO_4_, and 2 g KCl; the volume was completed with Milli-Q water to 1 L. The pH was adjusted to 7.4 using NaOH at 0.1 mol L^−1^. The radical cation ABTS˙^+^ solution (10 mL) was diluted in PBS (50 mL) and completed to 500 mL with Milli-Q water before use.

The diode array detector (DAD) was connected to a mixing tee, followed by a reaction coil (Peek, 20 m × 0.25 mm) loaded in a temperature controlled oven.

The post-column reaction is operated by delivering (0.5 mL min^−1^) the ABTS˙^+^ reagent with an Ultimate 3000 variable wavelength detector through the mixing tee. After the reaction coil, the flow passes through a second molecular absorption photometric detector set at 412 nm to detect the reduced form of the ABTS˙^+^ radical and thus a reduced absorbency. We present the result as a double chromatogram, with the upper part representing the phenolic compounds detected by their absorbance at 280 nm, and the lower part representing their free radical scavenging activity. A negative peak indicates that a compound with radical scavenging activity elutes out of the chromatographic column and reacts with the ABTS˙^+^ radical cation. The area of the chromatographic negative peak gives an indication of the radical-scavenging activity of the considered compound. The column used in the separation of EtOAc and CHCl_3_ extracts was a Kromasil C_18_ with a 5 μm particle size of 4.60 mm × 250 mm (column temperature: 25 °C). The mobile phase, delivered at 1 mL min^−1^, was composed of 0.1% formic acid in H_2_O Milli-Q (solvent A) and acetonitrile containing 1% formic acid (solvent B). The gradient was as follows: 0 min, 10% B; 10 min, 20% B; 20 min, 20% B; 50 min, 50% B; 55 min, 50% B; 56 min, 80% B; 66 min, 80% B; 67 min, 10% B, maintained during 13 min. Each phenolic compound was injected into the HPLC-ABTS˙^+^ and quantified by reference to its appropriate authentic standard by absorption at 280 nm, whereas the antioxidant potential was calculated as the concentration of trolox required to produce an equivalent negative peak area by absorption at 412 nm and expressed as trolox equivalent antioxidant capacity (TEAC) or μmol_TE_ mg^−1^.

### Oxygen radical absorbance capacity (ORAC)

The ORAC assay, developed and validated by Ou *et al.*^39^ was performed as described by Dávalos^40^ with minor modification by Volden.^41^ The assay measures the oxidative degradation of fluorescein by peroxyl radicals initiated by 2′,2-azobis(2-methylpropionamidine) dihydrochloride (AAPH) at 37 °C. Free radical scavenging molecules protect fluorescein from oxidative degradation and, until exhaustion, slow the reduction of the fluorescence signal by inducing latency. The area under the curve of the kinetics of fluorescence is directly proportional to the amount and effectiveness of the free radical scavengers present in a sample. The results are therefore expressed as the trolox equivalent (μmol_TE_ mg^−1^) of dry extract. The products are dissolved in a mixture of water and methanol (70/30) at 1 mg mL^−1^ (1000 ppm) and then have to be diluted with water (between 25 and 500 ppm) before being placed in triplicate in 96 wells micro-plates up to 10 μL per well. A trolox standard range between 25 and 500 μmol L^−1^ was also conducted in triplicate. An aqueous solution of 150 μL fluorescein (8.5 × 10^−6^ mol L^−1^) was added per well. An automatic dispenser then permits the initiation of the reaction by the addition of AAPH (30 μL, 153 μmol L^−1^) to each well from the initiation of the generation of radicals by the addition of AAPH, the intensity of fluorescence emitted is measured every 5 min for 2 h with a wavelength of excitation between 400 and 600 nm.

### Trolox equivalent antioxidant capacity (TEAC)

The TEAC method is a spectrophotometric technique based on the use of a stable coloured (blue-green) cationic radical, ABTS˙^+^ (2,2′-Azino-bis-(3ethylbenthioazoline-acid 6-sulfonic acid), which discolours when reduced by an antioxidant following electron transfer. A reference molecule, trolox ((+)-6-hydroxy-2,5,7,8-tetramethylchromane-acid. 2-carboxilic), is used in order to be able to compare the samples between them. The ABTS solution (Biochemica Apllichem, Darmstadt, Germany) is prepared by dissolving it at 7.5 mmol L^−1^ in Milli-Q water in the presence of 2.5 mmol L^−1^ of potassium persulfate, generating the radical ABTS˙^+^ cation by oxidation. A standard range of trolox (Sigma-Aldrich, Steinheim, Germany) (25; 50; 75; 100; 125; 150; 400 ppm) is prepared in Milli-Q water from a stock solution at 1000 ppm in a Milli-Q water/methanol mixture (50/50) and allows the expression of sample results in μmole Trolox equivalent/g of dry extract. The products were dissolved in a Milli-Q water/methanol (70/30) mixture at 1 mg mL^−1^, then diluted in Milli-Q water from 25 to 400 ppm. A volume of 10 μL of sample or range point (25 to 400 ppm) was deposited in triplicate in a 96-well plate, to which 200 μL of ABTS˙^+^ solution were added (the ABTS solution prepared is diluted in the 2% PBS solution (1x). The plate is then incubated at 37 °C for 10 minutes before reading the absorbance at 734 nm by a fluorescence reader (Varioskan Thermo Scientific).^42^

### Preparation of the PBS solution (phosphate buffer saline) 10 x

Add 80 g of sodium chloride (NaCl), 2 g of potassium chloride (KCl), 14.4 g of dibasic sodium phosphate (Na_2_HPO_4_), and 2.4 g of potassium phosphate (KH_2_PO_4_). Complete the volume of the mixture to 1 L with Milli-Q water (18.2 MΩ). We adjusted the pH to 7.4 using 0.1 mol L^−1^ of NaOH.

### Computational study

#### Molecular docking

The X-ray crystal structure of the xanthine oxidase–hypoxanthine complex (PDB ID: 3NRZ) was obtained from the Protein Data Bank,^43^ and it was prepared with the Protein Preparation Wizard in Schrodinger Suites. The three-dimensional structures of the derivatives were constructed using Maestro software and prepared with Ligprep using the OPLS3e force field.^44^

The final prepared PDB file of the protein and identified compounds were submitted in order to run the docking process. Docking studies were performed by Glide software^45^ at extra precision.^46^ Output files of docked compounds along with the xanthine oxidase protein were visualized on Chimaera software.^47^

#### Molecular dynamics simulation

We performed a Molecular Dynamics (MD) simulation using Desmond Academic Software-2022. The best docking complexes for the identified compounds from the biological experiments were taken as the initial coordinates for MD simulations. A 10 Å cubic water box employing the TIP3P water model was used for the solvation of the system with the OPLS_2005 force field. Sodium ions were added as counter ions to neutralise the systems. We have then subjected the system to minimization using an energy gradient convergence threshold of 1 kcal mol^−1^ Å^−1^ and pre-equilibration using the default six-step relaxation protocol implemented in Desmond. We perform the first two steps of minimization, one with the solute under restraint and the other without it. Further, steps three to six are short MD simulations of 12 ps, 12 ps, and 24 ps each using the NPT ensemble at 10, 10, 300, and 300 K, respectively. Subsequently, a 100 nanosecond MD simulation production run was performed. The remaining parameters were kept at the Desmond default values. We have visualised the protein–ligand complexes and analysed the MD trajectory using Maestro. The detailed analysis were performed using the Simulation Event Analysis tool from Desmond.^48^

#### Density functional theory (DFT) analysis

Molecular geometry: the gas phase structure optimization of isolated compounds is optimized using DFT at the B3LYP method,^49^ with the basis set of 6-31G (d, p) implemented by Gaussian 09 package.^50^ We calculate the highest occupied molecular orbital (HOMO)^51^ and lowest un-occupied molecular orbital (LUMO), as well as the energy gap and chemical reactivity descriptors, using the DFT/B3LYP/6-31G (d, p) method. We transitioned our chemical structure modeling process from Chemdraw 3D software (Chem-Draw, 2019) to GaussView 06 software, importing MDL mol files containing the 3D structures of our studied molecules isolated from *Helianthemum confertum*. Theoretical calculations were then conducted using Gaussian 09 in the gas phase, employing the DFT method with the 6-31G (d, p) basis set and utilizing the B3LYP functional.^49–51^ These calculations aimed to determine the most stable conformation and elucidate the molecular electrostatic potential (MEP), as well as global reactivity indices.

The literature proposes various reactivity indices as global properties. It's important to note that among these, the chemical potential (*μ*) and hardness (*η*) stand out as two fundamental descriptors, from which other global indices such as the global softness (*S*) or the global electrophilic index (*ω*) can be derived. Here, we computed the global indices of our molecular system using the B3LYP/6-31G (d, p) level of DFT theory, extracting them from the energy levels of the Highest Occupied Molecular Orbital (HOMO) and Lowest Unoccupied Molecular Orbital (LUMO) according to the following equations:

Electrophilicity index *ω*^52^1
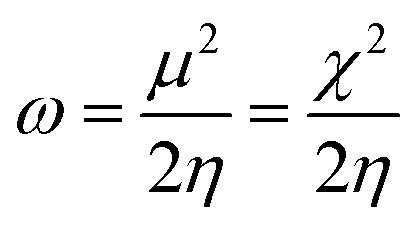


To determine this electrophilicity, we calculated the chemical potential *μ*,^53^ which is a negative value and chemical hardness *η*^54^2
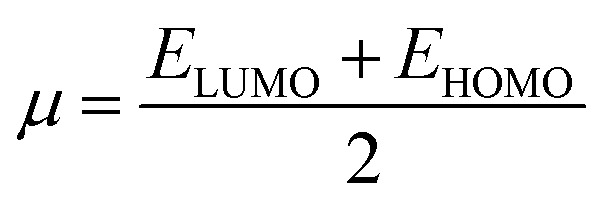
3
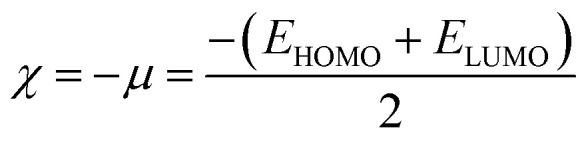
4
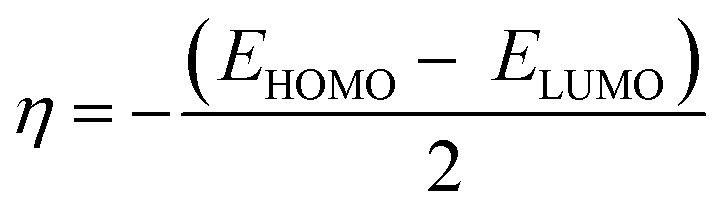


Molecular softness *S*,^55^ nucleophilicity index *N*.^56^5
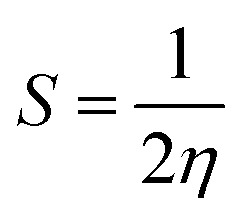

6 *N* = *E*_HOMO_ − *E*_HOMO_ (TCE)

### Pharmacokinetics analysis (ADME/T)

Various *in silico* methods aim to predict ADME/T parameters based on the molecular structure of compounds. One significant contribution in this field was made by Lipinski *et al.*, who studied orally active compounds to establish physicochemical ranges that increase the probability of a compound being an oral drug.

This approach, known as the Rule-of-Five, established a correlation between pharmacokinetic and physicochemical parameters.^57^ Regarding this part of the work, we used the SwissADME web tool *via* the link: https://www.swissadme.ch/, which provides free access to a pool of quick yet reliable predictive models for physicochemical properties, pharmacokinetics, drug-likeness, and medicinal chemistry friendliness, including in-house effective techniques like the BOILED-Egg, iLOGP, and Bioavailability Radar.^58^ The Drug Likeness Score (DLS) result was determined using the Molsoft web tool *via* the link: https://www.molsoft.com.^59^

## Results and discussion

### Chemistry

#### Total phenolic

Table 1 shows the amount of secondary metabolites in the crude extract. Total phenolic content was expressed in mg AGE mL^−1^ of extract by using a calibration curve (phenol: *y* = 0.0002*x* + 0.019, *R*^2^ = 0.9976). Our results show that the ethyl acetate extract is rich in polyphenols, with a total phenol content (388.33 ± 0.02 mg AGE mL^−1^).

**Table 1:** Total phenolic content of ethyl acetate extract (results expressed as mean ± SD, *n* = 3)

Extract	Total phenolics (mg AGE mL^−1^)
Ethyl acetate	388.33 ± 0.02

As an alternative, it should be noted that there is a relationship between polyphenol concentration and antioxidant activity. This highlights the antioxidant effectiveness of polyphenols, showing that they can inhibit free radical production and counteract macromolecule oxidation.

### Isolation and structure elucidation of compounds

**Fig. 1:**
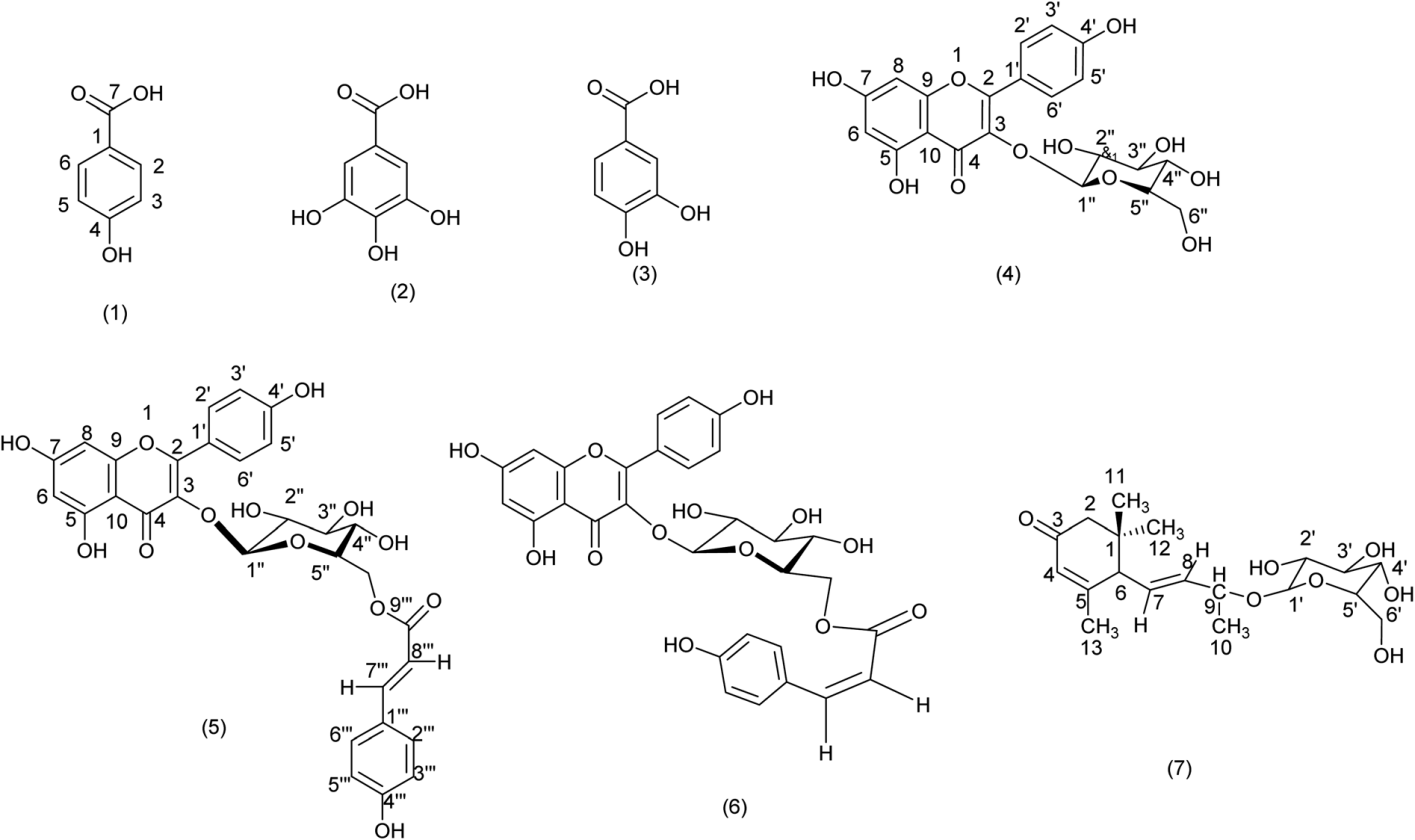
Structures of compounds (1–7) isolated from *Helianthemum confertum*.

Using semi-preparative reverse phase HPLC, seven compounds were extracted from the aerial parts of *Helianthemum confertum*: a mixture (50–50%) of *para*-hydroxybenzoic acid (1),^60^ and methyl gallate (2),^61^ protocatechuic acid (3),^62^ astragalin (4),^63^*trans*-tiliroside (5),^64,65^*cis*-tiliroside (6)^64,65^ contaminated by *trans*-tiliroside, and 3-oxo-α-ionol-β-d-glucopyranoside (7)^66^ (Fig. 1).

Spectral investigation, including ESI-HRMS, UV, and NMR experiments (^1^H, ^13^C, DEPT, DOSY, COSY, NOESY, HSQC, and HMBC), as well as comparison with spectroscopic data given in the literature, were used to identify the compounds. We are currently unaware of any other compounds in the *Helianthemum* genus that can be compared to compounds 2 and 7. Additionally, no chemical has ever been reported before in this species; all of the identified compounds are novel. Compounds 2 and 7 are particularly noteworthy additions to the *Helianthemum* genus, although these substances have been found in other species and in the Cistaceae family as well.^61,63,64,66^

*Para*-hydroxybenzoic acid (1): HRESI-MS (+): *m/z* 139.03814 [M + H]^+^; formula: C_7_H_6_O_3_; ^1^H-NMR (400 MHz, CD_3_OD) *δ*_H_ (ppm) 7.89 (1H, *d*, *J* = 8.8 Hz, H-2, H-6), 6.83 (^1^H, *d*, *J* = 8.8 Hz, H-3, H-5); ^13^C-NMR (100 MHz, CD_3_OD) *δ*_C_ (ppm) 168.76 (C-7), 161.94 (C-4), 131.64 (C-2, C-6), 121.35 (C-1), 114.68 (C-3, C-5).

Methyl gallate (2): HRESI-MS (+): *m/z* 185.04359 [M + H]^+^ accurate mass 184.03632; formula: C_8_H_8_O_5_; ^1^H-NMR (400 MHz, CD_3_OD) *δ*_H_ (ppm) 7.07 (^1^H, s, H-2, H-6), 3.82 (3H, s, –OCH_3_); ^13^C-NMR (100 MHz, CD_3_OD) *δ*_C_ (ppm) 167.68 (C-7), 145.10 (C-3, C-5), 138.37 (C-4), 120.13 (C-1), 108.74 (C-2, C-6), 50.93 (-OCH_3_).

Protocatechuic acid (3): HRESI-MS (+): *m/z* 155.03358 [M + H]^+^, accurate mass 154.0263; formula: C_7_H_6_O_4_; ^1^H-NMR (400 MHz, CD_3_OD) *δ*_H_ (ppm) 7.5 (^1^H, *d*, *J* = 2 Hz, H-2), 7.48 (^1^H, dd, *J* = 8.2, 2 Hz, H-6), 6.85 (^1^H, *d*, *J* = 8.2 Hz, H-5); ^13^C-NMR (100 MHz, CD_3_OD) *δ*_C_ (ppm) 169.30 (C-7), 150.14 (C-4), 144.57 (C-3), 122.88 (C-1), 121.71 (C-6), 116.52 (C-2), 114.63 (C-5).

Astragalin (4): HRESI-MS (+): *m/z* 449.10813 [M + H]^+^, accurate mass: 448.10056, formula: C_21_H_20_O_11_; ^1^H-NMR (400 MHz, CD_3_OD) *δ*_H_ (ppm) aglycone: 7.95 (2H, *d*, *J* = 8.7 Hz, H-2′, H-6′), 6.79 (2H, *d*, *J* = 8.7 Hz, H-3′, H-5′), 6.30 (^1^H, *d*, *J* = 2.0 Hz, H-8), 6.10 (1H, *d*, *J* = 2.0 Hz, H-6), sugar moiety: 5.13 (1H, *d*, *J* = 7.3 Hz, H-1′′), 3.59 (^1^H, dd, *J* = 11.5, 2.1 Hz, H-6′′a), 3.43 (^1^H, dd, *J* = 12.0, 5.7 Hz, H-6′′b), 3.34 (^1^H, m, H-2′′), 3.29 (^1^H, m, H-3′′), 3.18 (^1^H, t, *J* = 8 Hz, H-4′′), 3.10 (^1^H, m, H-5′′); ^13^C-NMR (100 MHz, CD_3_OD) *δ*_C_ (ppm) aglycone: 178.15 (C-4), 164.57 (C-7), 161.66 (C-5), 160.17 (C-4′), 157.79 (C-2), 157.12 (C-9), 134.12 (C-3), 131.04 (C-2′, C-6′), 121.43 (C-1′), 114.73 (C-3′, C-5′), 104.39 (C-10) 98.56 (C-6), 94.98 (C-8), sugar moiety: 102.81 (C-1′′), 76.99 (−5′′), 76.68 (C-3′′), 74.36 (C-2′′), 70.00 (C-4′′), 61.29 (C-6′′).

*trans*-Tiliroside (5): yellow powder, HRESI-MS (+): *m/z* 617.12741 [M + Na]^+^, 595.14507 [M + H]^+^, formula: C_30_H_26_O_13_; ^1^H-NMR (400 MHz, CD_3_OD) *δ*_H_ (ppm) aglycone: 8.01 (2H, *d*, *J* = 8.3 Hz, H-2′, H-6′), 6.84 (2H, *d*, *J* = 8.3 Hz, H-3′, H-5′), 6.28 (_1_H, *d*, *J* = 2.2 Hz, H-8), 6.12 (^1^H, *d*, *J* = 2.2 Hz, H-6), sugar moiety: 5.21 (^1^H, *d*, *J* = 7.4 Hz, H-1′′), 4.32 (^1^H, dd, *J* = 10.0, 2.0 Hz, H-6′′a), 4.19 (^1^H, dd, *J* = 6.5, 5.2 Hz, H-6′′b), 3.48 (^1^H, t, *J* = 5.2 Hz, H-2′′), 3.46 (^1^H, m, H-5′′), 3.44 (^1^H, 1m, H-3′′), 3.34 (^1^H, m, H-4′′), *p*-coumaroyl moiety: 7.43 (^1^H, *d*, *J* = 16.0 Hz, H-7′′′), 7.35 (2H, *d*, *J* = 8.7 Hz, H-2′′′,H-6′′′), 6.84 (2H, *d*, *J* = 8.7 Hz, H-3′′′, H-5′′′), 6.08 (^1^H, *d*, *J* = 16.0 Hz, H-8′′′); ^13^C-NMR (100 MHz, CD_3_OD) *δ*_C_ (ppm) aglycone: 177.69 (C-4), 1767.44 (C-7), 161.42 (C-5), 160.13 (C-4′), 157.60 (C-9), 157,27 (C-2), 133.73 (C-3), 130.75 (C-2′, C-6′), 121.40 (C-1′), 114,65 (C-3′, C-5′), 103.38 (C-10), 99.56 (C-6), 94.12 (C-8), sugar moiety: 102.92 (C-1′′), 76.65 (C-3′′), 74.41 (C-5′′), 74.32 (C-2′′), 70.30 (C-4′′), 62.94 (C-6′′), *p*-coumaroyl moiety: 167.45 (C-9′′′), 157.25 (C-4′′′), 145.22 (C-7′′′), 129.82 (C-2′′′, C-6′′′), 125.66 (C-1′′′), 115.45 (C-3′′′, C-5′′′), 113.30 (C-8′′′).

*cis*-Tiliroside (6): This compound was obtained as a mixture with *trans*-tiliroside (5) (70% *trans*-tiliroside- 30% *cis*-tiliroside). Yellow powder, HRESI-MS (+): *m/z* 595.14494 [M + H]^+^, formula: C_30_H_26_O_13_; ^1^H-NMR (400 MHz, CD_3_OD) *δ*_H_ (ppm) aglycone: 7.96 (2H, d, *J* = 8.7 Hz, H-2′,H-6′), 6.60 (2H, d, *J* = 8.7 Hz, H-3′, H-5′), 6.31 (^1^H, *d*, *J* = 2 Hz, H-8), 6.11 (^1^H, *d*, *J* = 2 Hz, H-6), sugar moiety: 5.15 (^1^H, *d*, *J* = 7.4 Hz, H-1), 4.20 (^1^H, dd, *J* = 9.6, 2.4 Hz, H-6′′a), 4.09 (^1^H, dd, *J* = 6.4, 5.2 Hz, H-6′′b), 3.36 (^1^H, m, H-2′′), 3.35 (^1^H, m, H-5′′), 3.22 (^1^H, m, H-3′′), 3.24 (^1^H, m, H-4′′), *p*-coumaroyl moiety: 7.41 (2H, d, *J* = 8.8 Hz, H-2′′′, H-6′′′), 6.58 (2H, *d*, *J* = 8.8 Hz, H-3′′′, H-5′′′), 6.62 (^1^H, *d*, *J* = 12.7 Hz, H-7′′′), 5.41 (^1^H, *d*, *J* = 12.7 Hz, H-8′′′); ^13^C-NMR (100 MHz, CD_3_OD) *δ*_C_ (ppm) aglycone: 177.10 (C-4), 164.54 (C-7), 161.61 (C-5), 159.79 (C-4′), 157.98 (C-2), 157.05 (C-9), 133.78 (C-3), 130.80 (C-2′, C-6′), 121.36 (C-1′), 115.40 (C-3′, C-5′), 104.23 (C-10), 98.61 (C-6), 93.41 (C-8), sugar moiety: 102.52 (C-1′′), 76.66 (C-3′′), 74.43 (C-5′′), 74.33 (C-2′′), 70.34 (C-4′′), 62.89 (C-6′′), *p*-coumaroyl moiety: 167.38 (C-9′′′), 159.79 (C-4′′′), 132.35 (C-2′′′, C-6′′′), 129.78 (C-3′′′, C-5′′′), 125.72 (C-1′′′), 114.66 (C-7′′′), 114.33 (C-8′′′).

3-oxo-α-ionol-β-d-glucopyranoside (7): this compound was obtained as a mixture with *trans*-tiliroside (5) (60.71% 3-oxo-α-ionol-β-d-glucopyranoside – 39.29% *trans*-tiliroside). (HRESI (+): *m/z* 393,18 772 [M + Na]^+^, *m/z* 370.1985 [M + H]^+^, accurate mass *m/z* 370.19915, formula: C_19_H_30_O_7_; ^1^H-NMR (500 MHz, CD_3_OD) *δ*_H_ (ppm) aglycone: 5.90 (^1^H, s, H-4), 5.79 (^1^H, dd, *J* = 15.5, 8.8 Hz, H-8), 5.67 (^1^H, dd, *J* = 15.5, 7.7 Hz, H-7), 4.41 (^1^H, m, H-9), 2.70 (^1^H, *d*, *J* = 7.7 Hz, H-6), 2.45 (^1^H, *d*, *J* = 16.8 Hz, H-2a), 2.07 (^1^H, *d*, *J* = 16.8 Hz, H-2b), 1.95 (3H, s, H_3_-13), 1.31 (3H, *d*, *J* = 6.4 Hz, H_3_-10), 1.05 (3H, s, H_3_-12), 1.02 (3H, s, H_3_-11), sugar moiety: 4.37 (^1^H, *d*, *J* = 7.7 Hz, H-1′), 3.84 (^1^H, dd, *J* = 12.0, 2.0 Hz, H-6′a), 3.68 (^1^H, dd, *J* = 12.0, 5.2 Hz, H-6′b), 3.34 (^1^H, m, H-3′), 3.30 (^1^H, m, H-4′), 3.23 (^1^H, m, H-5′), 3.18 (^1^H, m, H-2′); ^13^C-NMR (125 MHz, CD_3_OD) *δ*_C_ (ppm) aglycone: 202.15 (C-3), 166.77 (C-5), 138.33 (C-8), 129.07 (C-7), 126.26 (C-4), 77.31 (C-9), 56.92 (C-6), 48.45 (C-2), 37.30 (C-1), 28.23 (C-12), 27.76 (C-11), 23.95 (C-13), 21.26 (C-10), sugar moiety: 102.60 (C-1′), 78.24 (C-5′), 78.11 (C-3′), 75.44 (C-2′), 71.64 (C-4′), 62.81 (C-6′).

### Identification of chromatographic peaks and antioxidant activity of plant extracts and pure compounds

**Fig. 2:**
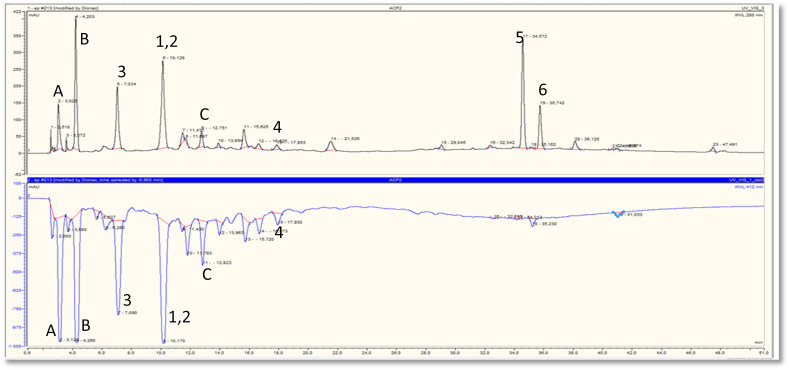
Chromatographic determination of antioxidant compounds (upper chromatogram) and their corresponding antioxidant activity (lower chromatogram) in ethyl acetate extract of *H. confertum*. *Para*-hydroxybenzoic acid (1), methyl gallate (2), protocatechuic acid (3), astragalin (4), *trans*-tiliroside (5), *cis*-tiliroside (6), unidentified compounds (A, B and C).

**Fig. 3:**
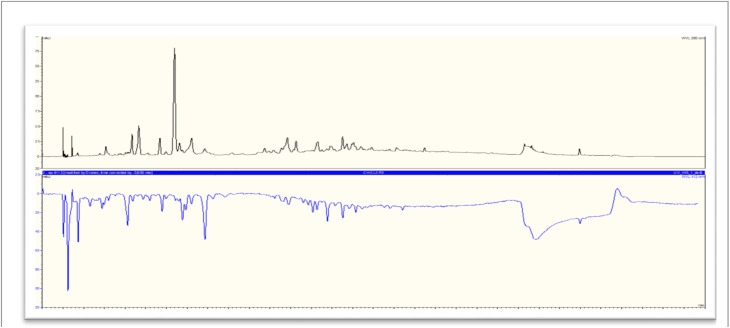
Chromatographic determination of antioxidant compounds (upper chromatogram) and their corresponding antioxidant activity (lower chromatogram) in chloroform extract of *H. confertum*.

The on-line HPLC-ABTS˙^+^ profile of the ethyl acetate and chloroform extracts of *H. confertum* is reported in Fig. 2 and 3. This profile showed a wealth of the extract in phenolic compounds detected by their absorbency at 280 nm. High temperatures, bright sunshine, dryness, and salinity are some of the severe weather conditions that could be contributing factors to this occurrence. Under these circumstances, secondary metabolites like polyphenols are biosynthesized.^67^ Following chromatographic separation and purification, the separated chemicals were re-injected into the HPLC-ABTS˙^+^ system under identical circumstances as the extracts, and chromatographic peaks were identified. Tables 2 and 3 show that the compounds with relatively high radical scavenging capacity were *para*-hydroxybenzoic acid and methyl gallate (1 and 2), protocatechuic acid (3), and astragalin (4), while Table 3 shows that the mixture of these two compounds was the most active at 828 mAU. Table 3 shows that the ethyl acetate extract has a total of 40.06 percent antioxidant activity from the three phenolic acids 1, 2, and 3, as well as the flavonoid glycoside astragalin (4). Unfortunately, compounds A, B, and C, which also exhibited high antioxidant activity, were not able to be isolated in their pure forms and were thus not identified. According to Table 3, these three components accounted for 42.30% of the antioxidant activity of the ethyl acetate extract. Fig. 2 shows that despite having a very high molecular absorbance on the upper chromatogram, *trans*- and *cis*-tiliroside (5 and 6) had very little radical scavenging capacity. Off-line ABTS, ORAC, and TEAC tests validated these findings. Table 2 shows that the two *Helianthemum confertum* extracts, EtOAc and CHCl_3_, had similar free radical scavenger capacities, with TEAC values of 649 and 410 μMol_TE_ mg^−1^, respectively, as confirmed by the findings of the off-line TEAC test.

**Table 2:** Free radical scavenging activities of the pure compounds and extracts of *H. confertum* based on ABTS, Oxygen Radical Absorbance Capacity (ORAC) and trolox equivalent antioxidant capacity (TEAC) assays. (Results expressed as mean ± SD, *n* = 3)

Peaks	Pure compounds or extracts	ABTS (μg_TE_ mL^−1^)	Radical scavenging activity (mAU)	ORAC (μMol_TE_ mg^−1^)	TEAC (μMol_TE_ mg^−1^)
	EtOAc extract	—	—	557.9 ± 11.04	649.4 ± 0.02
	CHCl_3_ extract	—	—	363.8 ± 15.45	410.06 ± 0.04
1 and 2	*para*-Hydroxybenzoic acid and methyl gallate	153.93	637	156.82 ± 33.02	387.97 ± 17.49
3	Protocatechuic acid	137.39	569	158.21 ± 20.39	185.58 ± 9.01
4	Astragalin	1.48	13	300.006 ± 82.71	144.69 ± 15.36

**Table 3:** Free radical scavenging activities of the phenolic compounds in the EtOAc extract of *H. confertum* based on ABTS˙^+^ and their participation (%) in total antioxidant capacities with ABTS assay

Peaks	Compounds	ABTS (μg_fTE_ mL^−1^)	Radical scavenging activity (mAU)	Scavenging activity percent (%)
A	Not identified	204.17	842	16.80
B	Not identified	209.50	864	20.36
1 and 2	*para*-Hydroxybenzoic acid and methyl gallate	200.84	828	23.00
3	Protocatechuic acid	155.19	642	15.09
4	Astragalin	11.48	54	1.97
C	Not identified	71.70	300	5.14

This may be in relation to the strongest amount of phenolic and flavonoid compounds in these extracts. The off-line ABTS assay of the isolated compounds also showed that *para*-hydroxybenzoic acid (1), methyl gallate (2), protocatechuic acid (3), and astragalin (4) were the most potent antioxidant with on-line ABTS assay values: 828, 642 and 54 mAU respectively (Table 3), off-line ABTS values: 637, 569, and 13 mAU respectively (Table 2). ORAC essay values: 157, 158 and 300 μmol_TE_ mg^−1^, respectively (Table 2), and TEAC assay values: 388, 185, and 145 μmol_TE_ mg^−1^ (Table 2). The higher activity of the mixture of *para*-hydroxybenzoic acid and methyl gallate (1 and 2) and protocatechuic acid (3) in comparison with astragalin (4) may be due to the lack of glycosylation, which has been found to diminish the radical scavenging activity.^68^ The values of on-line or off-line ABTS of the mixture of *para*-hydroxybenzoic acid and methyl gallate (1 and 2) and protocatechuic acid (3) are comparable, and this can be due to the similarity of the structures.

This plant may have traditional medicinal use in Algeria due to its strong antioxidant potential and the presence of *trans*-tiliroside, which has been shown to have several biological effects.^69,70^ Furthermore, it should be mentioned that no report regarding the potential ethnomedical applications of this species has been published thus far.

Numerous authors^71–73^ have indicated that the antioxidant activity of plant extracts is contingent upon the quantity of phenolic compounds present. Indeed, this significant antioxidant efficacy may be linked to the number and positioning of hydroxyl groups^74^ within the molecule, as well as the nature of the phenolic compounds, which can potentially exhibit synergistic effects.^75^

### *In silico* study

#### Molecular docking

The xanthine oxidase enzyme is essential for purine metabolism, which is how the body breaks down purines, a type of chemical component included in many foods. Cao *et al.*'s research indicates that hypoxanthine undergoes hydroxylation at the C-2 position by xanthine oxidase (XO) to form xanthine, which is then further converted to uric acid through a reductive half-reaction.^76^ This process involves the oxidative hydroxylation of the substrate at the molybdenum (Mo) center, which generates reactive oxygen species (ROS). Therefore, inhibiting xanthine oxidase could be a viable approach to managing diseases that lead to the accumulation of uric acid and the associated ROS production. Such a strategy could be useful in both preventing and treating these conditions. To understand the interactions between extracted compounds and the important xanthine oxidase enzyme, molecular docking was performed. The structure derived from X-rays of the hypoxanthine complex with xanthine oxidase PDB codes 3NRZ was selected for molecular docking studies due to good parameters for experimental resolution (1.8 Å). We evaluated the accuracy of the docking protocol by re-docking hypoxanthine into the active site of the XO enzyme.

**Fig. 4:**
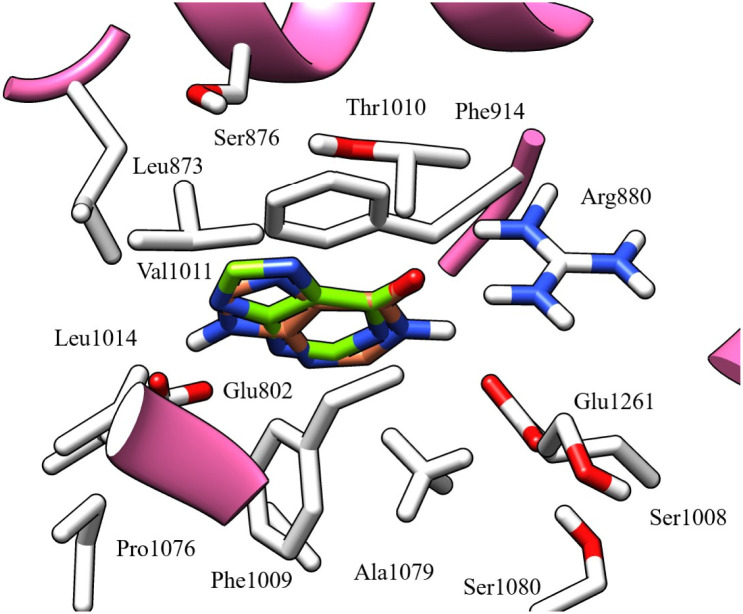
Docked and co-crystalized hypoxanthine in xanthine oxidase enzyme after self-docking calculation.

Fig. 4 shows the results, which show that the docked hypoxanthine and co-crystallized ligand were almost exactly where they should have been in the receptor, with an RMSD of 1.20 Å. These findings confirmed the validity of the docking protocol using the Extra Precision (XP) Glide scoring function and were obtained in the absence of water molecules.

All compounds were docked into the active site of the XO enzyme, and most of them exhibited interesting stability inside the cavity with docking scores within the range of −6.40 to −6.90 kcal mol^−1^, higher than the score of the co-crystallized ligand except the 3-oxo-α-ionol-β-d-glucopyranoside compound, as presented in Table 4.

**Table 4:** Docking score of docked ligands and the reference ligand ‘hypoxanthine’ against XO enzyme

Compound	Docking score (kcal mol^−1^)
*para*-Hydroxybenzoic acid	−6.44
Methyl gallate	**−6.80**
Protocatechuic acid	−6.40
Astragalin	−6.53
*trans*-Tiliroside	−6.68
*cis*-Tiliroside	−6.50
3-Oxo-α-ionol-β-d-glucopyranoside	−5.01
Hypoxanthine	−6.07

**Fig. 5:**
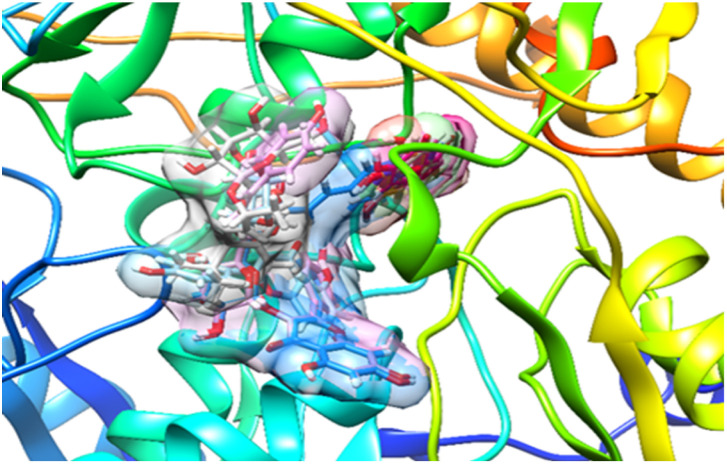
Super-imposition of docked ligands in the active site of XO enzyme.

Moreover, the docked compounds remained in the active site pocket of the XO enzyme (Fig. 5); therefore, we were interested in studying the interaction of compounds.

The binding interaction of the molecular docking results of studied ligands and hypoxanthine were reported in Table 5.

**Table 5:** Analysis of binding interaction of extract compound and the reference ligand against xanthine oxidase enzyme

Compound	Hydrogen bond	Hydrophobic interaction
*para*-Hydroxybenzoic acid	Glu162, Ala1079	Phe914, Phe1005, Ala1078, Ala1079, Pro1076, Leu873, Leu1014, Val1011
Methyl gallate	Glu802, Thr1010	Phe914, Phe1009, Ala910, Ala1078, Ala1079, Pro1076, Leu873, Leu1014, Met770, Val1011
Protocatechuic acid	Glu802, Glu1261, Ala1079	Phe914, Phe1009, Ala1078, Ala1079, Pro1076, Leu873, Leu1014, Phe1005, Val1011
Astragalin	Ser876	Pro1076, Leu873, Leu1014, Val1011, Leu648, Phe649, Met770, His875
*trans*-Tiliroside	Leu648, Asn1073, Lys771	Pro1076, Phe1013, Leu648, Leu1014, Leu873, Val1011, Phe1009, Phe775, Ala1078, Ala1079
*cis*-Tiliroside	Glu879, Lys771, Asn1073, Leu648	Pro1076, Phe1142, Leu648, Leu1014, Leu873, Val1011, Phe649, Phe775, His875, Met770
3-Oxo-α-ionol-β-d-glucopyranoside	Glu879, Lys771	Pro1012, Phe1013, Leu648, Leu1014, Val1011, Leu648, Phe649, Phe1142, His875
Hypoxanthine	Glu802, Thr1010, Arg880	Phe914, Phe1009, Ala910, Ala1078, Ala1079, Pro1076, Leu873, Leu1014, Met770, Val1011

**Fig. 6:**
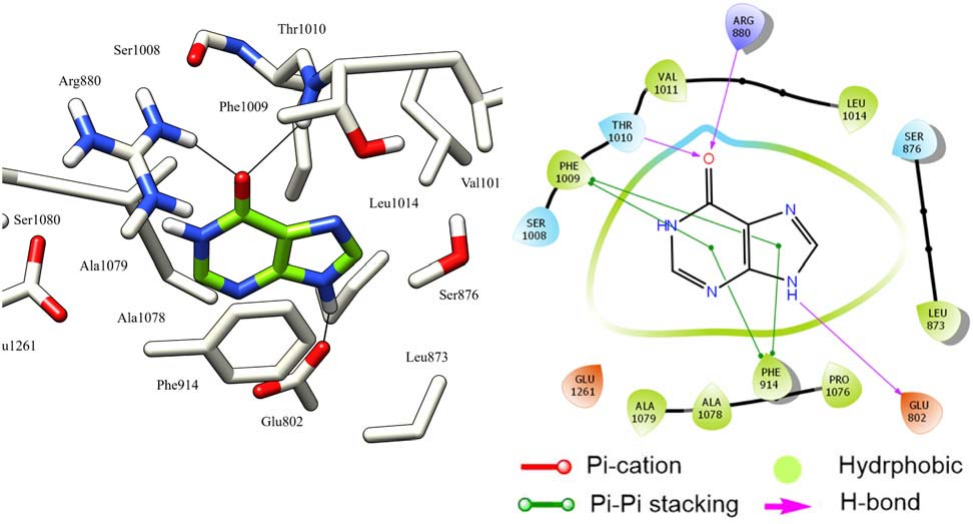
3D left and 2D right binding disposition of hypoxanthine after docking calculations in the active site of XO enzyme. The amino acid residues were shown as stick model and H-bonds were shown as black lines.

The reference ligand hypoxanthine formed three hydrogen bonds with Glu802, Thr1010, and Arg880 residues. Moreover, the two aromatic rings of hypoxanthine are involved in Pi–Pi stacking interactions with Phe1009 and Phe914 residues. These interactions suggest that these residues possess aromatic π-electron-rich properties, while the Phe914, Phe1009, Ala910, Ala1078, Ala1079, Pro1076, Leu873, Leu1014, Met770, and Val1011 residues form hydrophobic interactions (Fig. 6).

**Fig. 7:**
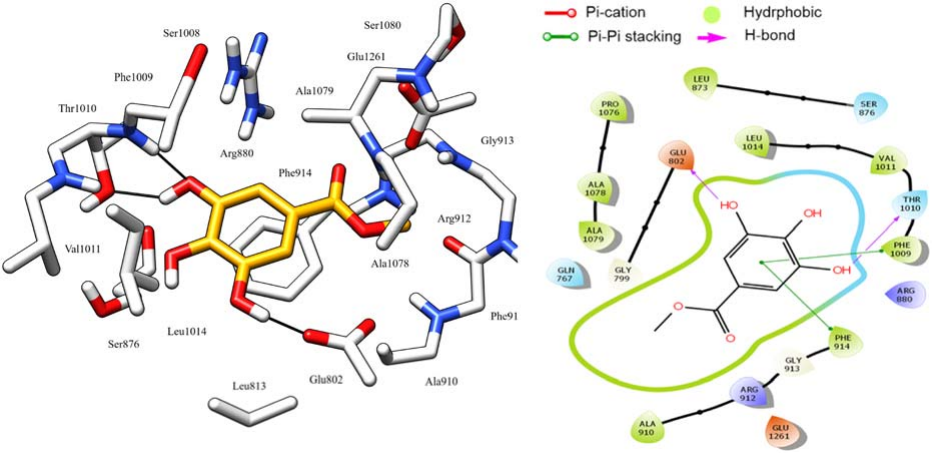
3D left and 2D right binding disposition of methyl gallate after docking calculations in the active site of XO enzyme. The amino acid residues were shown as stick model and H-bonds were shown as black lines.

Most docked compounds effectively formed strong hydrogen bonds and hydrophobic interactions with the specific residues responsible for inhibition of the XO enzyme, as shown in Table 5. Notably, the most stable compound, methyl gallate, formed three hydrogen bonds through its hydroxyl groups on the aromatic ring. Two of these bonds were established with the NH and side chain OH of the Thr1010 residue, while the third bond formed with the Glu802 residue. In addition, methyl gallate demonstrated a strong electrostatic attraction force with Ser876 and two Pi–Pi stacking interactions between its aromatic ring and the aromatic rings of two other residues, Phe914 and Phe1009. Furthermore, the compound is involved in hydrophobic interactions with the active site residues, contributing to XO enzyme inhibition (Fig. 7).

**Fig. 8:**
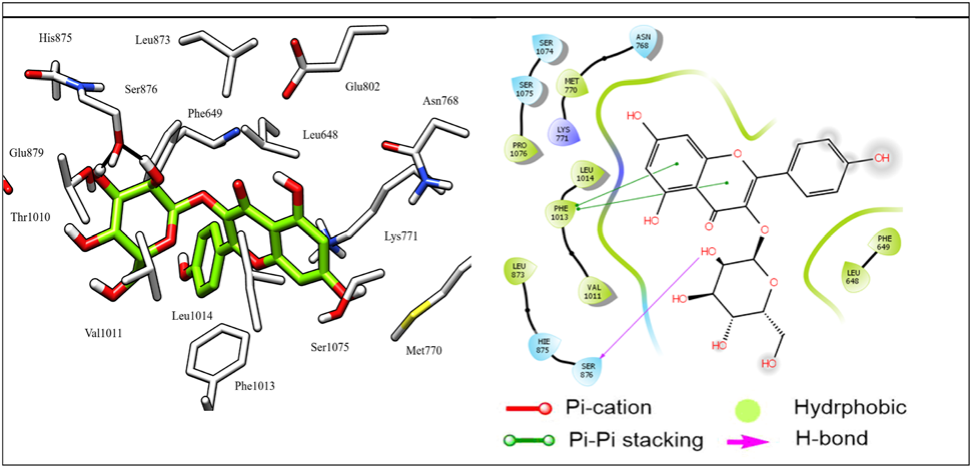
3D left and 2D right binding disposition of astragalin after docking calculations in the active site of XO enzyme. The amino acid residues were shown as stick model and H-bonds were shown as black lines.

Astragalin, which demonstrated the highest antioxidant activity, displayed excellent stability in the active site of XO, attributed to its significant hydrophobic interactions with the Pro1076, Leu873, Leu1014, Val1011, Leu648, Phe649, Met770, and His875 residues, which help to stabilize the molecule in its position. Additionally, it formed two pi–pi stacking interactions between its two aromatic rings and the key residue Phe1003 for XO (Fig. 8). These interactions further contribute to the stability of the molecule by enhancing its binding to the active site. Furthermore, two hydrogen bonds were formed between the hydroxyl groups of the glucose moiety and the Ser 876 residue.

Protocatechuic acid, *cis* and *trans*-tiliroside compounds are equally important as astragalin. These compounds demonstrate high stability in the enzyme's active site, as indicated by their docking score, which is higher than that of hypoxanthine. They establish crucial hydrogen bonds, electrostatic attraction forces, and hydrophobic interactions, highlighting their significance in XO enzyme inhibition.

**Fig. 9:**
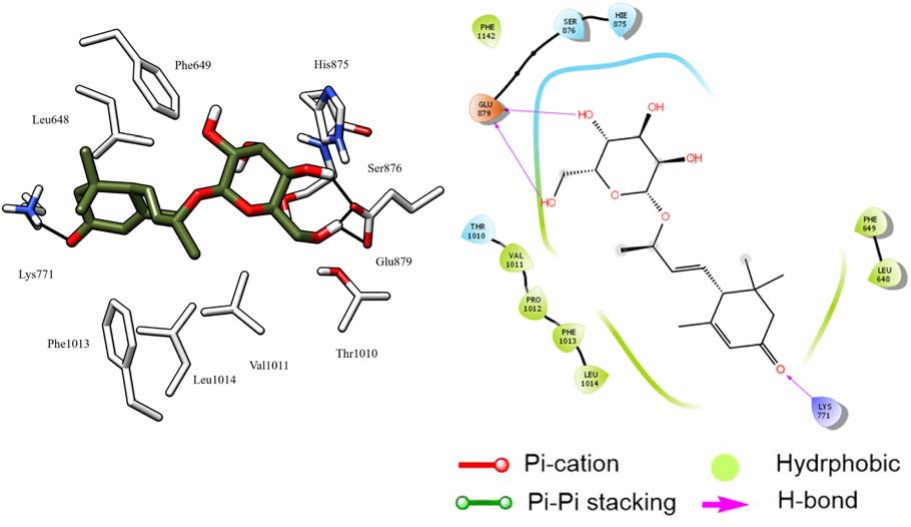
3D left and 2D right binding disposition of 3-oxo-α-ionol-β-d-glucopyranoside after docking calculations in the active site of XO enzyme. The amino acid residues were shown as stick model and H-bonds were shown as black lines.

Among all the compounds tested, only 3-oxo-α-ionol-β-d-glucopyranoside exhibited a docking score lower than hypoxanthine. This may be attributed to the absence of aromatic rings, which are essential for forming hydrophobic interactions, and Pi–Pi stacking with the residues involved in XO enzyme inhibition (Fig. 9).

#### Molecular dynamics simulation

The molecular dynamic simulations provide valuable insights into the dynamics and stability of the protein–ligand complex and can aid in the design of more effective and specific drugs. The process of conducting a molecular dynamics (MD) simulation of the complex structure containing the methyl-gallate ligand bound to the XO protein is given below.

The simulation was performed using the Desmond Academic Software-2022 and the OPLS_2005 force field, which is a commonly used force field for biomolecular simulations. The simulation was run on a Linux environment's Intel Xeon octal-core 2.88 Mhz.

To create the simulation system, the ligand–protein complex was solvated in an orthorhombic box with dimensions of 10.0 × 10.0 × 10.0 nm^3^. The box was filled with 23 328 TIP3P water molecules, which were used as the solvent for the simulation. To neutralize the net charge of the system, water molecules were replaced with Na^+^ ions.

To ensure that the protein remains in a stable conformation during the simulation, all protein atoms were maintained at a distance of 1.0 nm from the edges of the solvate box.

The simulation system was subjected to an energy minimization process of 50 000 steps, followed by equilibration for 100 ps at a temperature of 310 K. After equilibration, the system was subjected to a final production run of 100 ns MD simulation at 310 K under isothermal and isobaric conditions with a relaxation time of 0.2 ps.

Periodic boundary conditions were applied to the simulation to create an infinite number of identical simulation boxes. These conditions helped to simulate the behavior of the molecules in a larger system rather than in isolation.

Overall, the MD simulation was performed to study the behavior of the methyl-gallate-XO complex in a solvent environment and to gain insights into the interactions and dynamics of the system.^77^

The MD simulation was employed to confirm the previous mode of binding predicted by molecular docking for the methyl-gallate ligand to the XO protein.

The simulation was carried out to identify the regions of the protein influenced by the presence of the methyl-gallate ligand and to evaluate the stability of the system.^78^

To achieve this, the root mean square deviation (RMSD) values, the number of hydrogen bonds, the root mean square fluctuation (RMSF), and the protein secondary structure elements (SSE) were monitored throughout the simulation.

The results indicated that the methyl-gallate ligand remained in the same binding site both before and after the MD simulation and maintained hydrogen bonds with Glu879, Ser876, and Thr1010 residues, as well as hydrophobic interactions with Phe1009, Val1011, Leu1014, Pro1076, and Ala1078 residues.

In conclusion, the MD simulation validated the binding mode of the ligand to the protein and provided insight into the system's stability and dynamics.

### Root mean square deviation (RMSD)

**Fig. 10:**
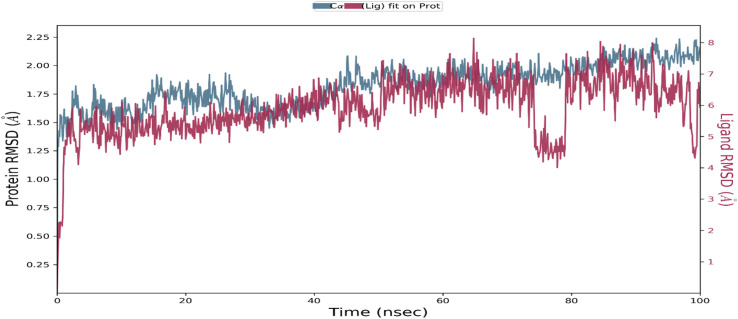
Protein–ligand Root Mean Square deviation (PL-RMSD) obtained from the molecular dynamic simulation trajectories.

The RMSD is a widely used measure to analyze structural changes in a protein–ligand complex during MD simulations. In this study, the protein and ligand PL-RMSD (Protein and Ligand Root Mean Square Deviation) were calculated from the MD simulation trajectories to measure the deviation between the initial and final configurations. A deviation of 1–3 Å is considered acceptable for small globular proteins. The PL-RMSD measures the distance between the protein and its ligand over the simulation time, and all protein frames (1000 frames) were aligned to the reference frame backbone (frame 0) before calculating the PL-RMSD based on the C-Alpha.^78^ Monitoring the RMSD of the protein provides insights into its structural conformations throughout the simulation, indicating the simulation's equilibration and its fluctuations around some thermal average structure. A high RMSD value implies an unstable protein system. In this study, the RMSD pattern comparison between the methyl-gallate bound to the XO protein showed comparable values from the first nanosecond of the MD simulation trajectory (Fig. 10). After 100 ns of MD simulation, the ligand-RMSD values were perfectly superimposed on the RMSD of the protein, indicating that the ligand was stable with respect to the protein and its binding pocket. The PL-RMSD values revealed that the XO protein remained periodically stable throughout the MD simulation while bound with the methyl-gallate ligand.

### Root mean square fluctuation (RMSF)

**Fig. 11:**
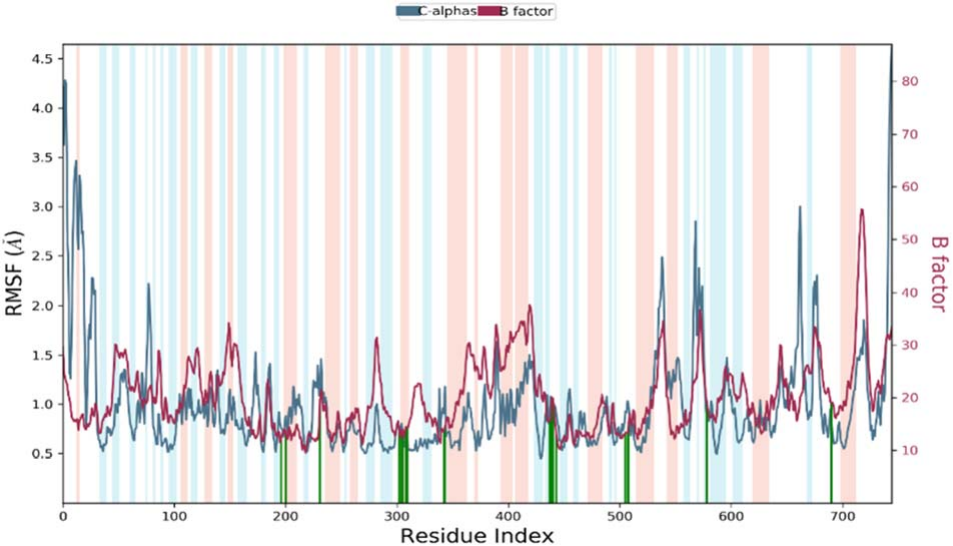
RMSF diagram of amino acids of the XO protein in the system studied methyl-gallate-XO enzyme. (Protein residues that interact with the ligand are marked with green-colored vertical bars Alpha-helical and beta-strand regions are highlighted in red and blue backgrounds, respectively, and the loop regions are highlighted in white backgrounds).

RMSF stands for root mean square fluctuation, which is a measure of the deviation of atom positions from their mean position over a period of time. In this case, RMSF monitoring is essential for characterizing local changes along the protein chain during the simulation.^79^Fig. 11 shows the RMSF diagram of the amino acids of the XO protein in the studied system, and the results were compared with the atomic displacement factor “*B* factor” of the Cα atoms of each protein residue. The “*B*-factor” is a measure of the degree of thermal motion of atoms in a crystal structure, and a higher “*B*-factor” value indicates that the atoms are more mobile or flexible. Peaks in the diagram indicate regions of the protein that fluctuate the most during the simulation. In this case, the N- and C-terminal regions fluctuate more than any other part of the protein, while alpha helices and beta strands are usually more rigid and thus fluctuate less than the loop regions. Overall, the comparison of the RMSF curve of the complex with the curve of the atomic displacement factor “*B*-factor” provides insights into the quality of the MD simulation. In this study, the RMSF monitoring has revealed that certain residues, including Leu577, Ala582, Ser587, Tyr592, Ile596, Tyr599, Phe649, Asp1110, Gly1139, Ser1234, and Pro1248, have higher RMSF values of alpha carbons (Cα). These residues with higher RMSF values may be more flexible or dynamic compared to other residues in the protein structure. This could suggest that they play an important role in the protein's function, such as binding to other molecules or undergoing conformational changes. In his simulation, the RMSF curve and the “*B*-factor” curve are parallel throughout the 100 ns molecular dynamic simulation, which suggests that the simulation has captured the expected atomic motions and dynamics and that the protein structure is behaving as expected. This is a good indication that the simulation has accurately modeled the protein structure and its dynamics.

### Protein–ligand contacts analysis

Protein–ligand interactions or 'contacts' are categorized into four types: hydrogen bonds, hydrophobic, ionic, and water bridges. Protein–ligand interactions during the 100 ns of MD simulation are presented in Fig. 11.

Hydrogen bonds: are one of the most important types of protein–ligand interactions and play a critical role in ligand binding.

The number and strength of hydrogen bonds between a ligand and its target protein can strongly influence the specificity, metabolization, and adsorption of the drug. Overall, the hydrogen bonding properties of a ligand and its interactions with the target protein should be considered in drug design, as they can strongly influence the drug's efficacy and specificity.^80^ In our study, it has been observed that the methyl-gallate ligand formed 3 hydrogen bonds on average during the 100 ns of MD simulation. These hydrogen bonds can be further categorized into four subtypes based on the acceptor or donor site: side-chain acceptor (Glu879 185.2%), side-chain donor (Ser876 6.5%), backbone acceptor (Ser876 3.5%), and backbone donor (Thr1010 2.3%).

These results support the findings of the molecular docking study, which predicted three hydrogen bonds formed between the methyl-gallate ligand and the residues of the active site of the protein.

The formation of hydrogen bonds between the ligand and the protein residues is a crucial aspect of ligand binding and is indicative of the stability of the protein–ligand complex.

Hydrophobic contacts: are another type of protein–ligand interaction that involves hydrophobic amino acids and an aromatic or aliphatic group on the ligand. In our study, the methyl-gallate ligand formed seven hydrophobic contacts on average during the 100 ns of molecular dynamic simulation, which can be further categorized into three subtypes: π-cation (Phe1009 19.10%), π–π stacking (Phe914 2.1%), and other non-specific interactions (Leu873, Phe914, Phe1009, Val1011, Leu1014, Pro1076, and Ala1078). These hydrophobic interactions can significantly contribute to the stability of the protein–ligand complex and can influence the binding affinity of the drug.

On the other hand, ionic interactions involve two oppositely charged atoms within 3.7 Å of each other and do not involve a hydrogen bond. The results obtained from our simulation study revealed that there were no ionic interactions between the methyl-gallate ligand and the protein.

Water bridges: are hydrogen-bonded protein–ligand interactions mediated by a water molecule. In our study, it has been observed that the methyl-gallate ligand formed multiple water bridges, especially between the Ser1008, Thr1010, Glu879, Arg880, and Ser876 amino acids and the ligand. These water bridges can contribute to the stability of the protein–ligand complex by mediating hydrogen bonds between the ligand and the protein residues.

**Fig. 12:**
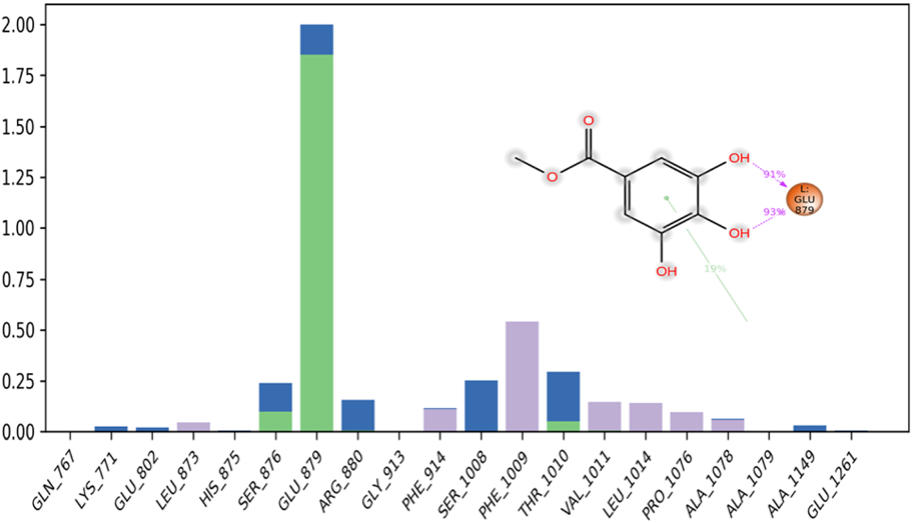
Protein–ligand contacts obtained from the MD simulation trajectories.

A timeline representation of the interactions and contacts (H-bonds, hydrophobic, ionic, and water bridges) can provide valuable insights into the dynamics and stability of the protein–ligand complex. The top panel of the Fig. 12 shows the total number of specific interactions that the protein makes with the methyl-gallate ligand over the 100 ns MD simulation trajectory.

**Fig. 13:**
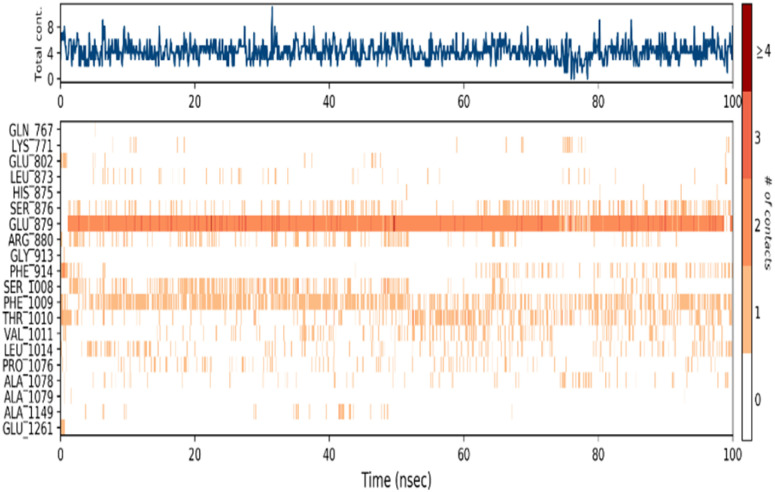
Timeline representation of the interactions and contacts (H-bonds, hydrophobic, ionic, and water bridges) obtained from the MD simulation trajectories.

This panel can help identify the most stable and persistent interactions between the protein and the ligand. The bottom panel of the figure shows which residues interact with the methyl-gallate ligand in each trajectory frame (Fig. 13). The darker orange shade indicates that some residues, specifically Thr1010, Ser1008, Arg880, Glu879, and Ser876, make more than one specific interaction with the ligand, which can contribute significantly to the stability of the protein–ligand complex. Overall, the timeline representation of the interactions and contacts help visualize the dynamics of the protein–ligand complex and provide valuable insights into the specific interactions that contribute to the stability of the complex.

### Protein secondary structure elements (SSE) analysis

**Fig. 14:**
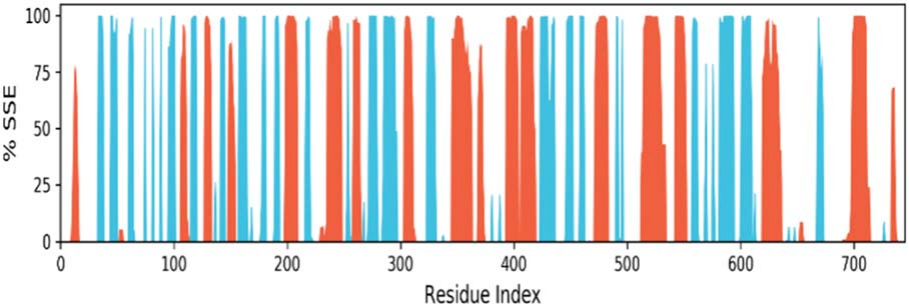
Protein secondary structure elements (SSE) monitored throughout the MD simulation. Alpha-helical 24.55%, beta-Strand 22.42%, total SSE 46.96%. (Alpha-helical and beta-strand regions are highlighted in red and blue backgrounds, respectively, and the loop regions are highlighted in white backgrounds).

The simulation analysis of the protein–ligand complex reveals that the secondary structure elements (SSE), including alpha-helices and beta-strands, remained stable throughout the simulation (Fig. 14). The conservation of these elements indicates the reliability and stability of the complex. The loop regions showed slight fluctuations, but there were no significant structural changes observed.

The ligand remained permanently attached to the active site of the protein without causing any modification, highlighting its stability. The analysis of the secondary structure elements suggested that the protein–ligand complex is structurally stable and specific, indicating that the ligand has a high affinity towards the protein and is likely to remain bound to it in its active form.

### Density functional theory (DFT) study

Density Functional Theory (DFT) is indeed one of the most widely used computational methods for studying the electronic structure of systems comprising a large number of electrons, such as molecules, solids, surfaces, and nanostructures. DFT has proven to be very successful in predicting a wide range of properties, including electronic structure, energetics, reaction mechanisms, and spectroscopic properties. Its versatility and computational efficiency make it an indispensable tool in theoretical chemistry, physics, and materials science.^81,82^

### Geometry optimization

**Fig. 15:**
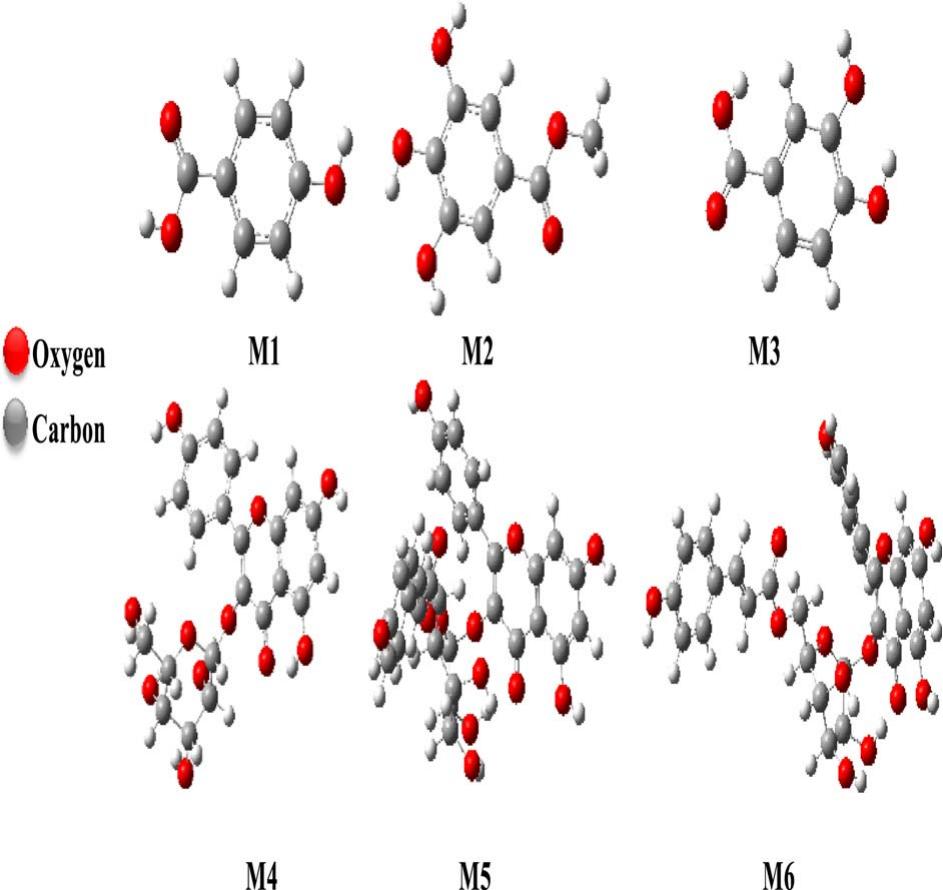
Optimized structure of titled compounds obtained at B3LYP/6-31G (d, p) level in gas phase.

The optimization of different structures was performed by Gaussian 09 at the B3LYP/6-31G (d, p) level. Fig. 15 shows the structure of our compounds obtained after the geometry optimization in the gas phase.

### Analysis of molecular orbital and the global reactivity index

Understanding the energy gap is essential in fields such as organic electronics, photovoltaics, and molecular design, where it influences the conductivity, reactivity, and optical properties of materials.

Experimental and theoretical techniques are often employed to measure or calculate the energy gap of molecules, providing valuable insights into their electronic structure and potential applications. The energy gap, often referred to as the HOMO–LUMO gap, is a crucial concept in the field of molecular electronic structure. It represents the energy difference between the Highest Occupied Molecular Orbital (HOMO) and the Lowest Unoccupied Molecular Orbital (LUMO) in a molecule. This energy gap plays a significant role in determining the various properties and behaviors of molecules. Therefore, Table 6 provides details on the energy levels (HOMO, LUMO), the energy gap, total energy, dipolar moment (*μ* total), and linear polarizability (*α*_ToT_) for the discussed molecules computed by DFT method and B3LYP with 6-31G (d, p) level in gas phase (1–6).

**Table 6:** The calculated descriptors of studied compounds 1–6 obtained at B3LYP/6-31G (d, p) level in gas phase

Descriptors molecule	Gas phase					
	1	2	3	4	5	6
*E*_HOMO_ (eV)	−6.41	−6.22	−6.24	−6.27	−5.63	−5.63
*E*_LUMO_ (eV)	−1.04	−1.43	−1.14	−2.24	−1.53	−1.53
|Δ*e*_gap_| (eV)	5.36	4.78	5.09	4.02	4.09	4.09
*E* (u. a)	−496.05	−685.8	−571.2	−1639.8	−2136.7	−2136.7
*μ* total (D)	1.85	1.30	5.57	2.38	3.07	3.07
*α*_ToT_ (Bhor^3^)	79.73	177.46	83.25	387.23	310.96	310.96

On the other hand, the global indices of reactivity are a set of parameters used in theoretical chemistry to characterize the overall reactivity of a molecule or system. These indices provide valuable insights into the chemical behavior and stability of molecules.

**Fig. 16:**
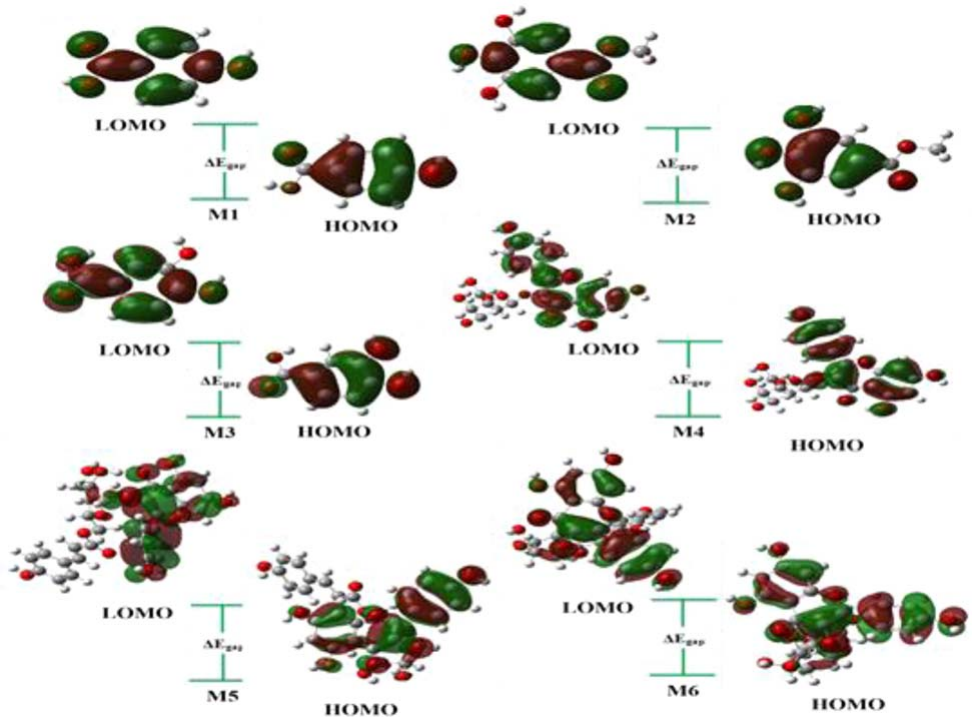
3D representation of HOMO, LUMO of the studied molecules (1–6).

As indicated in Table 6, the energy gap among the studied compounds follows a decreasing order: 4 < 5 < 6 < 2 < 3 < 1. Compound M4 exhibits the smallest gap, measured at 4.02 eV, along with the highest values of linear polarizability (387.23 Bohr^3^). Consequently, it is inferred that 4 is less stable and demonstrates higher lipophilicity and reactivity compared to the other compounds. Though the highest values of gap are for compound 1, with 5.36 eV and small linear polarizability (79.73 Bohr^3^). The 3D visualization of the frontier molecular orbitals, including the Lowest Unoccupied Molecular Orbital (LUMO) and Highest Occupied Molecular Orbital (HOMO) computed in the gas phase, is depicted in Fig. 16.

On the other hand, the global indices of reactivity are a set of parameters used in theoretical chemistry to characterize the overall reactivity of a molecule or system. These indices provide valuable insights into the chemical behavior and stability of molecules. Some commonly used global indices of reactivity are calculated in this paper including chemical potential (*μ*), chemical hardness (*η*), electronegativity (*χ*), electrophilicity (*ω*), and nucleophilicity (*N*). Based on the results outlined in Table 7, it was observed that compound 1 exhibited a higher ionization energy (IP = 6.41 eV) and a small value of electron affinity (EA = 1.04 eV). This suggests a larger tendency for the atom to accept an additional electron, indicating that M1 is highly reactive. Also, this molecule is less likely to act as an electron acceptor because it shows a minor electronegativity value *x* = 3.72 and a lower electrophilicity of *ω* = 2.58.

**Table 7:** Obtained values of global reactivity descriptors for studied compounds (1–6) employing DFT with B3LYP/6-31G (d, p) at the gas phase

Gas phase								
Molecule	*I*	*A*	*μ*	*X*	*η*	*S*	*ω*	*N*
1	6.41	1.04	−3.72	3.72	2.68	0.18	2.58	2.95
2	6.22	1.43	−3.82	3.82	2.39	0.20	3.05	3.14
3	6.24	1.24	−3.74	3.74	2.50	0.20	2.79	3.12
4	6.27	2.24	−4.25	4.25	2.01	0.24	4.49	3.09
5	5.63	1.53	−3.58	3.58	2.05	0.25	3.12	3.73
6	5.63	1.53	−3.58	3.58	2.05	0.25	3.12	3.73

Furthermore, compounds 2 and 4 displayed the minimum values of chemical potential, measured at *μ* = −3.82 and −4.25, respectively. This observation proposes that these molecules could be characterized as strong electron-acceptor reagents. Conversely, compounds 5 and 6 exhibited higher values of chemical potential (*μ* = −3.58) and a greater nucleophilicity index (*N* = 3.73), indicating their potential as strong electron-donor molecules.

Softness is particularly useful in predicting and understanding chemical reactions, especially those involving electron transfer or polarization effects. As result, the highest value of softness was seen with 5 and 6 with *S* = 0.25, which signifies a molecule that is highly reactive and prone to donate electrons, making it potentially important in various chemical reactions and interactions.

### Molecular electrostatic potential (MEP)

Molecular Electrostatic Potential (MEP) is a valuable tool in computational chemistry used to visualize and analyze the distribution of electron density in a molecule.

It provides insights into the reactivity, polarity, and intermolecular interactions within the molecule.

**Fig. 17:**
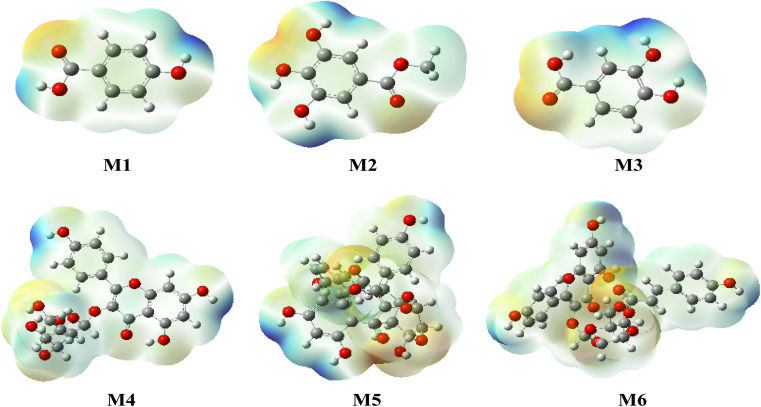
MEP shaped by mapping of total density over electrostatic potential in gas phase for the studied compounds (1–6).

MEP is derived from quantum chemical calculations, such as Density Functional Theory (DFT) or Hartree-Fock methods, and is typically represented as a 3D surface plot. In this computational study, we analyzed the molecular electrostatic potential (MEP) of the titled molecules (1–6) using the Density Functional Theory (DFT) method with the B3LYP/6-31G (d, p) basis set for the optimized geometries. The results of the MEP for the investigated molecules are illustrated in Fig. 17. These positive regions are associated with nucleophilic reactivity, where molecules or molecular sites are more likely to donate electrons and behave as nucleophiles in chemical reactions. Consequently, the negative regions are typically represented by red and yellow colors, indicating areas of high electron density. These negative regions are associated with electrophilic reactivity, where molecules or molecular sites are more likely to accept electrons and behave as electrophiles in chemical reactions. On the contrary, the positive regions in blue color represent areas of low electron density. These positive regions are associated with nucleophilic reactivity, where molecules or molecular sites are more likely to donate electrons and behave as nucleophiles in chemical reactions.

### Ligand based druglikeness proprieties

As we can observe from the results of Table 8, compounds 1, 2, 3, 4, 7 and TROLOX reference follow the Lipinski rule, which specifies a molecular weight of less than 500 g mol^−1^ with *M* = 138.12, 184.15, 154.12, 462.40, 370.44, and 250.29 g mol^−1^ respectively, except compounds 5 and 6 more than 500 g mol^−1^, which have a number of hydrogen bond acceptors (HBA = 3–7), with the exception of compounds 4, 5 and 6, with HBA = 11–13 violating one of Lipinski's rules. Also, all studied compounds have a number of hydrogen bond donors (HBD = 2–7) and lipophilicities less than 5, with values between 0.57 and 2.47, which is in agreement with Lipinski's rules. Compound 4 exhibits lipophilicity less than zero, which is not in violation of Lipinski's rules.

**Table 8:** Drug-likeness results of compounds

Drug likeness properties	TROLOX	1	2	3	4	5	6	7
Molecular weight g mol^−1^	250.29	138.12	184.15	154.12	462.40	594.52	594.52	370.44
Consensus log *P* o/w	2.47	1.05	0.57	0.65	−0.09	1.52	1.21	0.66
Log *S*	−3.36	−2.07	−1.73	−1.86	−3.13	−4.93	−4.93	−1.83
NHBA	4	3	5	4	11	13	13	7
NHBD	2	2	3	3	7	7	7	4
Molar refractivity	68.94	35.42	43.79	37.45	112.94	149.51	149.51	95.03
Lipinski	Yes	Yes	Yes	Yes	No	No	No	Yes
Ghose	Yes	No	Yes	No	Yes	No	No	Yes
Veber	Yes	Yes	Yes	Yes	No	No	No	Yes
Egan	Yes	Yes	Yes	Yes	No	No	No	Yes
Muegge	Yes	No	No	No	No	No	No	Yes
Bioavailability score	0.85	0.85	0.55	0.56	0.17	0.17	0.17	0.55
Synthetic accessibility (SA)	3.05	1	1.50	1.07	5.33	5.96	5.96	5.65
TPSA (A)° 2	66.76	57.53	86.99	77.76	190.28	216.58	216.58	116.45
No. of rotatable bonds	1	1	2	1	5	8	8	5
DLS	0.59	−0.37	−0.65	0.23	0.72	0.75	0.75	0.52

Indeed, the determination of the number of rotatable bonds for the developed compounds revealed values less than nine (RBN = 1–8), suggesting that these compounds are likely to have good oral bioavailability.

In addition, derivatives 2, 3 and 7 presented good solubility with log *S* = −1.73 and −1.83, −1.86, respectively. Furthermore, TROLOX and 1, 4, 5, 6 show modest solubility in the range of −2.07 to −4.93.

An ideal molar refractivity value falls within the range of 40 to 130 for optimal absorption and oral bioavailability.^47^ The molar refractivity values of the designed compounds 2, 4, 7 and the TROLOX reference range from 43.79 to 95.03 m^3^ mol^−1^. This indicates that the proposed compounds exhibit favorable intestinal absorption and oral bioavailability.

Another reliable indicator to consider for drug absorption in the intestine and blood–brain barrier penetration is the topological polar surface area (TPSA). The compounds TROLOX, 3, 2 and 7 demonstrate good intestinal absorption, with TPSA values arranged in increasing order of 66.75 < 77.76 < 86.99 < 116.45, respectively. Though compound 1 exhibits satisfactory blood–brain barrier penetration with a TPSA value less than 60 Å^2^ (TPSA = 57.53 Å^2^).

On the other hand, the drug-likeness model score evaluates how closely a compound resembles known drugs based on its physicochemical properties and molecular structures. A high drug likeness model score indicates that a molecule possesses more favorable pharmacokinetic and pharmacodynamic properties, thereby enhancing its potential as a drug candidate.^83^ As per the predictions, the drug likeness model score for the majority of the compounds ranged from 0.23 to 0.75. It's noteworthy that compounds 1 and 2 deviated from this trend, exhibiting values less than zero.

Further, Synthetic Accessibility (SA) scores are used to assess the ease with which a molecule can be synthesized. Low SA scores may generally suggest a more feasible synthesis route and fewer complex molecules; the SA score goes from 1 to 10.^84^ The studied compounds have a SA score between 1 and 5.95.

**Fig. 18:**
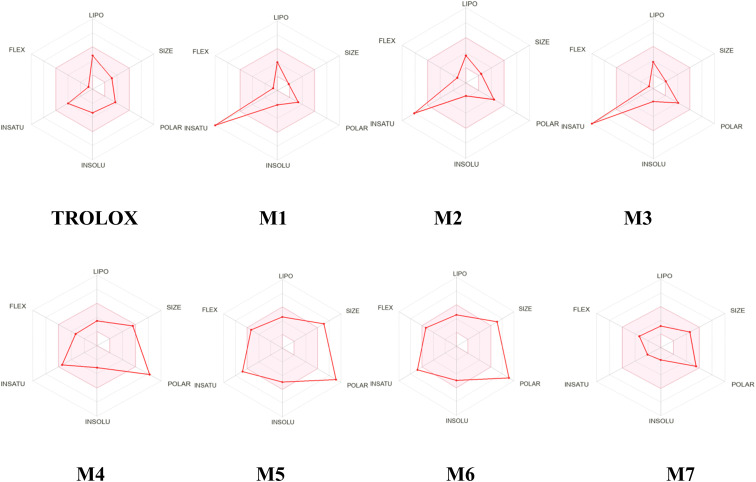
Oral bioavailability radar of studied compounds (1–7) and TROLOX.

An analysis of the bioavailability of RADAR (Fig. 18) provides information on the bioavailability properties of the selected compounds (1–7) and TROLOX. The pink area in the RADAR represents the most favorable zone for each bioavailability property. As observed in Table 9, all the studied compounds adhere to the recommended 500 g mol^−1^ size limit by Lipinski for good drug candidates, in contrast to the 594.52 g mol^−1^ obtained for M5 and M6, respectively. Polarity (POLAR) was assessed using Total Polarity Surface Area (TPSA) between 57.53 and 116.45 Å^2^. The flexibility (FLEX) property was evaluated using the number of rotatable bonds, whose value should not exceed nine. Evidently, all the selected compounds and TROLOX are within the recommended range. Lipophilicity (LIPO) and insolubility (INSOLU) were assessed using *x* log *P*_3_ and ESOL (log *S*), with recommended ranges of −0.7 to +5.0 and 0 to 6, respectively. Importantly, all compounds except Lupeol fall within the recommended values of *x* log *P*_3_, and they have values of ESOL (log *S*) less than 0.

**Table 9:** The ADME/T test result of ligands (various pharmacokinetics properties)

Class	Proprieties	Trolox	1	2	3	4	5	6	7
Absorption	Caco-2 permeability	1.038	1.13	0.925	0.049	0.285	0.262	0.262	−0.067
	Pgp-inhibitor	No	No	No	No	No	Yes	Yes	No
	Pgp-substrate	No	No	No	No	Yes	Yes	Yes	Yes
	Skin permeability	−2.727	−2.392	−2.777	−2.696	−2.735	−2.735	−2.735	−3.316
	Human intestinal absorption (HIA)	93.717	74.377	61.796	71.372	40.237	56.413	56.413	40.548
Distribution	BBB permeant	0.165	−0.331	−1.03	−0.797	−1.703	−1.789	−1.789	−0.96
	VDss	−0.942	−0.584	−0.143	−0.468	−0.17	−0.05	−0.05	−0.387
Metabolism	CYP1A2 inhibitor	No	No	No	No	No	No	No	No
	CYP2C19 inhibitor	No	No	No	No	No	No	No	No
	CYP2C9 inhibitor	No	No	No	No	No	No	No	No
	CYP2D6 inhibitor	No	No	No	No	No	No	No	No
	CYP3A4 inhibitor	Yes	No	No	Yes	Yes	No	No	No
Toxicity	hERG (hERG blockers)	No	No	No	No	No	No	No	No
	Maximum tolerated dose	1.398	1.002	0.696	0.948	0.921	0.479	0.479	0.643
	Acute oral toxicity	2.398	1.862	2.009	1.876	2.754	2.614	2.614	1.98

### Pharmacokinetics and target prediction

*In silico* screening software for pharmacokinetics encompasses a range of tools designed to predict the various pharmacokinetic properties of potential drug candidates. These tools utilize computational models and algorithms to estimate parameters such as absorption, distribution, metabolism, excretion, and toxicity (ADME/T). In this Azt, we have explored the prediction of pharmacokinetics properties of our compound's (1–7) and TROLOX references.

In absorption, studied compounds 1, 2, and TROLOX have a Caco_2_ permeability greater than 0.9 with Papp = 1.13, 0.925, and 1.038 log Papp in 10^−6^ cm s^−1^ respectively. The human intestinal absorption (% absorbed) values for all the compounds exceed 30%. When reviewing our P-glycoprotein data, we verified that compounds exhibit high levels of cellular absorption.

Further, to estimate the ability of a substance to penetrate the skin barrier, we predict the skin permeability (SP) of all compounds. High skin permeability values suggest that a compound can readily pass through the skin barrier, while low values indicate limited penetration.^85^ This information is valuable in drug development and formulation, as well as in the assessment of potential risks associated with dermal exposure to chemicals. Consequently, as shown in the table, all examined compounds have a higher skin permeability (SP) rate.

In the distribution phase, the Blood–Brain Barrier (BBB) is a critical consideration. We reported that all compounds (1–7) and TROLOX are poorly distributed to the brain with log BB < −1. Also, the reported values for Volume of Distribution at steady state (VDss) are consistently higher than −0.15 and less than 0.45 for all compounds, except for M5 and M6, which fall below −0.15.

On the other hand, based on the results from the metabolism section, compounds (1–7) and TROLOX are identified as no potential inhibitors of cytochrome P450 1A2, P450 2C19, and P450 2C9.

In the toxicity section, all the compounds demonstrated no inhibition of hERG I and II proteins. The results also suggested that they have the highest value for Oral Rat Acute Toxicity (LD_50_) (mol kg^−1^).

### Target prediction

Overall, target identification and validation are integral parts of the drug discovery process, contributing to the development of safe and effective therapies and elucidating their mechanism of action. Advances in computational methods, high-throughput screening technologies, and molecular biology have greatly facilitated target discovery and characterization in recent years, accelerating the drug development pipeline. Molinspiration is indeed a software platform used for various computational chemistry tasks, including the prediction of bioactivity scores.

In this paper, we employed this software to identify probable bioactivity scores (Table 10) for the studied compounds (1–7) and reference ligands. The analysis suggests that the main mode of interaction for examined compounds 4 and 7 is likely to act as enzyme inhibitors and portease inhibitor for 7; while the other compounds don't exhibit any measurable bioactivity scores given that their bioactivity scores are less than zero.

**Table 10:** Target and bioactivity scores prediction of studied compounds (1–7)

Property	1	2	3	4	5	6	7
GPCR ligand	−0.98	−0.89	−0.88	0.06	−0.10	−0.10	0.11
Ion channel modulator	−0.39	−0.36	−0.35	−0.05	−0.60	−0.60	0.02
Kinase inhibitor	−1.21	−0.89	−1.10	0.10	−0.24	−0.24	−0.27
Nuclear receptor ligand	−0.62	−0.72	−0.58	0.20	−0.07	−0.07	0.16
Protease inhibitor	−1.19	−1.03	−1.09	−0.05	−0.09	−0.09	**0.26**
Enzyme inhibitor	−0.41	−0.36	−0.34	**0.41**	0.05	0.05	**0.55**

## Conclusions

In this study, we aimed to evaluate the *in vitro* antioxidant activity, conduct an in *silico* study, and identify the chemical constituents of *Helianthemum confertum*, which have not been previously investigated.

The ethyl acetate extract's phytochemical analysis revealed a high total phenolic content. Seven compounds were isolated and identified: *para*-hydroxybenzoic acid 1, methyl gallate 2, protocatechuic acid 3, astragalin 4, *trans*-tiliroside 5, *cis*-tiliroside 6, and 3-oxo-α-ionol-β-d-glucopyranoside 7. Compounds 2 and 7 were novel discoveries for the genus *Helianthemum*.

The results showed that the compounds and extracts from this species have strong antioxidant properties, which is in line with previous research showing that the amount of free radicals a compound can scavenge is dependent on its molecular structure and the location and number of hydroxyl groups.

In light of the several biological effects of *tran*s-tiliroside and the possibility of methyl gallate as an adjuvant for anticancer treatment, as well as the large quantities of these compounds that were isolated and purified in this study, additional research is required. The fact that this *Helianthemum* species has such a high concentration of these bioactive components makes it interesting for potential industrial use. By highlighting the significance of phytochemical identification and thorough pharmacological evaluations, our research highlights the promise of developing drugs from plants for future medical therapies other than cancer.

## Data availability

All data generated or analyzed during this study are included in this published article and its ESI† files.

## Author contributions

Y. C.: performed the experiment, wrote the paper; S. B.: chose and collected the plant material; A. B.; N. A.: Performance of the docking study; E. M.: contributed with reagents, materials, and analysis tools; O. S.; Z. B.: follow the calculation of MD simulation; H. B.: DFT and ADME/T studies; Y. C., S. B., F. B., M. Z.: analyzed the data; F. B.: responsible for the principal idea, structural analysis, work supervision, and manuscript writing.

## Conflicts of interest

The authors declare no conflict of interest.

## Supplementary Material

RA-014-D4RA02540G-s001
