# Supplementary material for: Phytochemical on-line screening and in silico study of Helianthemum confertum: antioxidant activity, DFT, MD simulation, ADME/T analysis, and xanthine oxidase binding
Source: RSC Adv. 2024 Jul 15;14(31):22209–28. doi: 10.1039/d4ra02540g (PMC11247359; doi:10.1039/d4ra02540g)
Supplement: RA-014-D4RA02540G-s001 [file RA-014-D4RA02540G-s001.pdf]

Electronic Supplementary Material (ESI) for RSC Advances.
This journal is © The Royal Society of Chemistry 2024

Phytochemical on-line screening and *in silico* study of *Helianthemum confertum*: antioxidant activity, DFT, MD simulation, ADME/T analysis and xanthine oxidase binding

Yasmine Chemam[a,b,c]*, Samir Benayache[a], Abdeslem Bouzina[c], Eric Marchioni[b] , Omar sekiou[d], Houria Bentoumi[c], , Minjie Zhao[b], Zihad Bouslama[d], Nour-Eddine Aouf[c] and Fadila Benayache[a]

[a]Unité de Recherche Valorisation des Ressources Naturelles, Molécules Bioactives et Analyses Physicochimiques et Biologiques, Université des Frères Mentouri, Constantine 1, Route d'Aïn El Bey, 25000 Constantine, Algeria.
[b]Chimie Analytique des Molécules Bioactives, Institut Pluridisciplinaire Hubert Curien (UMR 7178 CNRS/UDS), 74 route du Rhin, 67400 Illkirch, France.
[c]Laboratory of Applied Organic Chemistry, Bioorganic Chemistry Group, Department of Chemistry, Sciences Faculty, Badji Mokhtar Annaba University, Box 12, 23000 Annaba, Algeria
[d]Environmental Research Center, Alzon Castle, Boughazi Said Street, PB 2024, Annaba 23000, Algeria.

Molecule 1: Para-hydroxybenzoic acid

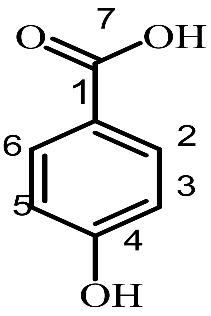

1

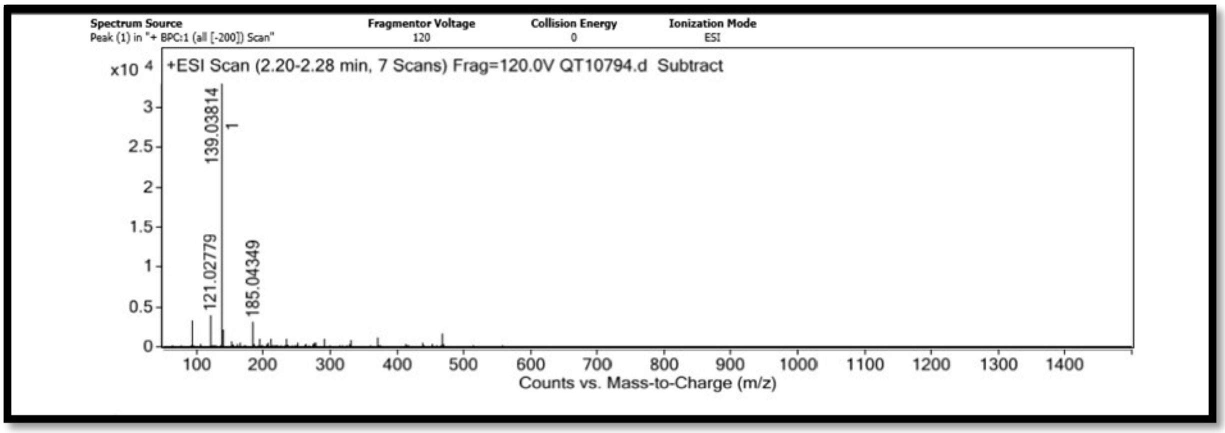

Figure.1.HRESI-MS (+) of para-hydroxybenzoic acid

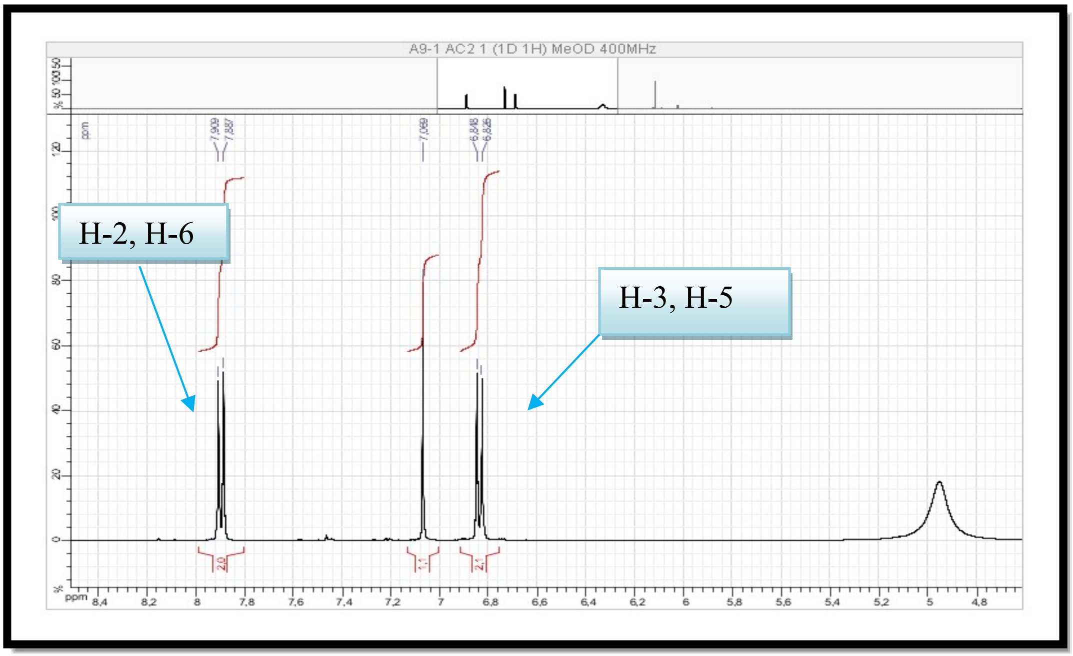

Figure.2. ^{1}H NMR spectrum (400MHz, CD_3OD, δppm) of para-hydroxybenzoic acid

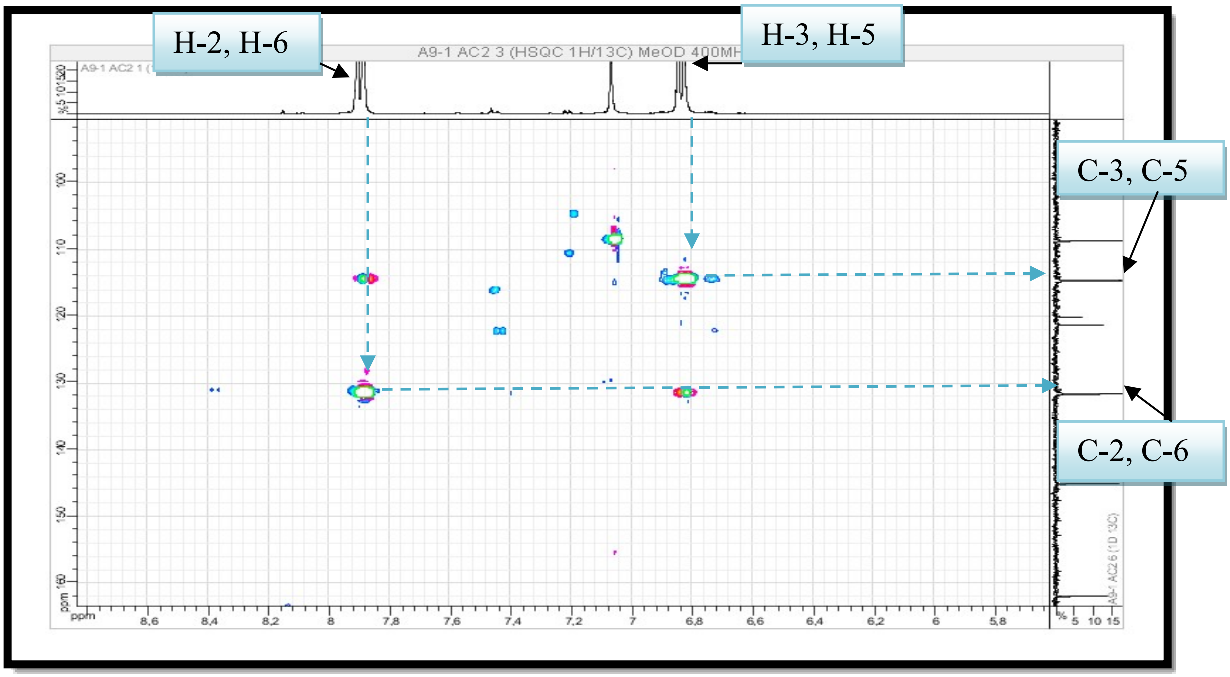

Figure .3.HSQC spectrum (400MHz, CD_3OD, δppm) of para-hydroxybenzoic acid

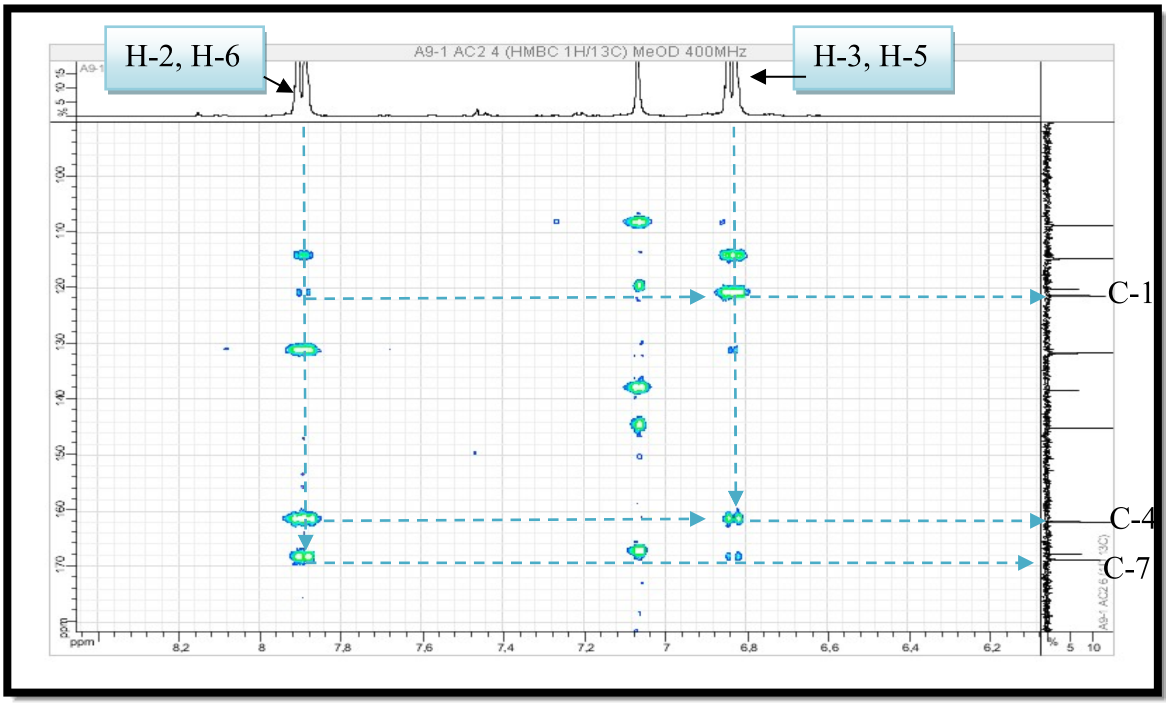

Figure .4.HMBC spectrum (400MHz, CD_3OD, δppm) of para-hydroxybenzoic acid

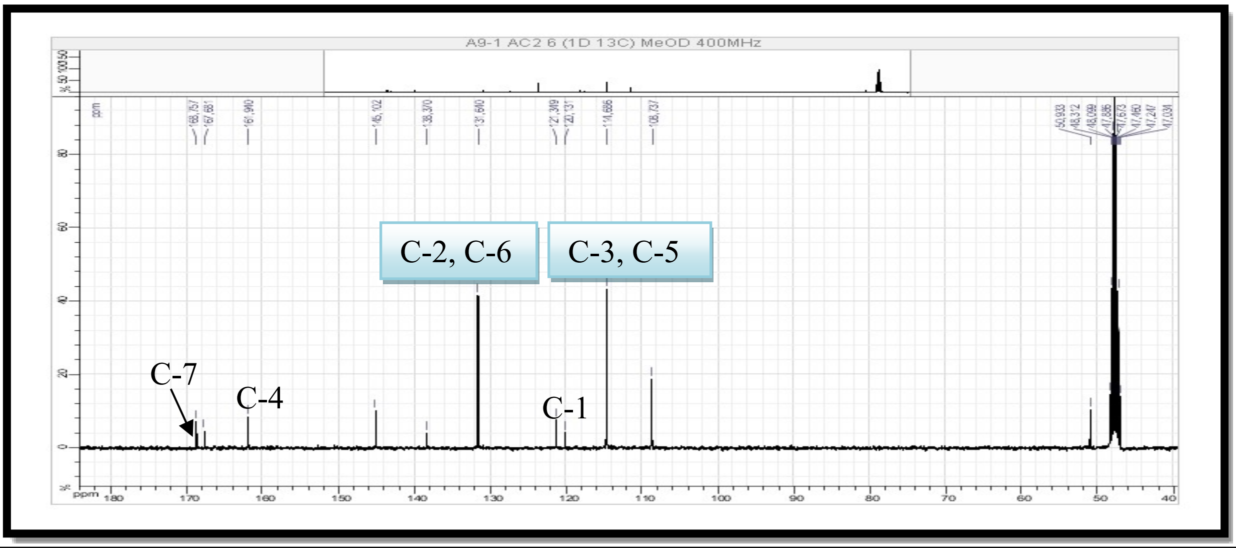

Figure.5. ^{13}C NMR spectrum (100MHz, CD_3OD, δppm) of para-hydroxybenzoic acid

Figure.5. ^{13}C NMR spectrum (100MHz, CD_3OD, δppm) of protocatechuic acid

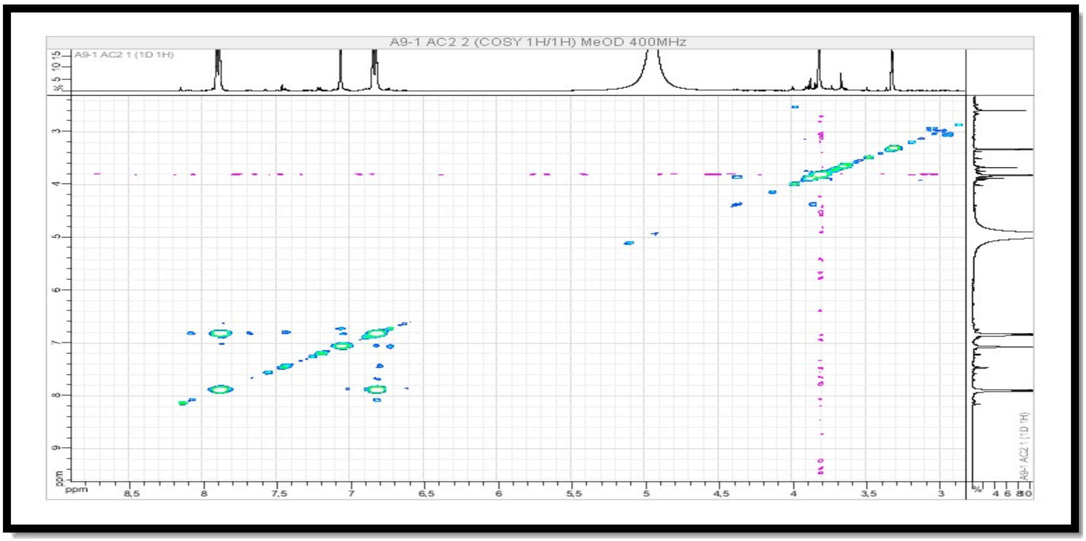

Figure.6. COSY spectrum (400MHz, CD_3OD, δppm) of para-hydroxybenzoic acid

Molecule 2: Methyl gallate

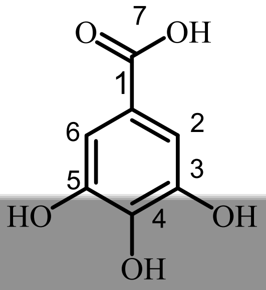

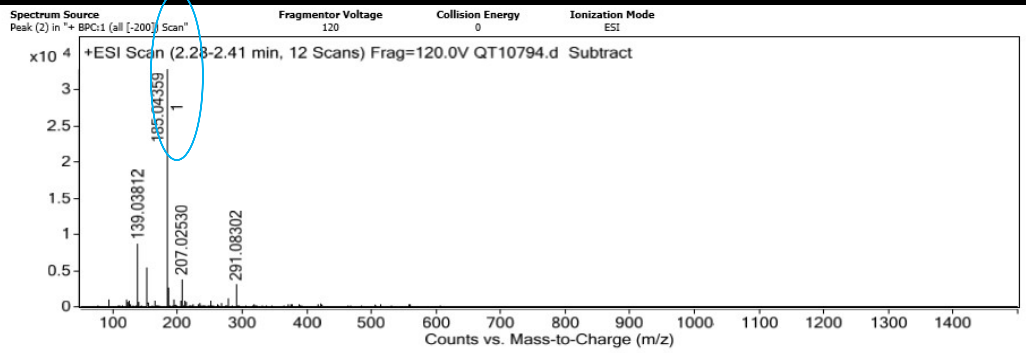

Peak List

m/z	z	Abund	Formula	Ion
139.03812		8788.2		
153.01769		5515.2		
185.04359	1	33319.5		
186.04704	1	2621.1		
207.0253		3862.7		
291.08302		3094.2		

Formula Calculator Results

Formula	Best	Mass	Tgt Mass	Diff (ppm)	Mz	Ion Species	Score
C8 H8 O5		184.03632	184.03717	4.66	185.04359	C8 H9 O5	83.56

Figure.7.HRESI-MS (+) of methyl gallate

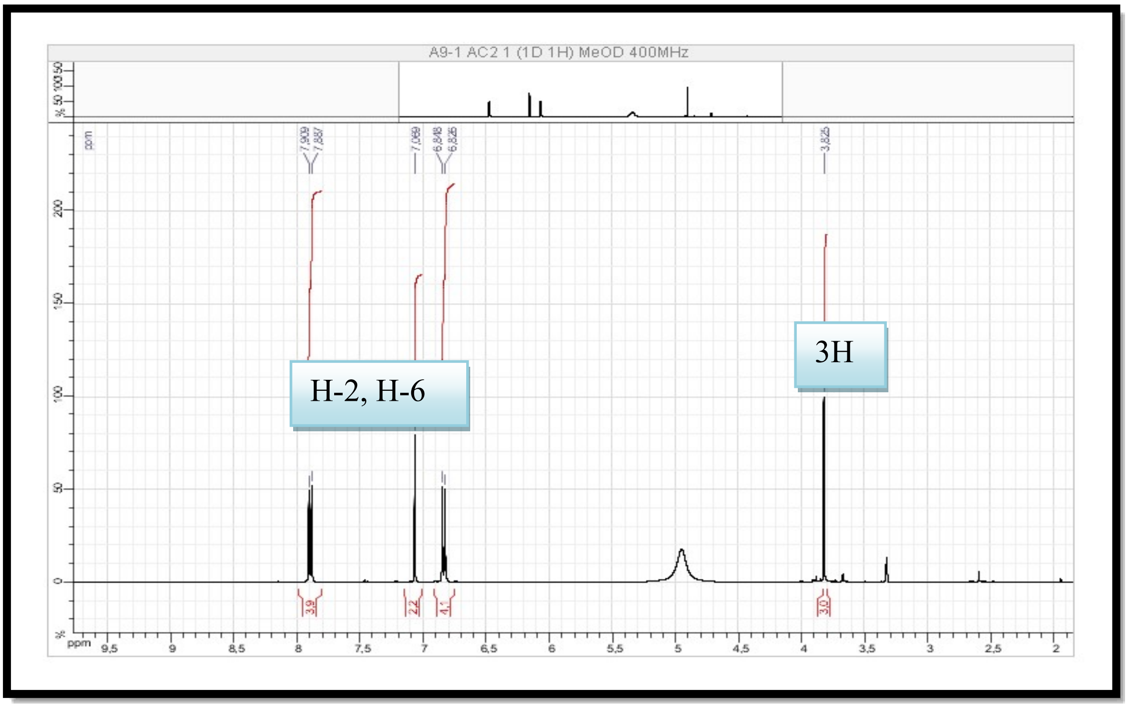

Figure.8. ^{1}H NMR spectrum (spreading out 2) (400MHz, CD_3OD, δppm) of methyl gallate

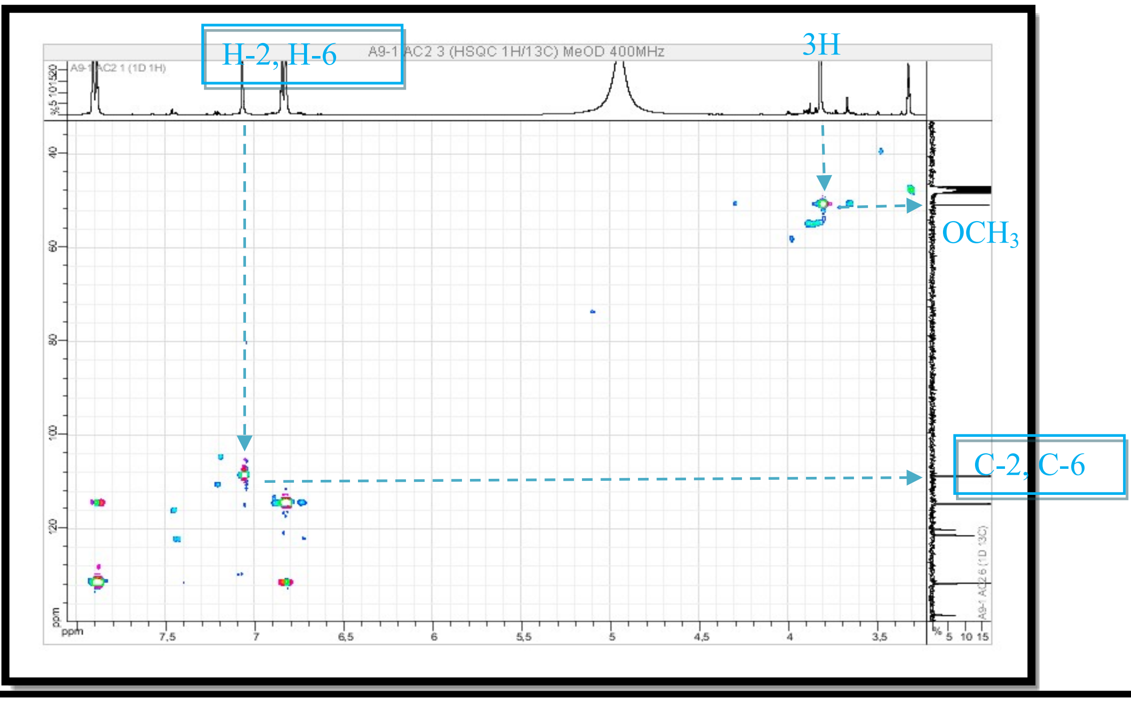

Figure .9.HSQC spectrum (spreading out 2) (400MHz, CD_3OD, δppm) of methyl gallate

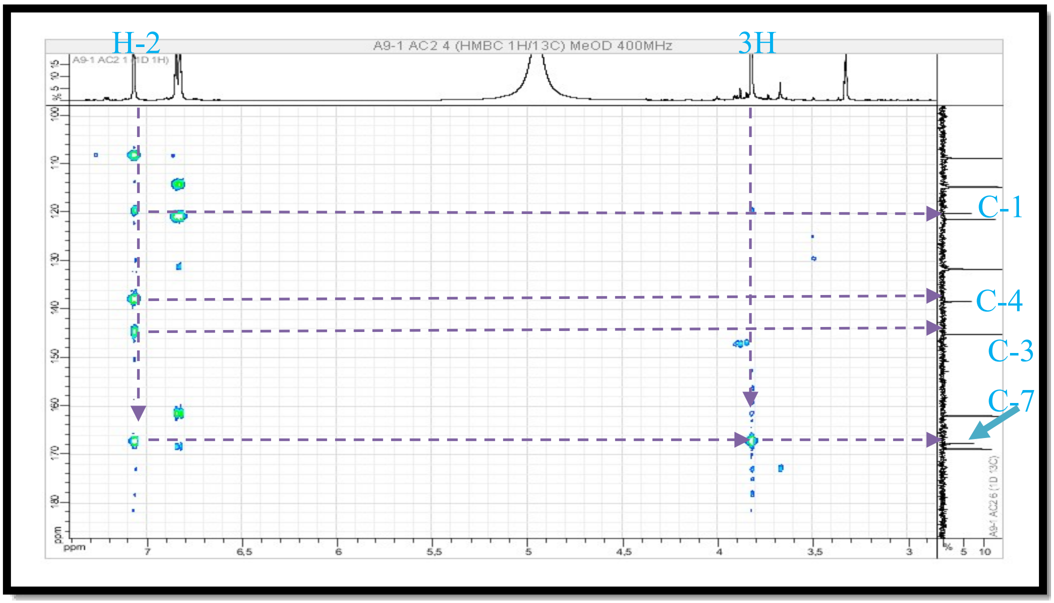

Figure .10.HMBC spectrum (spreading out 2) (400MHz, CD_3OD, δppm) of methyl

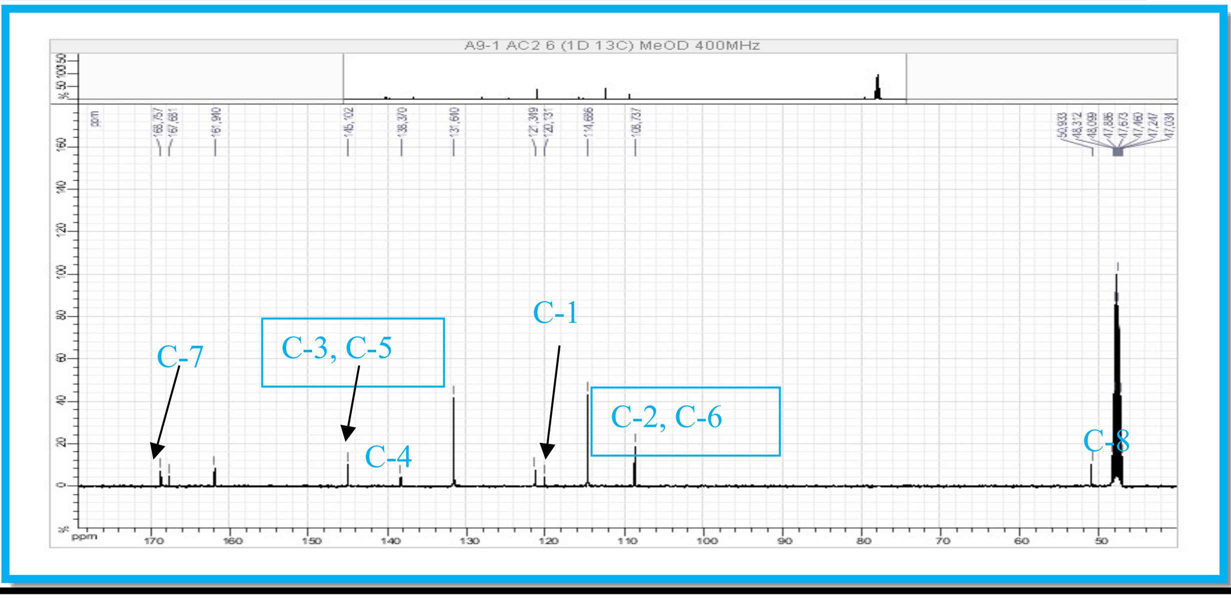

Figure.11. ^{13}C NMR spectrum (spreading out 2) (100MHz, CD_3OD, δppm) of methyl gallate

Molecule 3: Protocatechuic acid

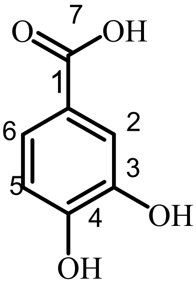

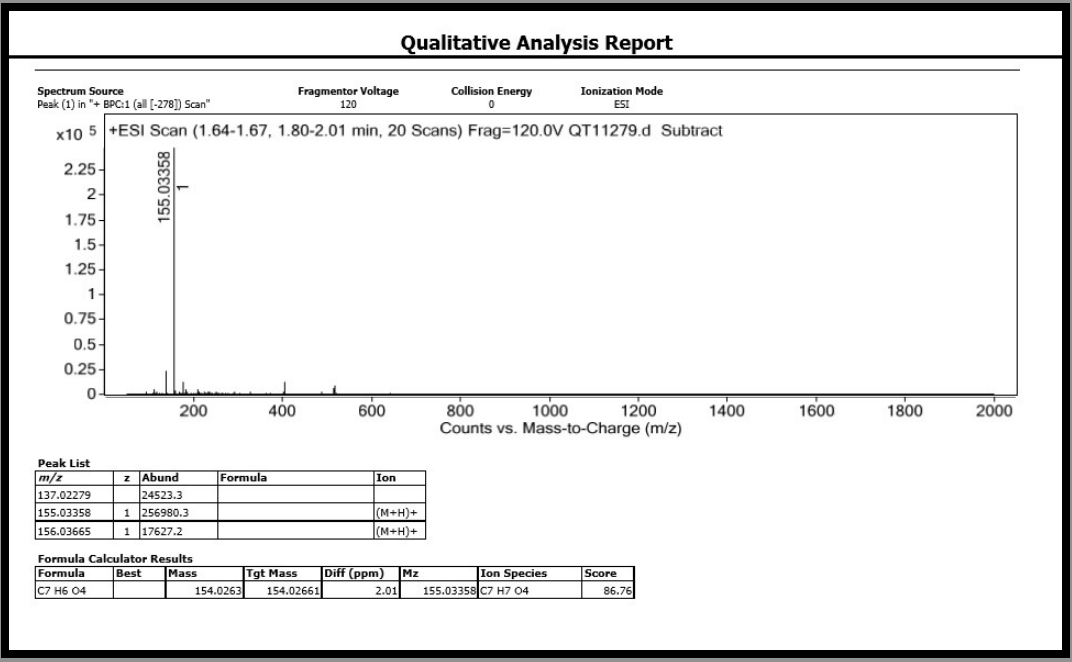

Peak List

m/z	z	Abund	Formula	Ion
137.02279		24523.3		
155.03358	1	256980.3		(M+H)+
156.03665	1	17627.2		(M+H)+

Formula Calculator Results

Formula	Best	Mass	Tgt Mass	Diff (ppm)	Mz	Ion Species	Score
C7 H6 O4		154.0263	154.02661	2.01	155.03358	C7 H7 O4	86.76

Figure.12.HRESI-MS (+) of Protocatechuic acid

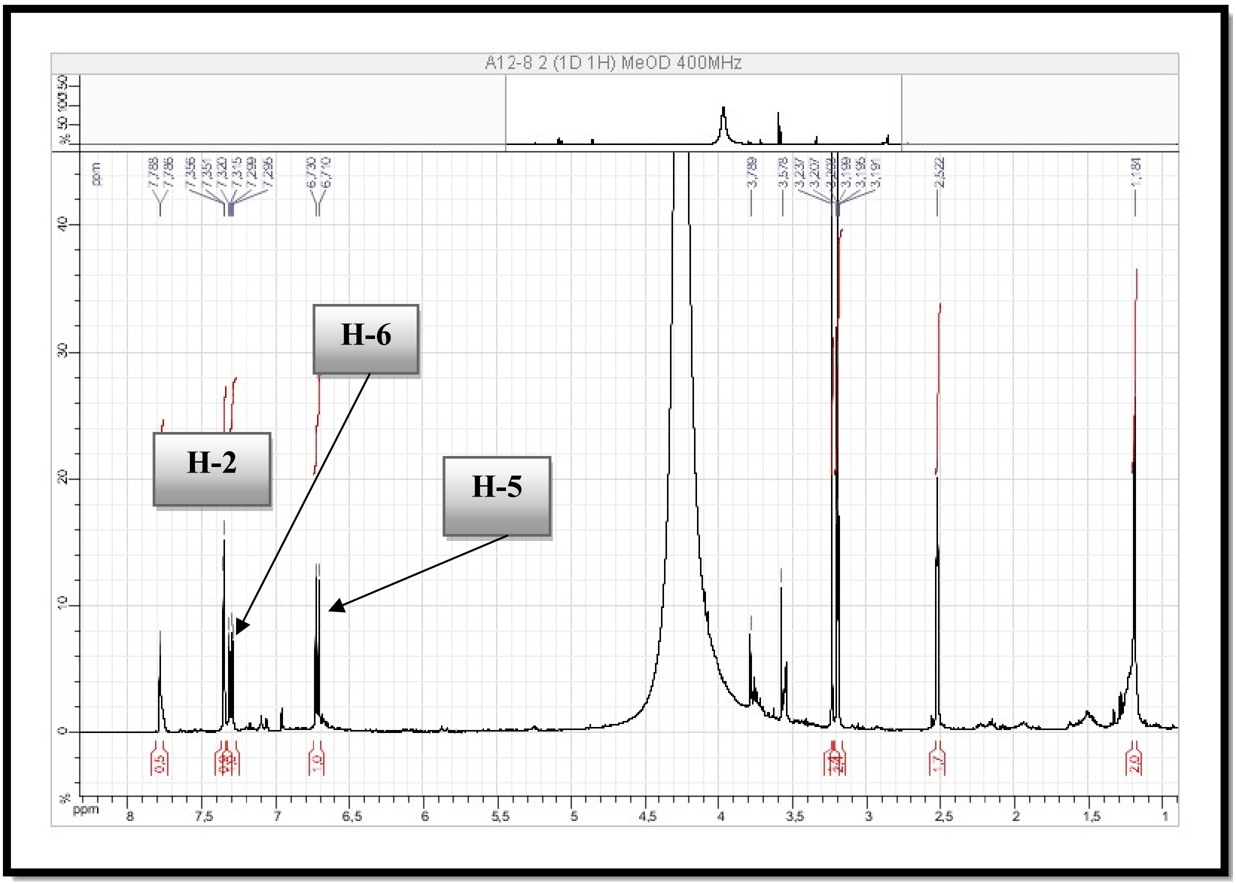

Figure.13. ^{1}H NMR spectrum (400MHz, CD_3OD, δppm) of protocatechuic acid

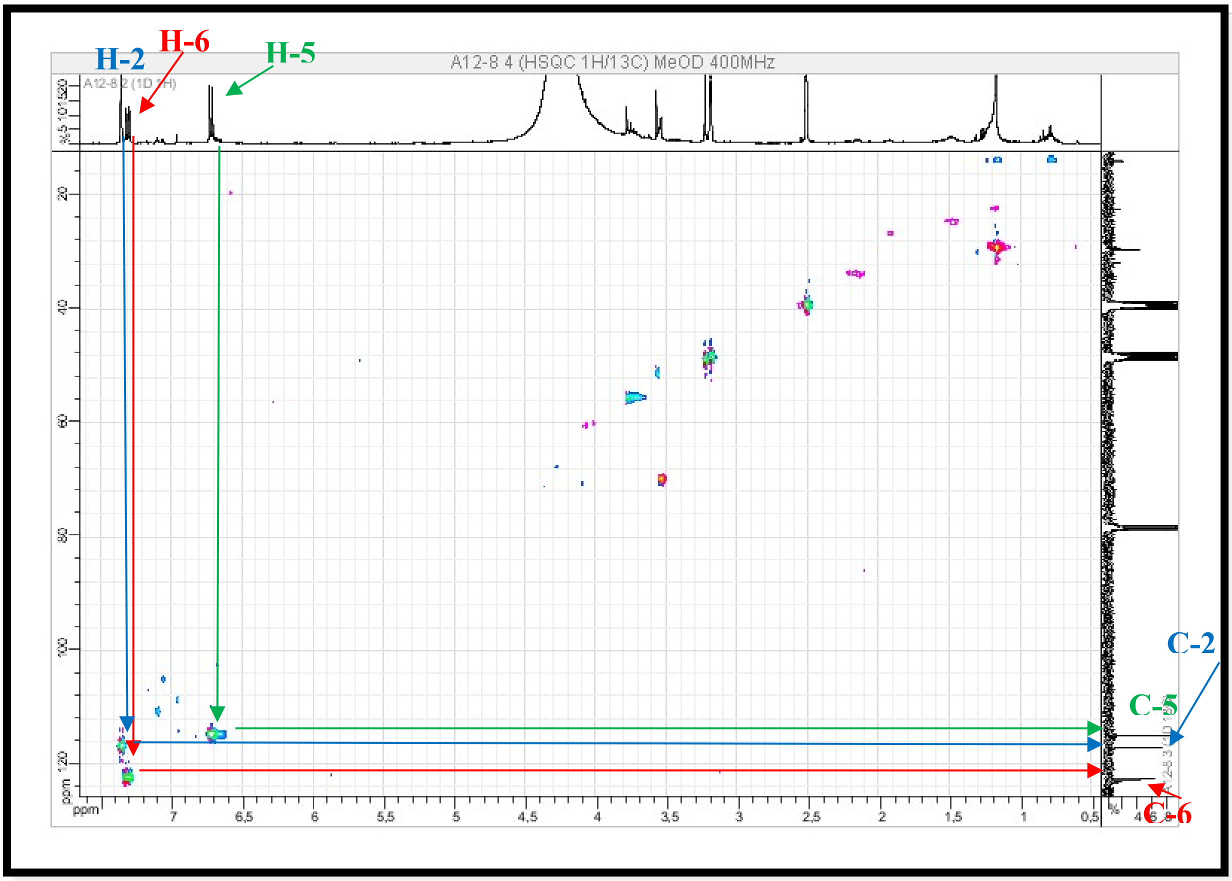

Figure.14. HSQC spectrum (400MHz, CD_3OD, δppm) of protocatechuic acid

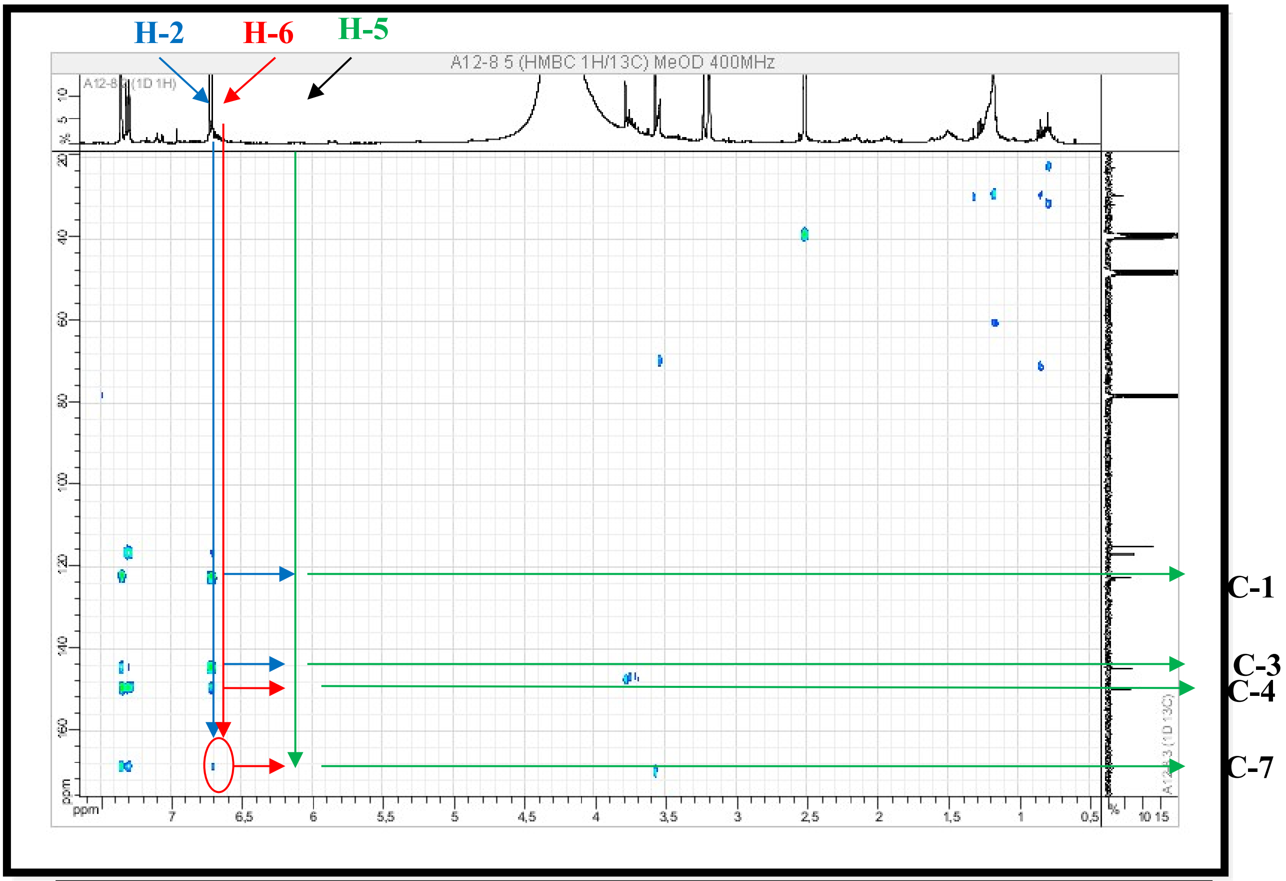

Figure .15.HMBC spectrum (400MHz, CD_3OD, δppm) of protocatechuic acid

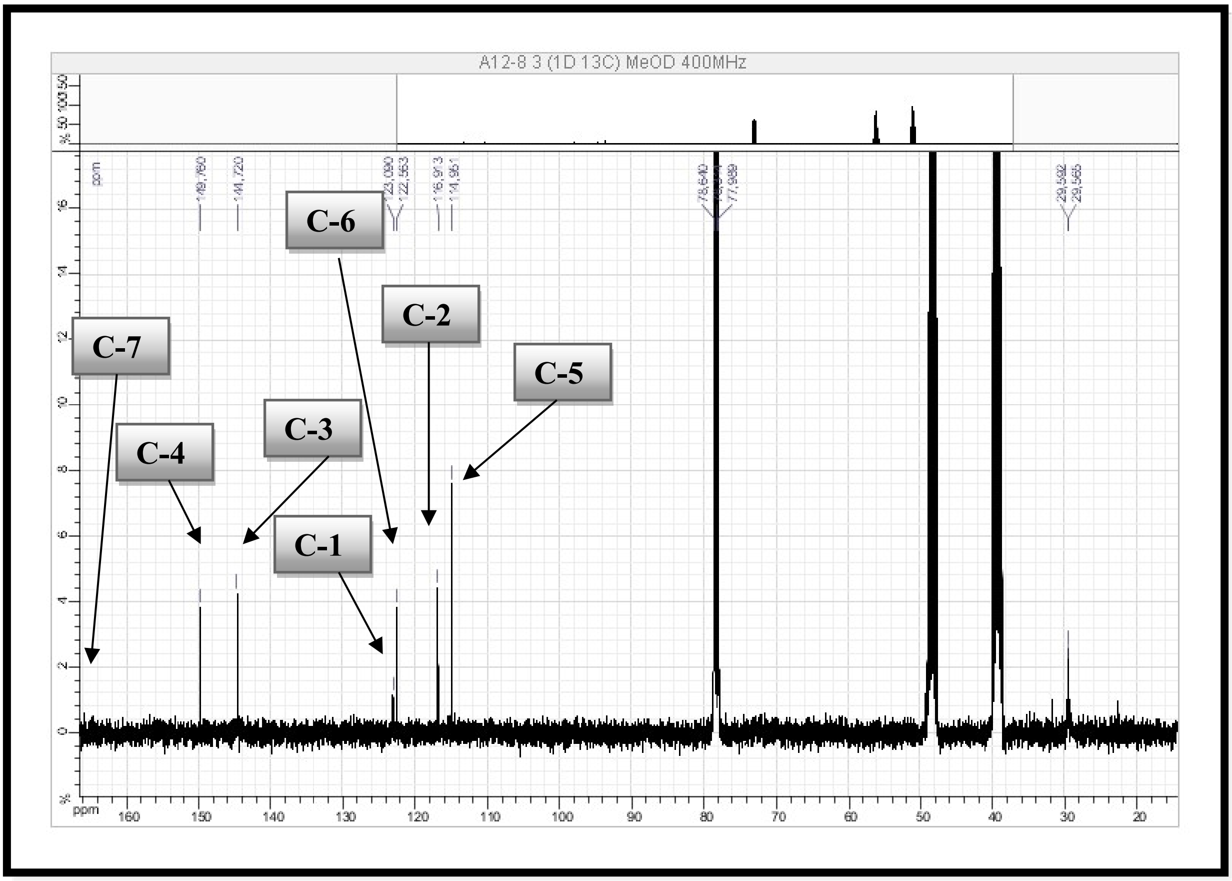

Figure.16. ^{13}C NMR spectrum (100MHz, CD_3OD, δppm) of protocatechuic acid

Molecule 4 : Astragalin

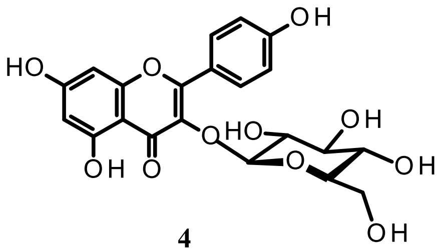

4

Spectrum Source	Fragmentor Voltage	Collision Energy	Ionization Mode
Peak (2) in "+ BPC:1 (all [-200]) Scan"	120	0	ESI

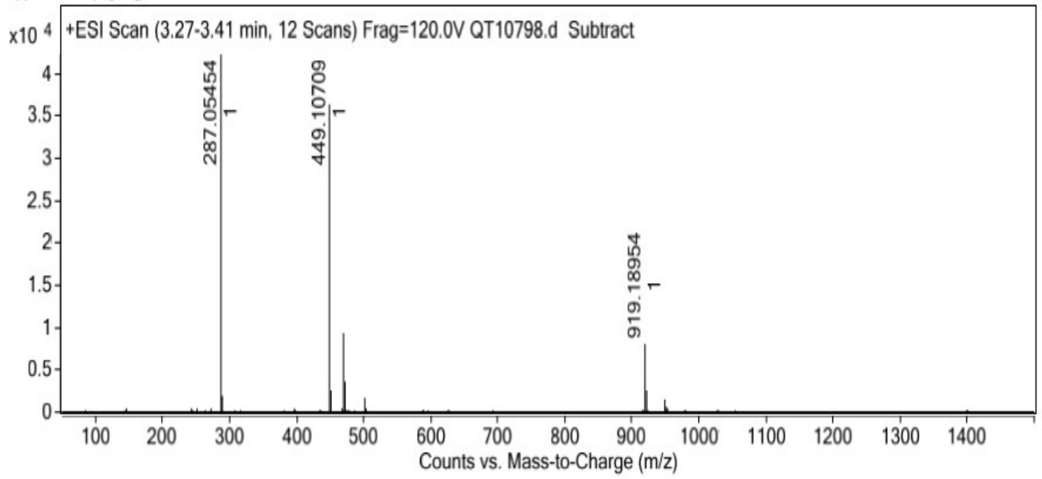

Peak List

m/z	z	Abund	Formula	Ion
287.05454	1	43767.9		(M+H)+
288.0593	1	12794.9		(M+H)+
449.10709	1	36298.9		(M+H)+
450.11158	1	12945.3		(M+H)+
451.11492	1	2487		(M+H)+
471.08895	1	9379.5		
472.09355	1	3573.7		
919.18954	1	8100.6		
920.19365	1	6621.5		
921.19707	1	2599.4		

Figure.17. HRESI-MS (+) of astragalin

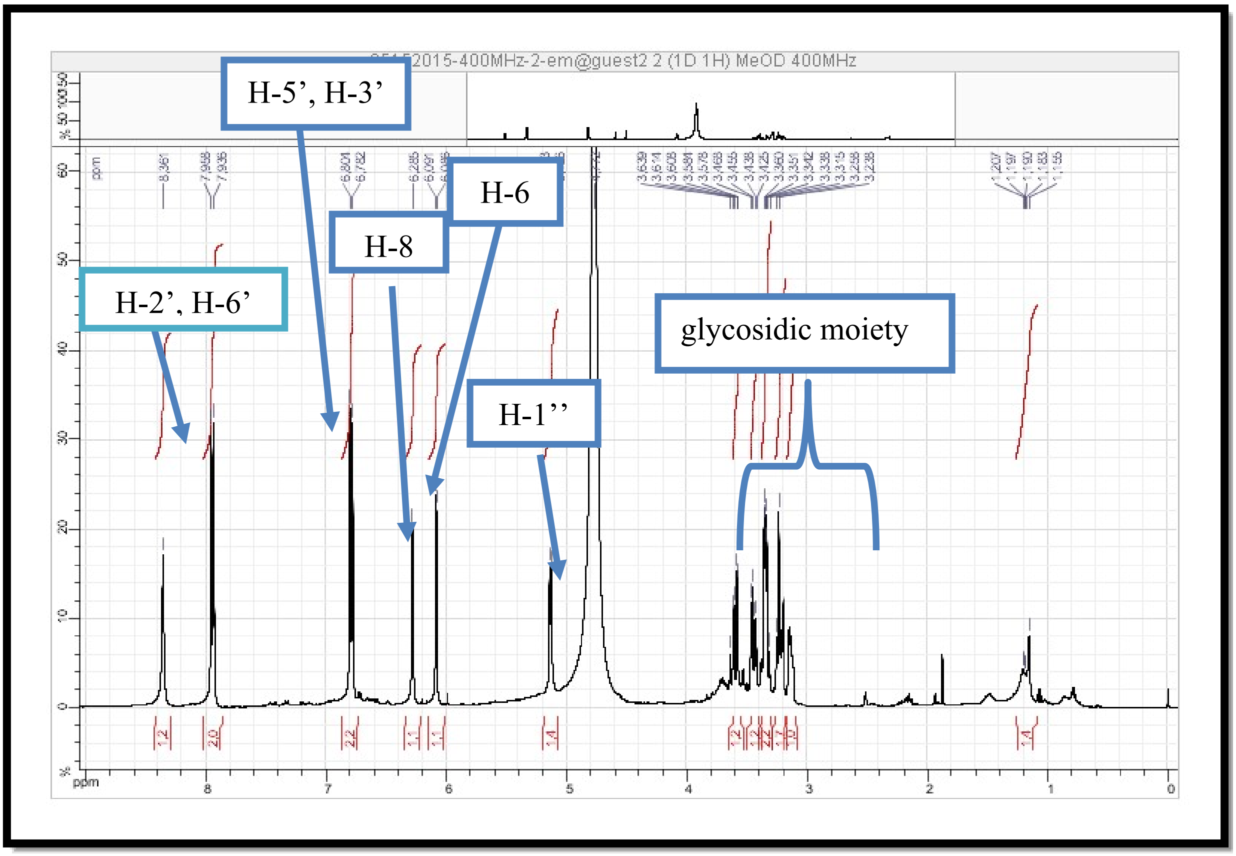

Figure.18. ^{1}H NMR spectrum (400MHz, CD_3OD, δppm) of astragalin

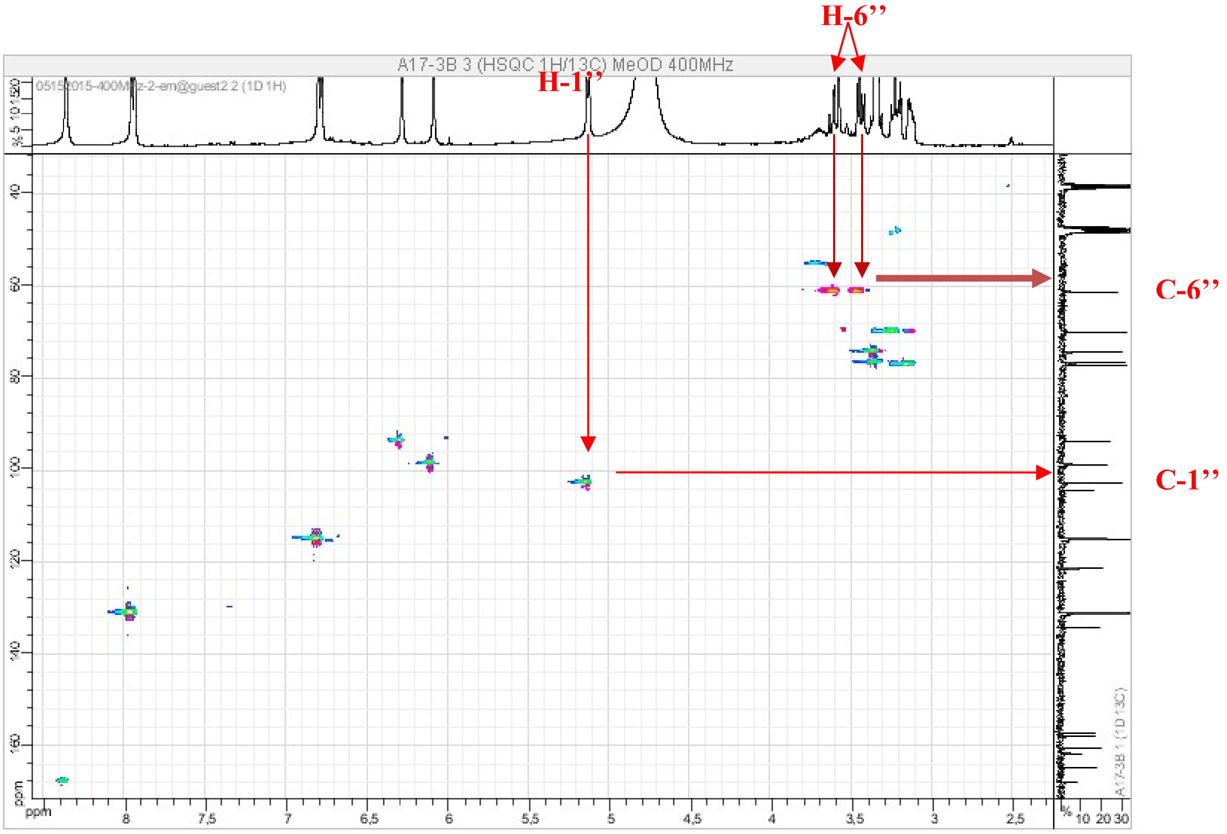

Figure.19. HSQC spectrum (400MHz, CD_3OD, δppm) of astragalin

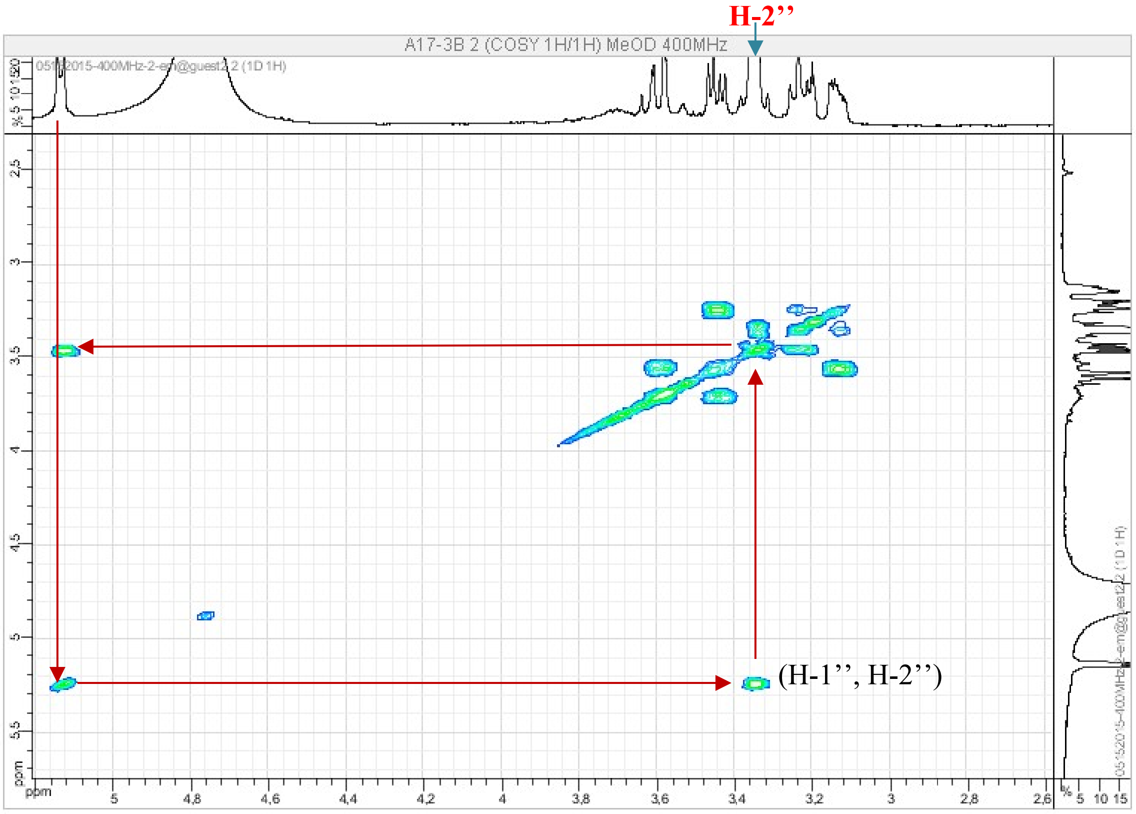

Figure.20. COSY spectrum (400MHz, CD_3OD, δppm) of astragalin

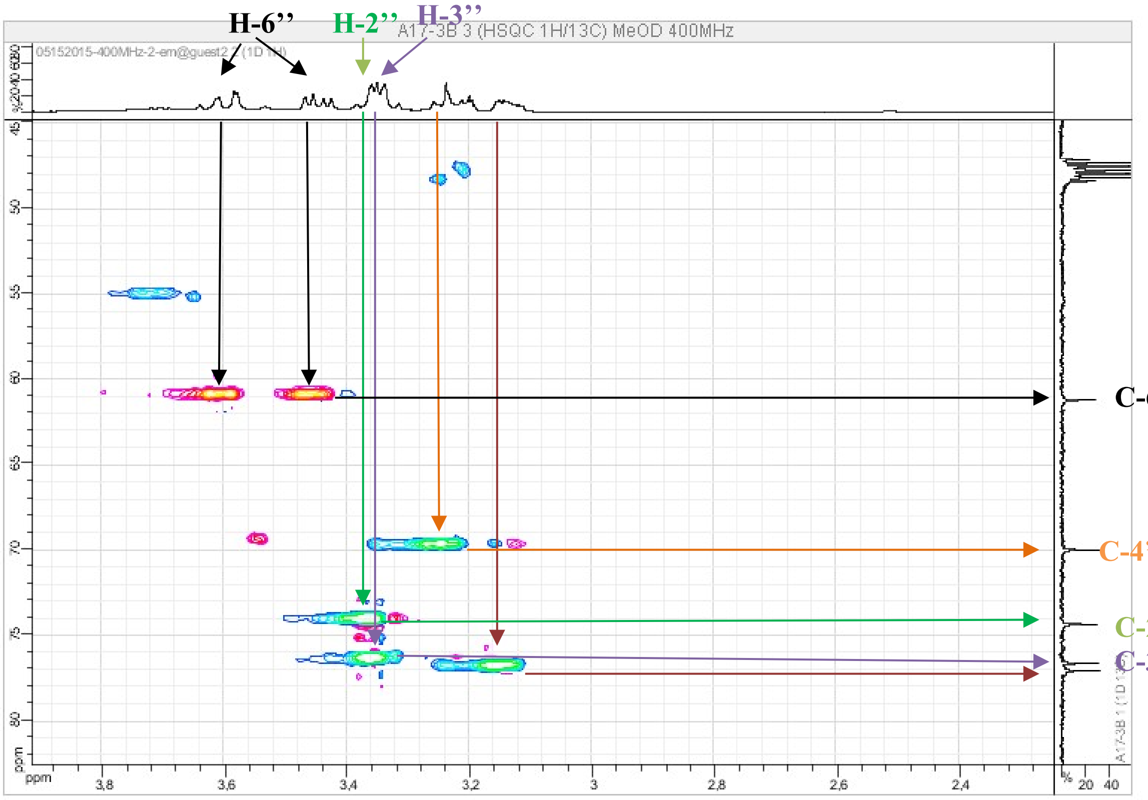

Figure.21. HSQC spectrum (400MHz, CD_3OD, δppm) of astragalin

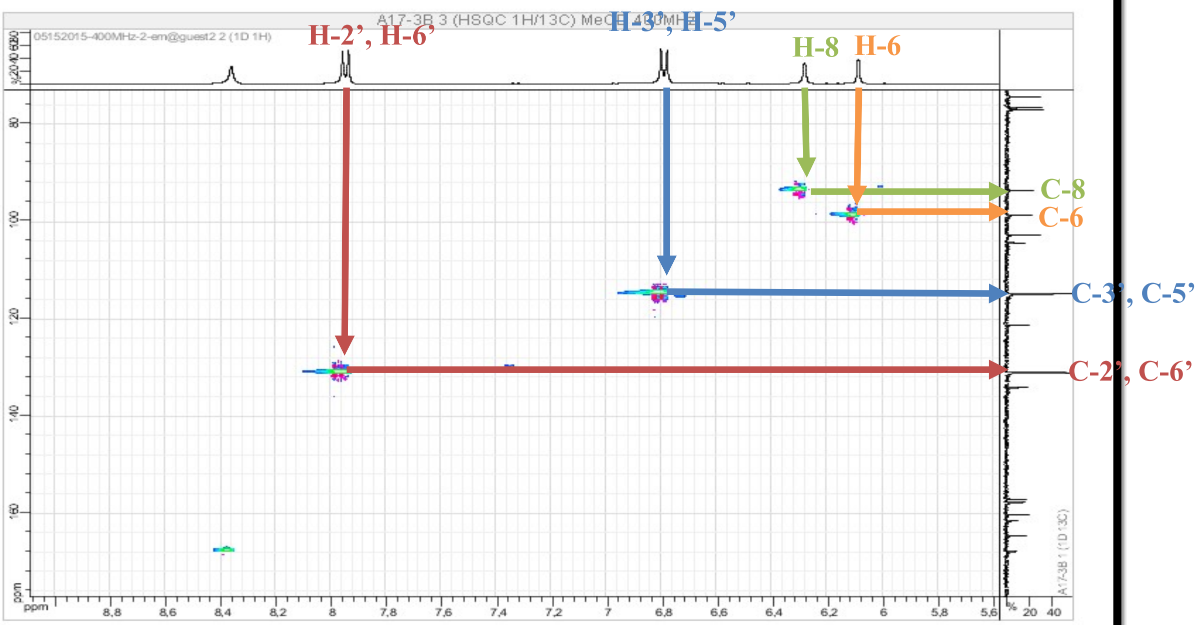

Figure.22. HSQC spectrum (spreading out 1) (400MHz, CD_3OD, δppm) of astragalin

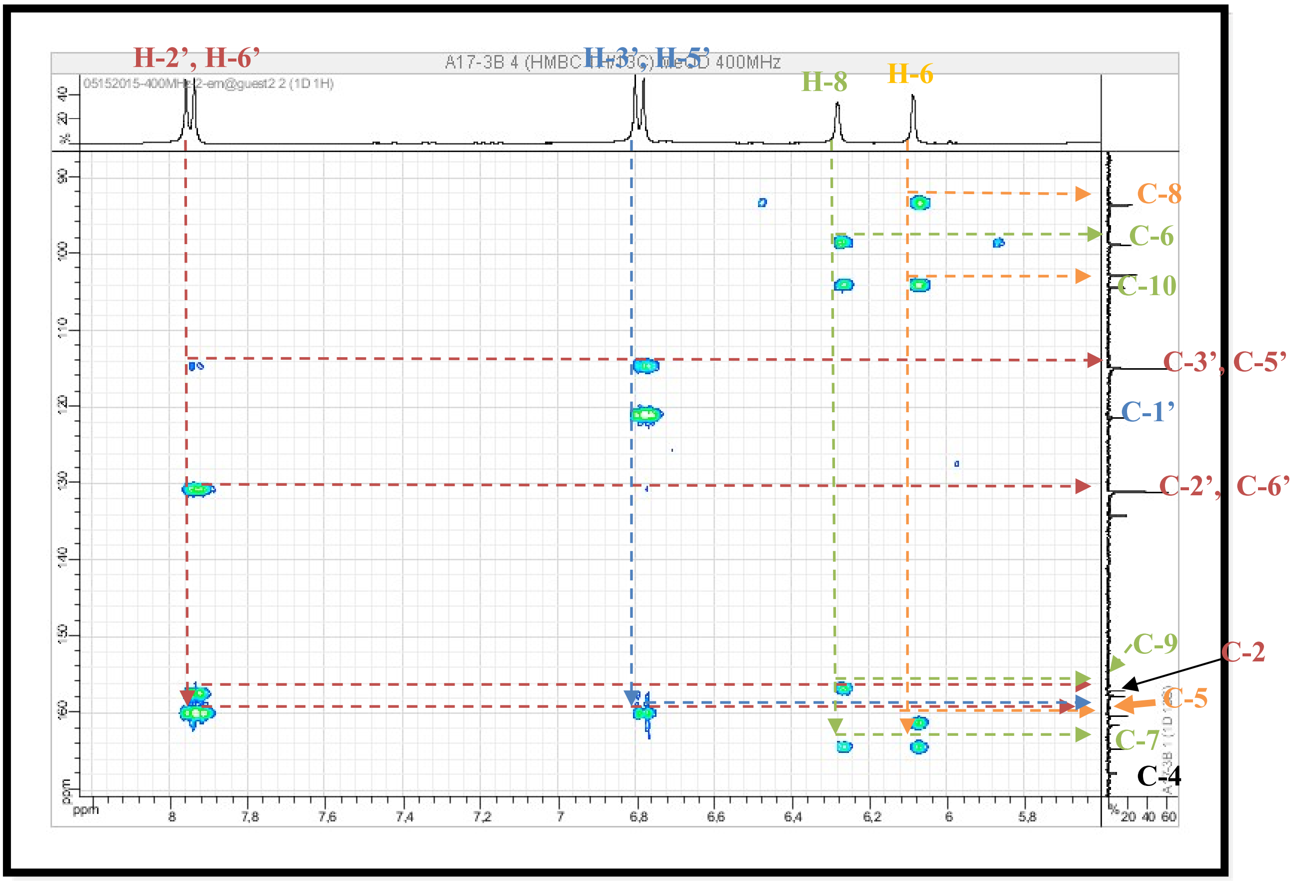

Figure.23. HMBC spectrum (spreading out1) (400MHz, CD_3OD, δppm) of astragalin

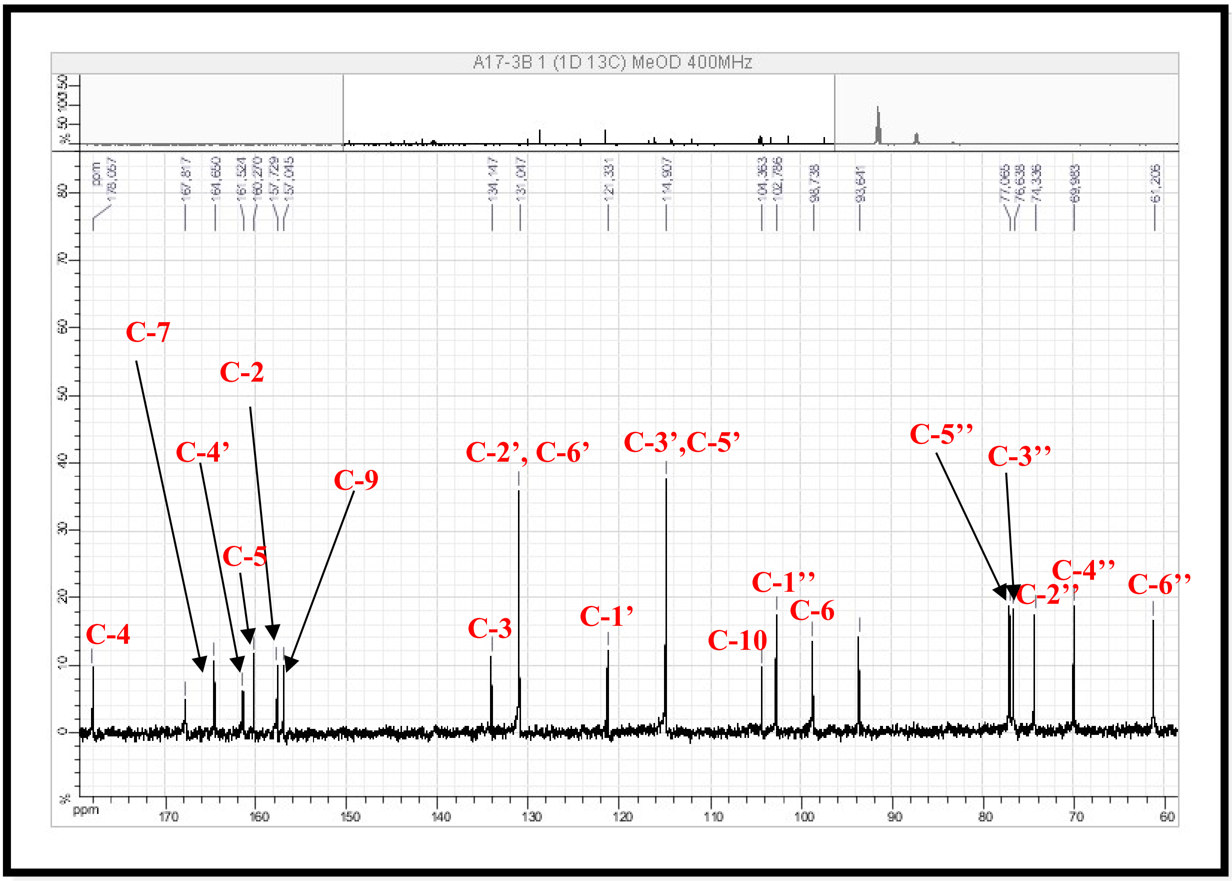

Figure.24. ^{13}C spectrum (100MHz, CD_3OD, δppm) of astragalin

Molecule 5: *Trans*-tiliroside

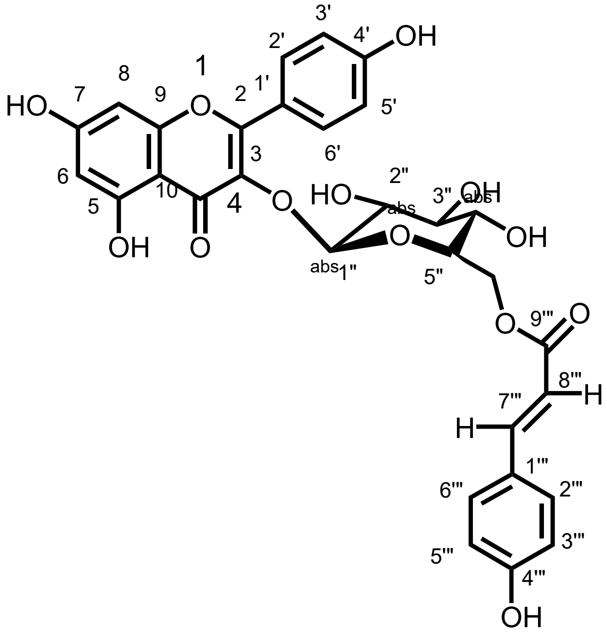

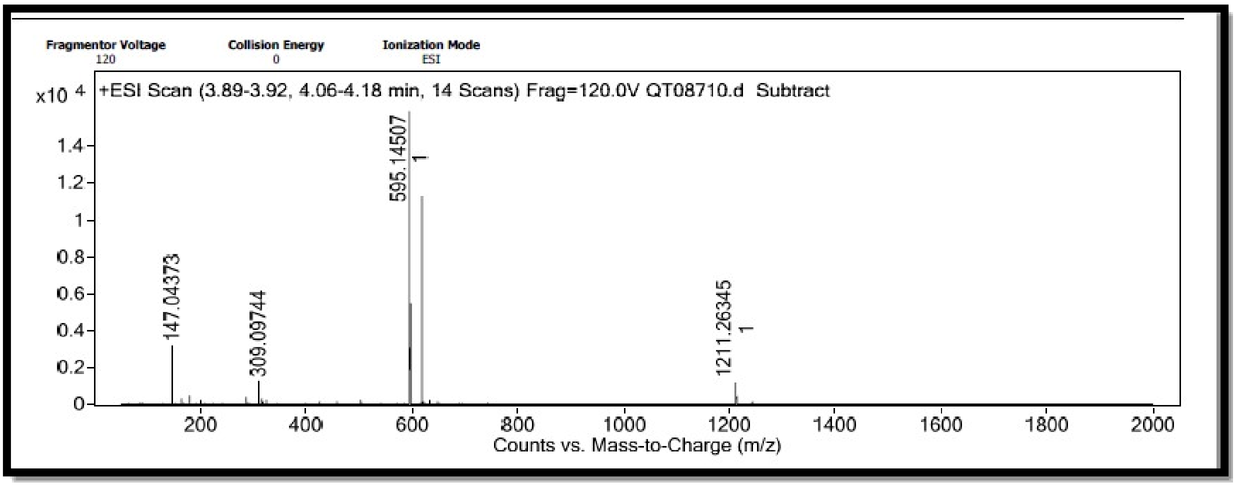

Figure.25.HRESI-MS (+) of *trans*-tiliroside

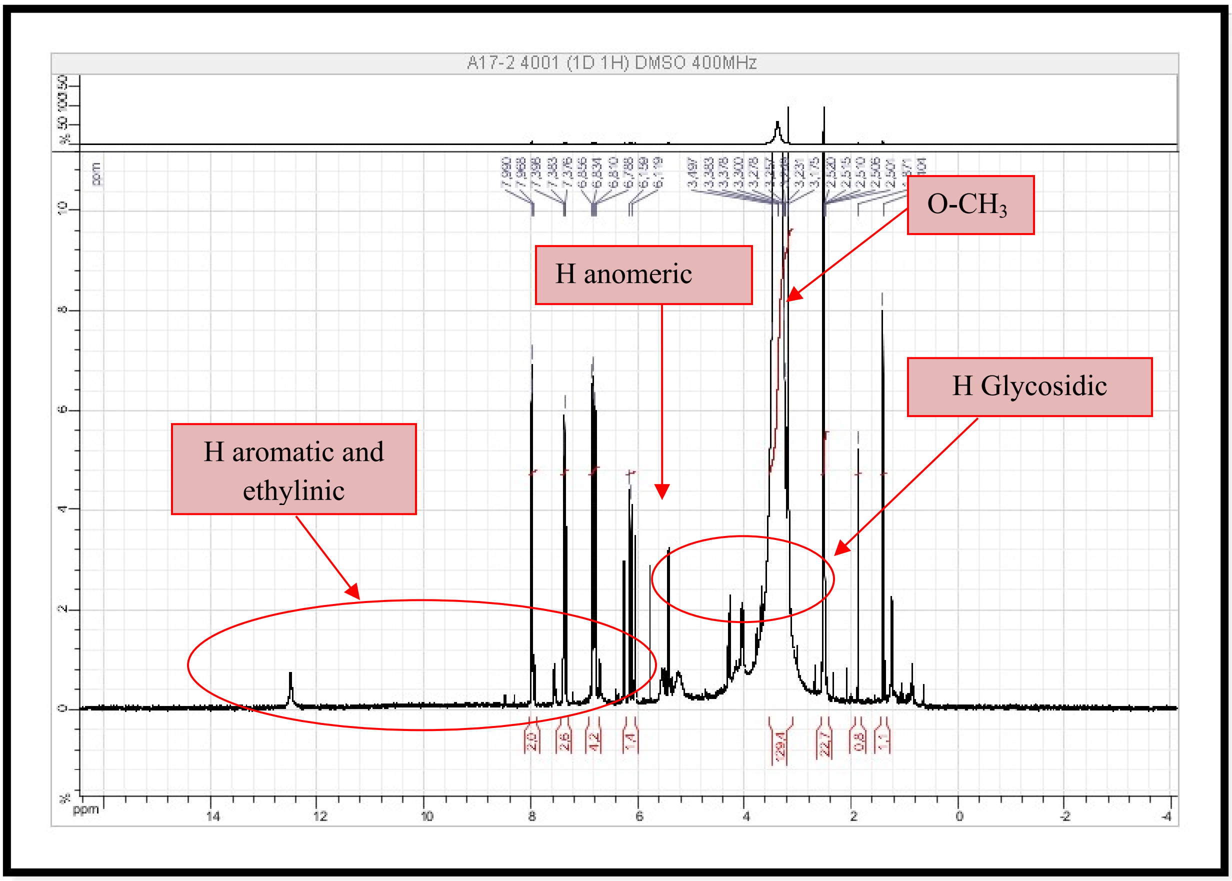

Figure.26. ^{1}H NMR spectrum (400MHz, DMSO-d_6, δppm) of *trans*-tiliroside

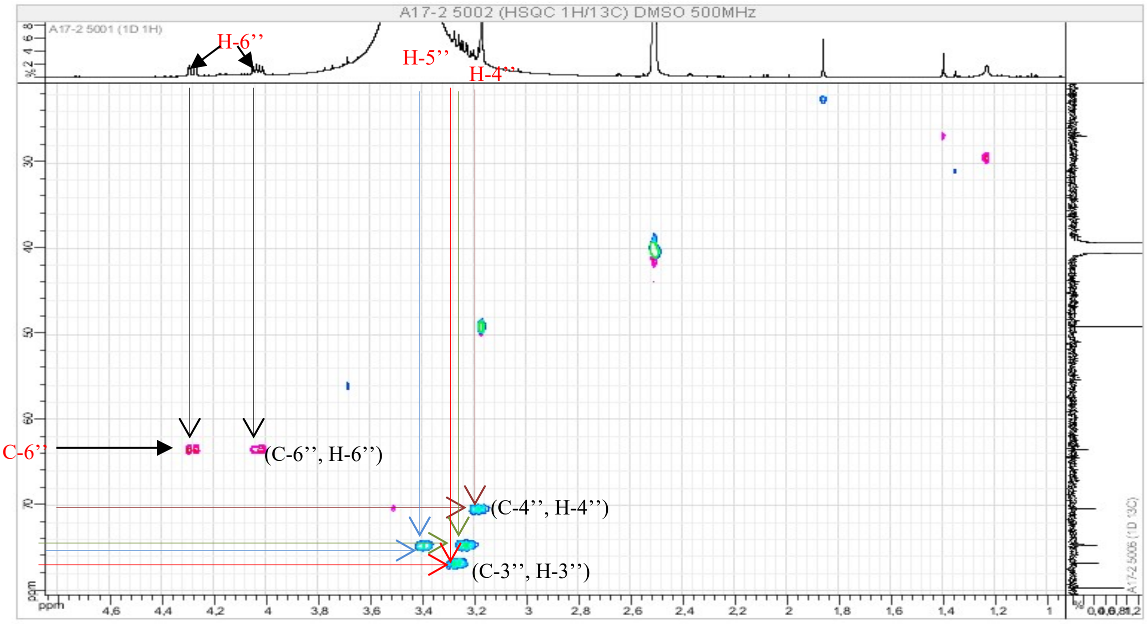

Figure .27. HSQC spectrum (spreading out 1) (500MHz, DMSO-d_6, δppm) of *trans*-tiliroside

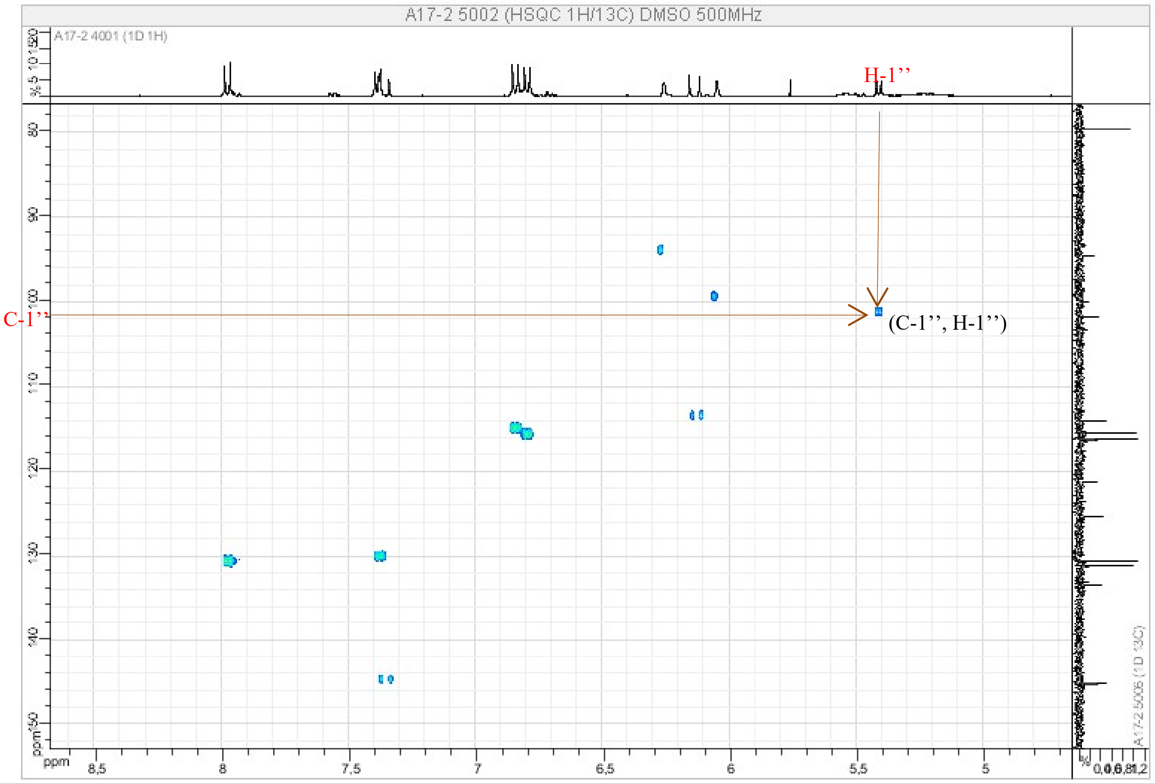

Figure.28. HSQC spectrum (spreading out 2) (500MHz, DMSO-d_6, δppm) of *trans*-

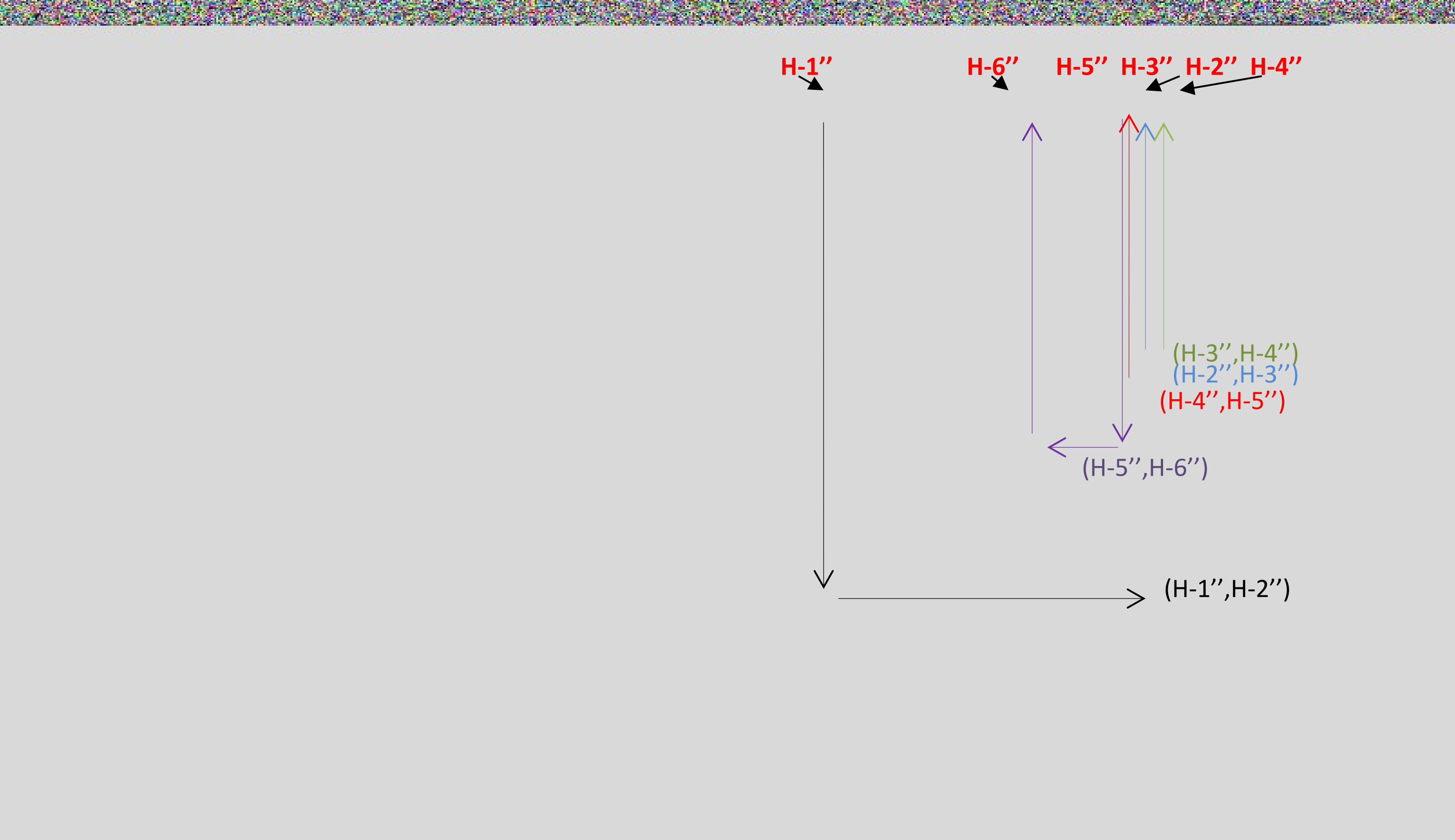

Figure.29. COSY spectrum (spreading out 1) (500MHz, DMSO-d_6, δppm) of *trans*-tiliroside

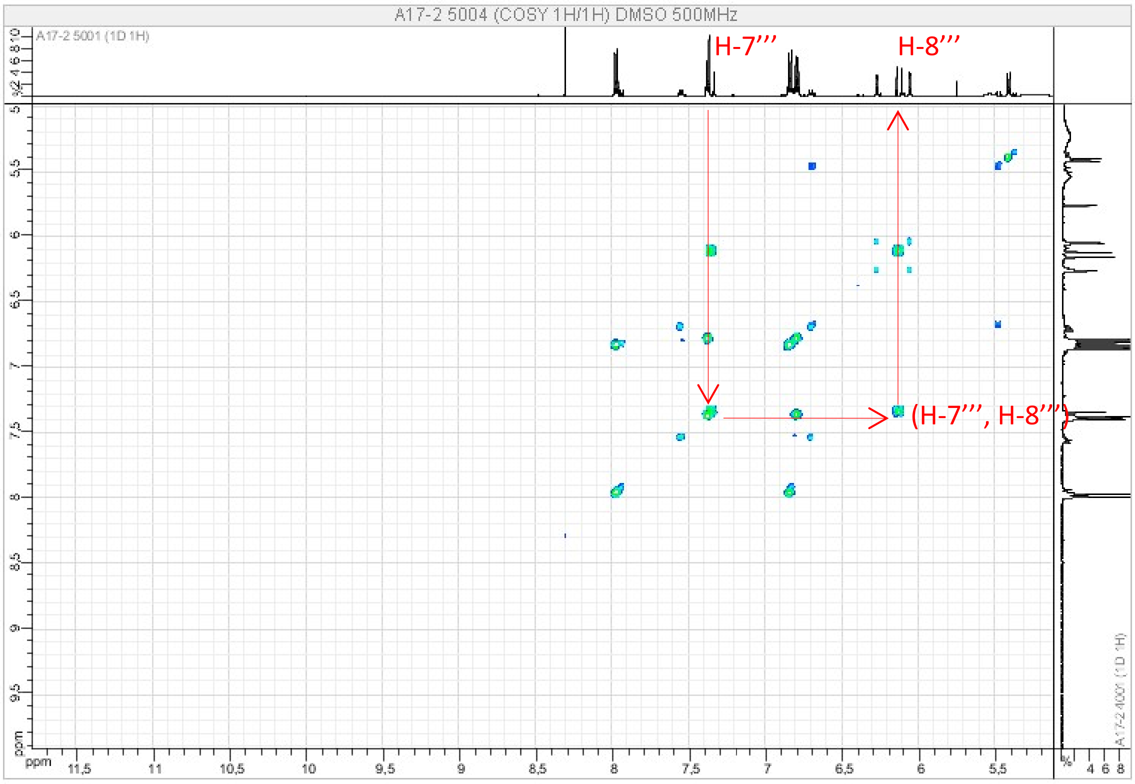

Figure.30. COSY spectrum (spreading out 2) (500MHz, DMSO-d_6, δppm) of *trans*-tiliroside

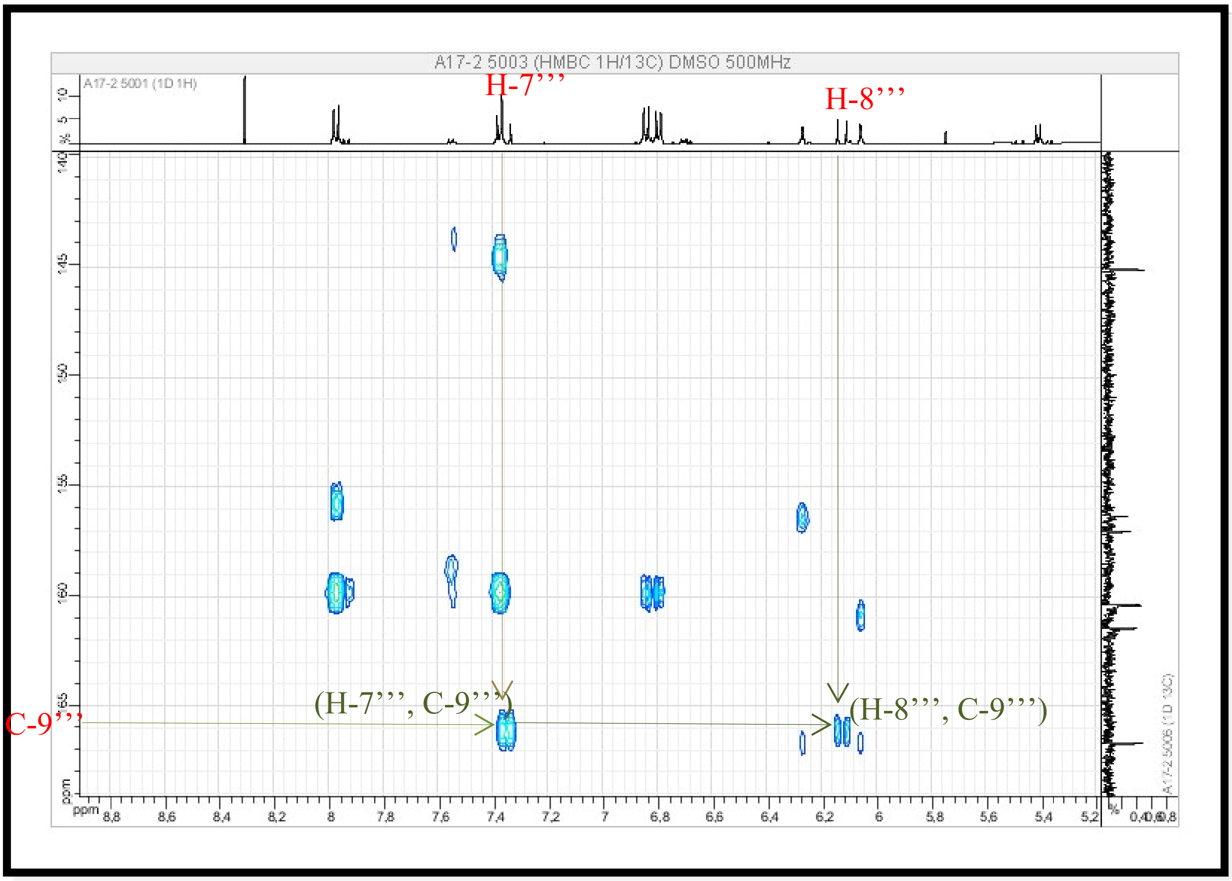

Figure .31. HMBC spectrum (spreading out 1) (500MHz, DMSO-d_6, δppm) of *trans*-tiliroside

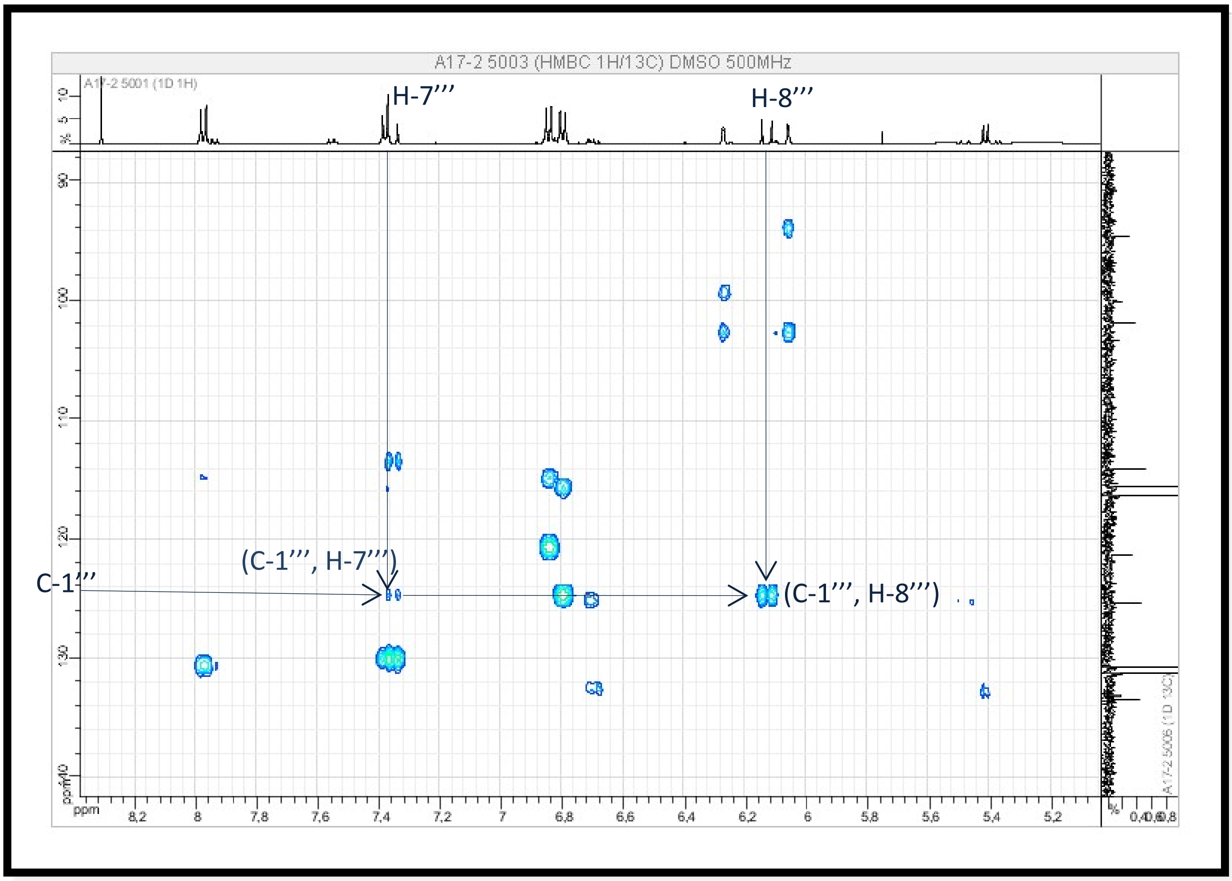

Figure.32. HMBC spectrum (spreading out 2) (500MHz, DMSO-d_6, δppm) of *trans*-tiliroside

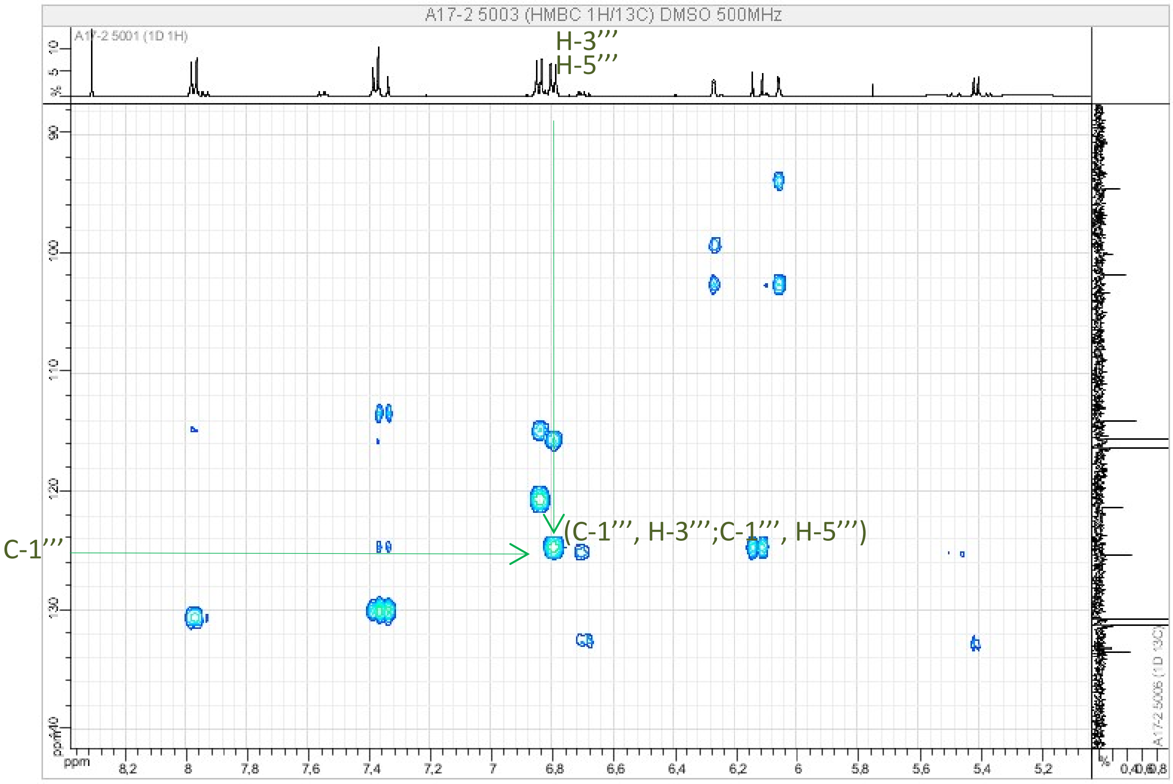

Figure.33. HMBC spectrum (spreading out 3) (500MHz, DMSO-d_6, δppm) of *trans*-tiliroside

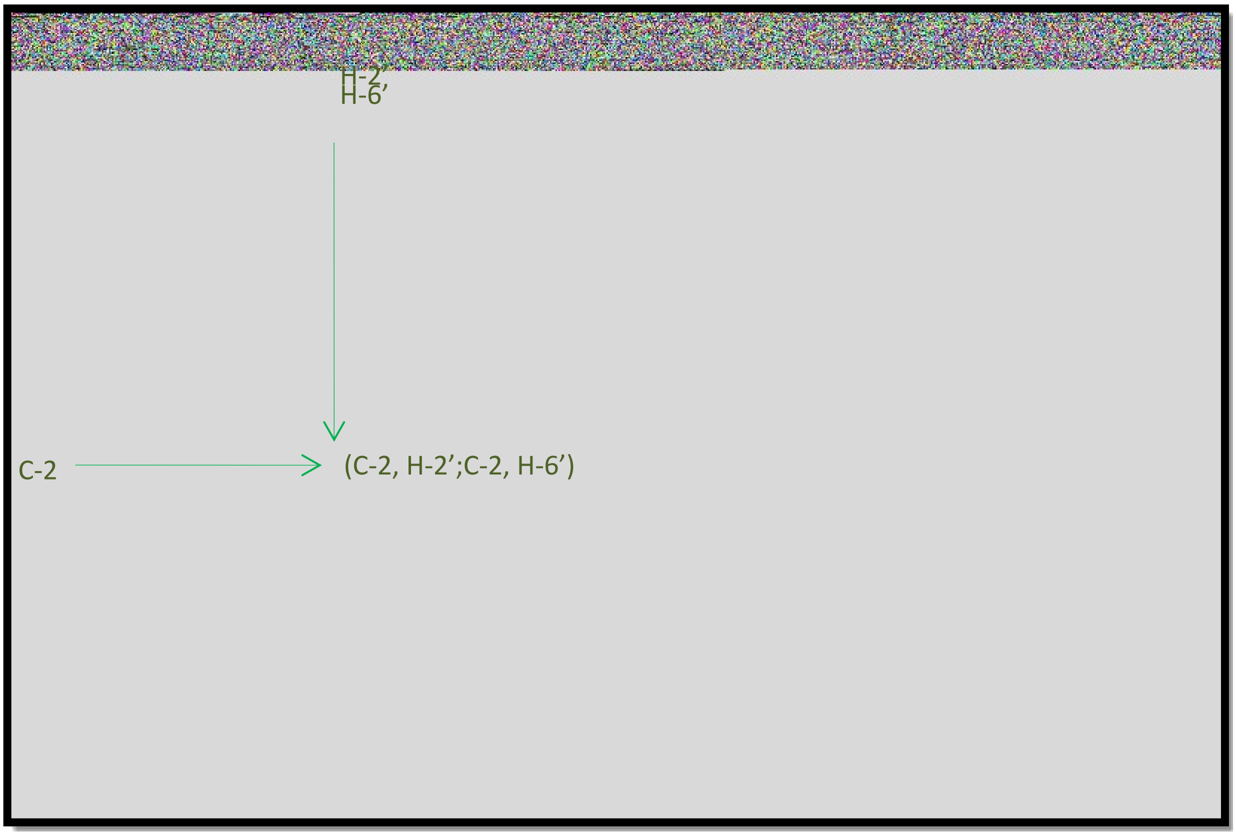

Figure.34. HMBC spectrum (spreading out 4) (500MHz, DMSO-d_6, δppm) of *trans*-tiliroside

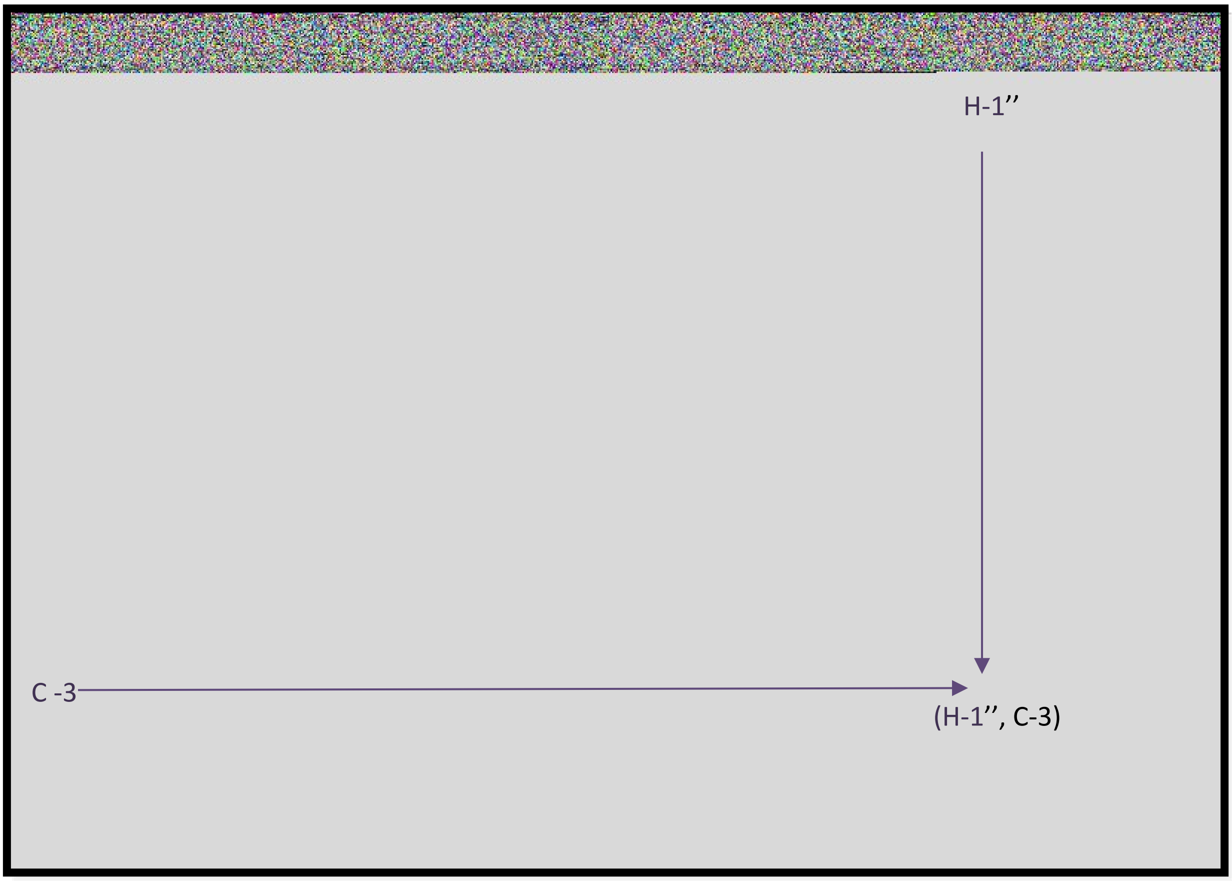

Figure.35.HMBC spectrum (spreading out 5) (500MHz, DMSO-d_6, δppm) of *trans*-tiliroside

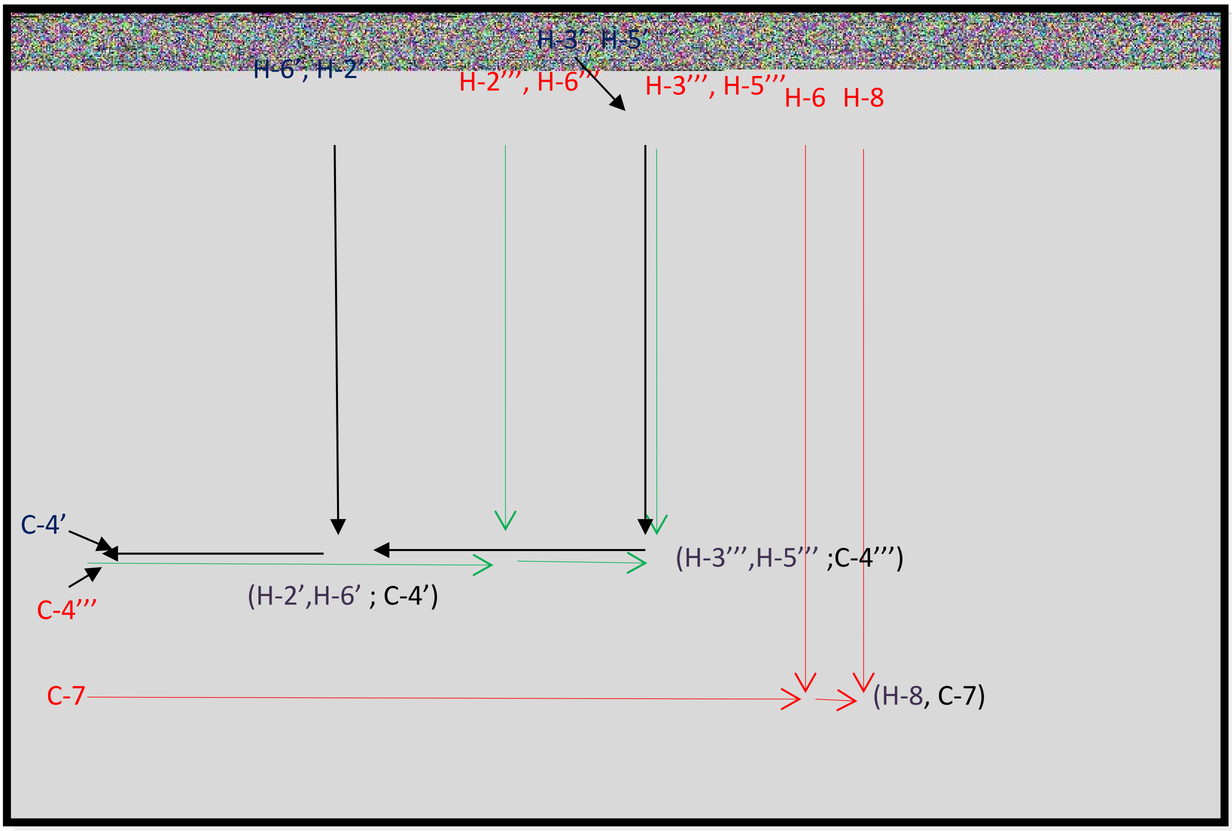

Figure.36.HMBC spectrum (spreading out 6) (500MHz, DMSO-d_6, δppm) of *trans*-tiliroside

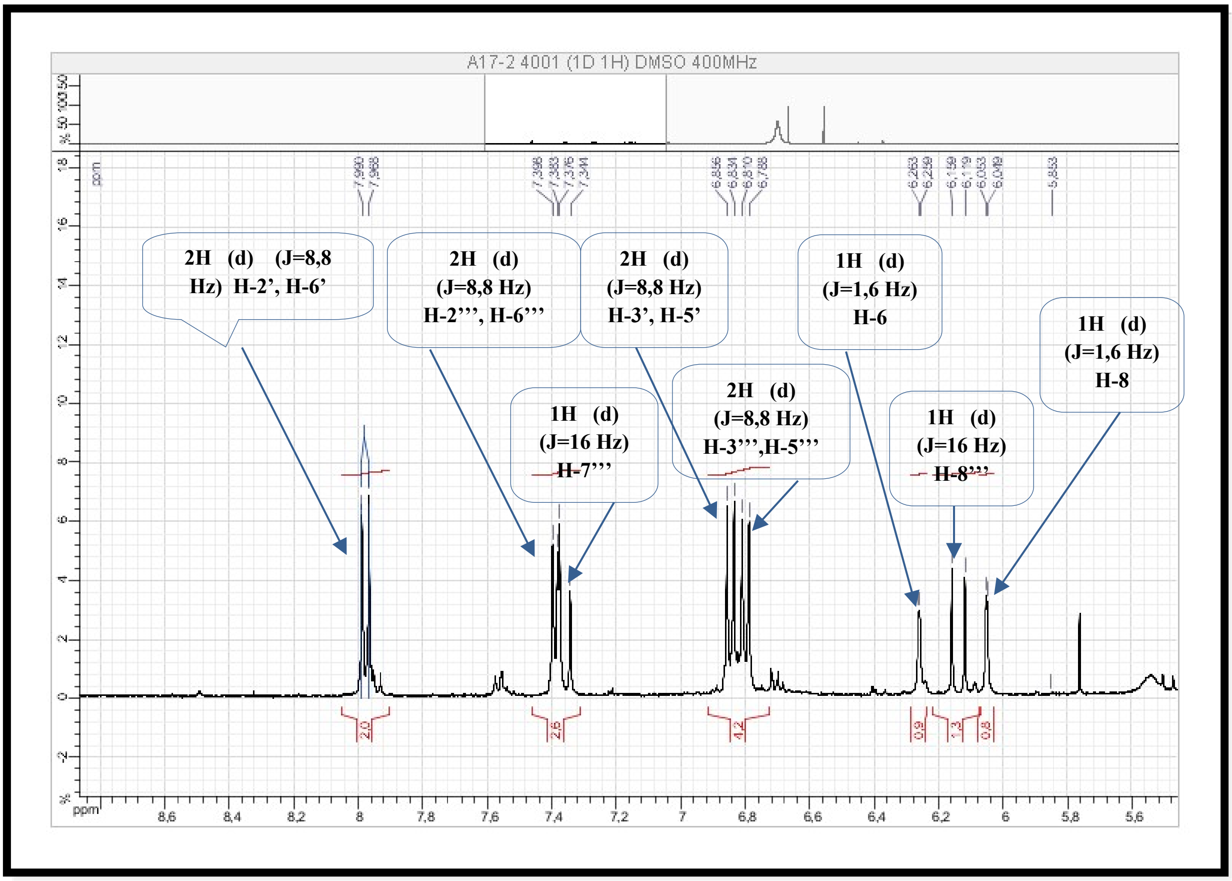

Figure.37.^{1}H NMR spectrum (400MHz, DMSO-d_6, δppm) of *trans*-tiliroside

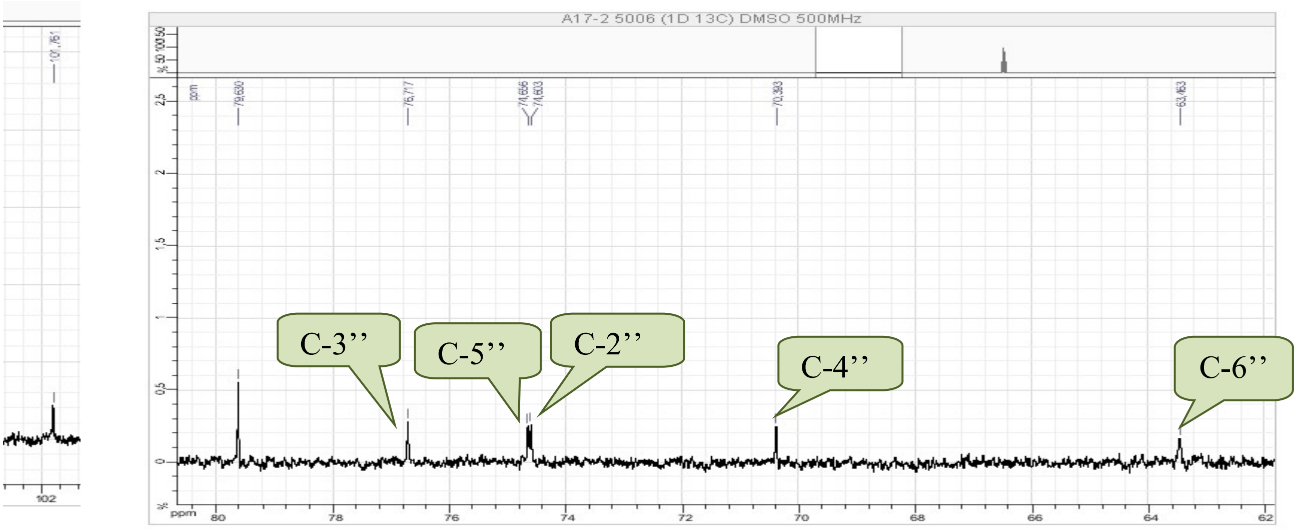

Figure.38. ^{13}C NMR spectrum (spreading out 1) (125MHz, DMSO-d_6, δppm) of *trans*-tiliroside

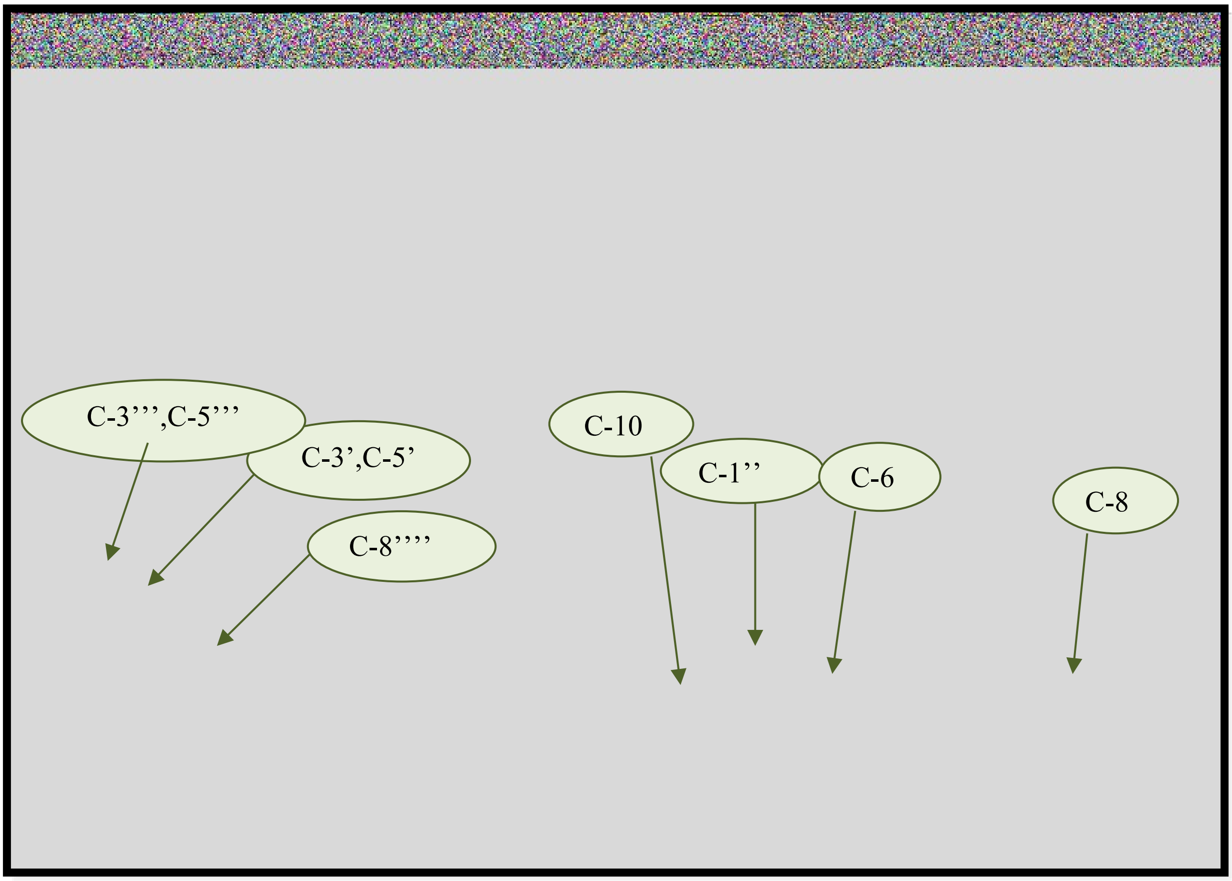

Figure.39. ^{13}C NMR spectrum (spreading out 2) (125MHz, DMSO-d_6, δppm) of *trans*-tiliroside

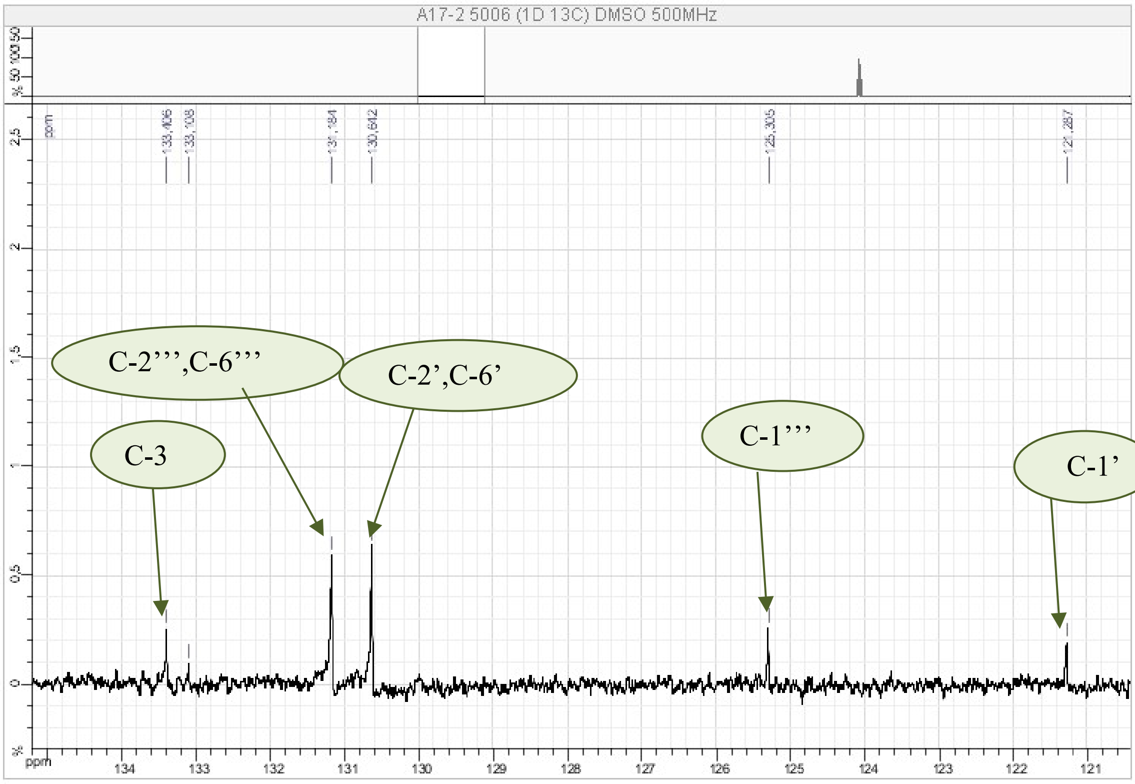

Figure.40. ^{13}C NMR spectrum (spreading out 3) (125MHz, DMSO-d_6, δppm) of *trans*-tiliroside

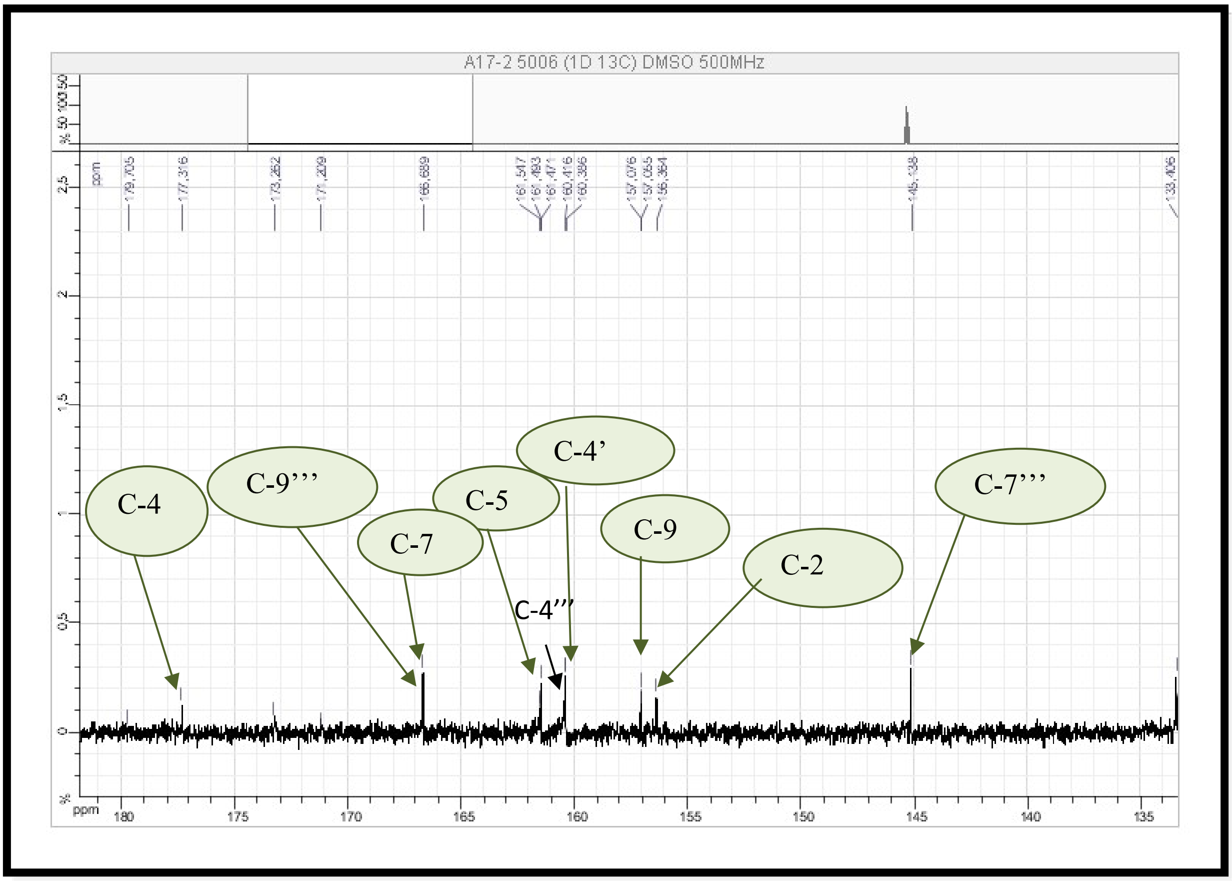

Figure.41.^{13}C NMR spectrum (spreading out 4) (125MHz, DMSO-d_6, δppm) of *trans*-tiliroside

Molecule 5 : *Cis*-tiliroside

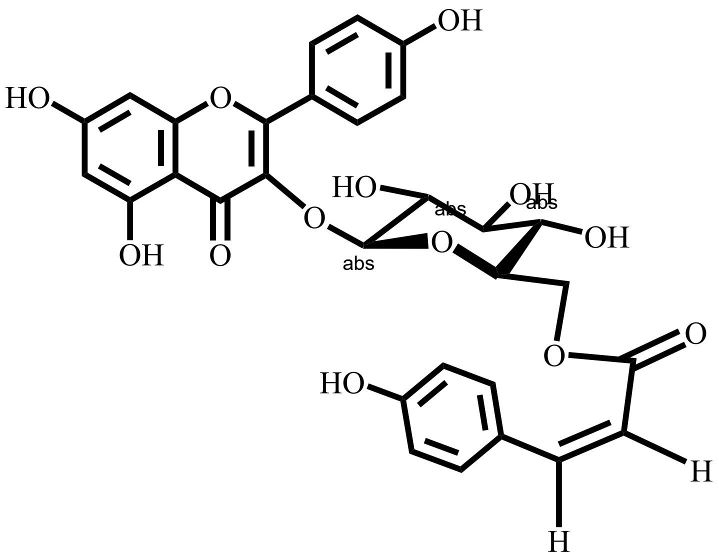

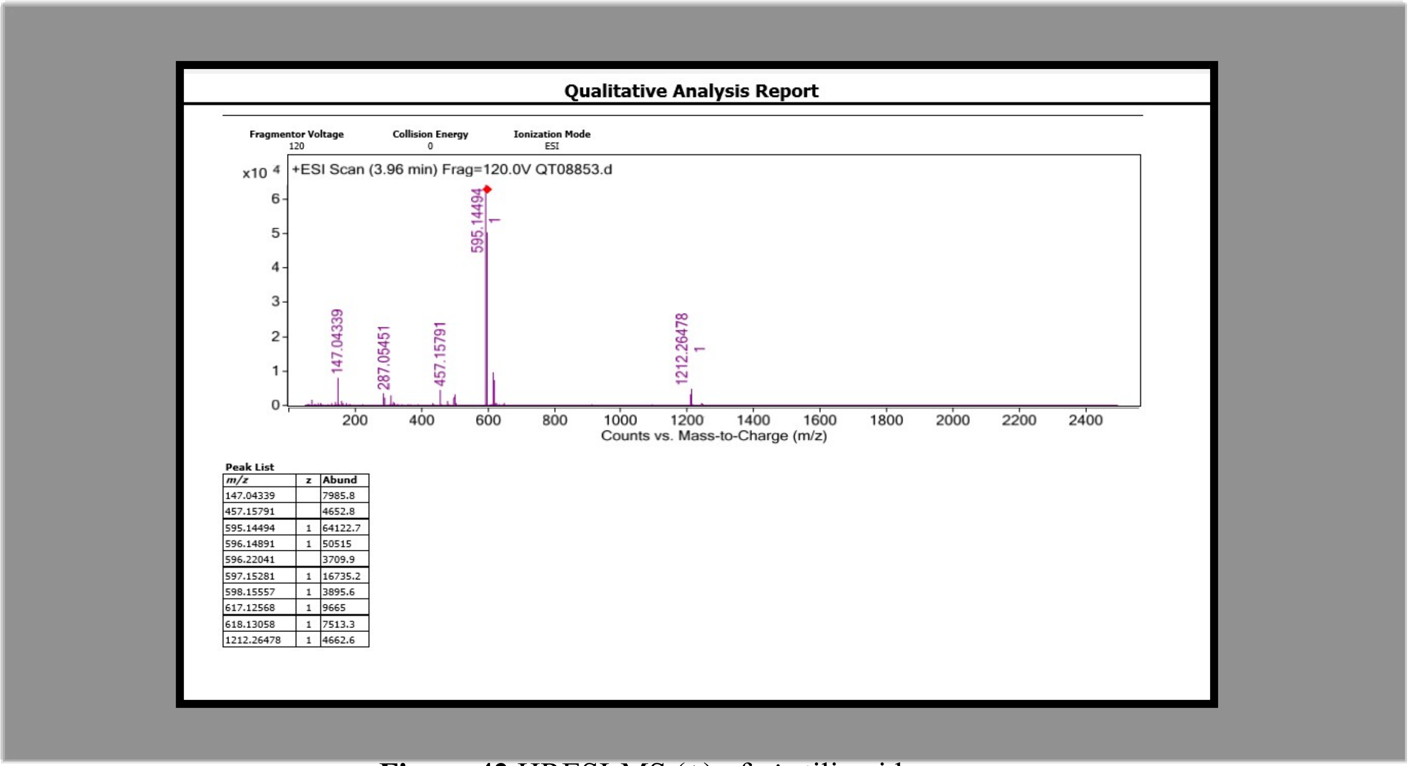

m/z	z	Abund
147.04339		7985.8
457.15791		4652.8
595.14494	1	64122.7
596.14891	1	50515
596.22041		3709.9
597.15281	1	16735.2
598.15557	1	3895.6
617.12568	1	9665
618.13058	1	7513.3
1212.26478	1	4662.6

Figure.42.HRESI-MS (+) of *cis*-tiliroside

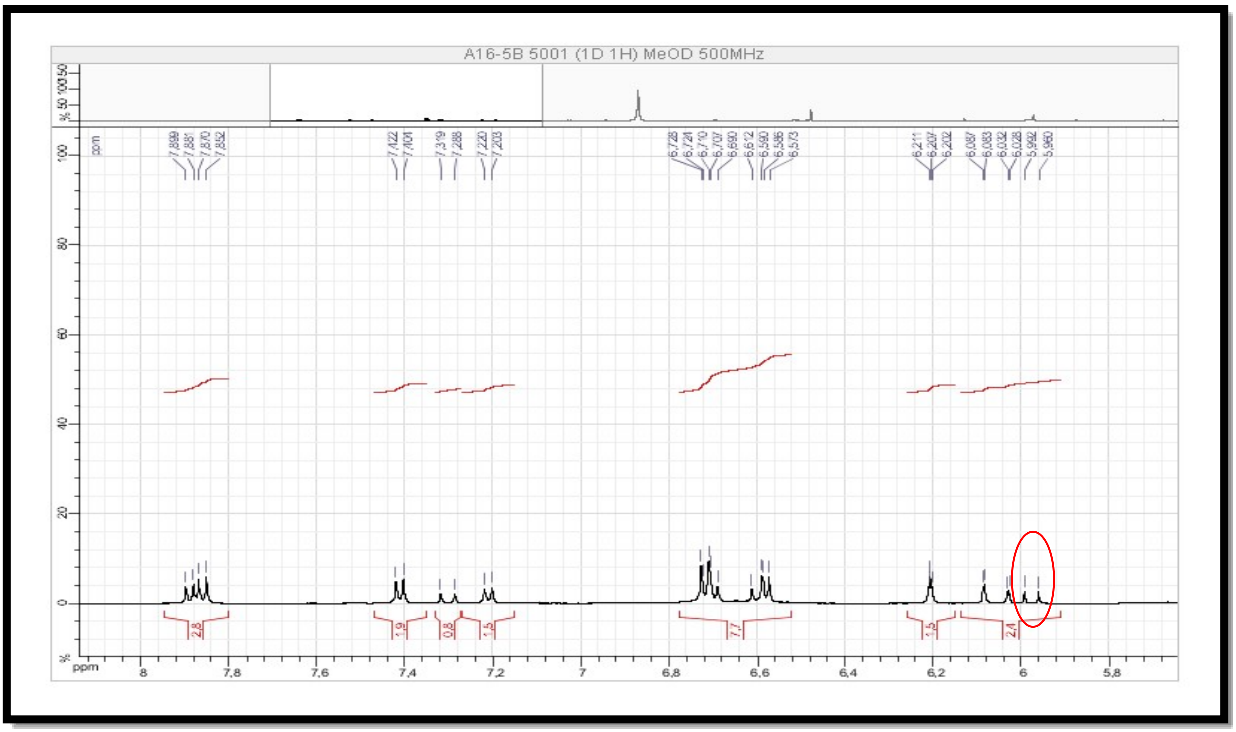

Figure.43. ^{1}H NMR spectrum (spreading out1) (500MHz, CD_3OD, δppm) of *cis*-tiliroside

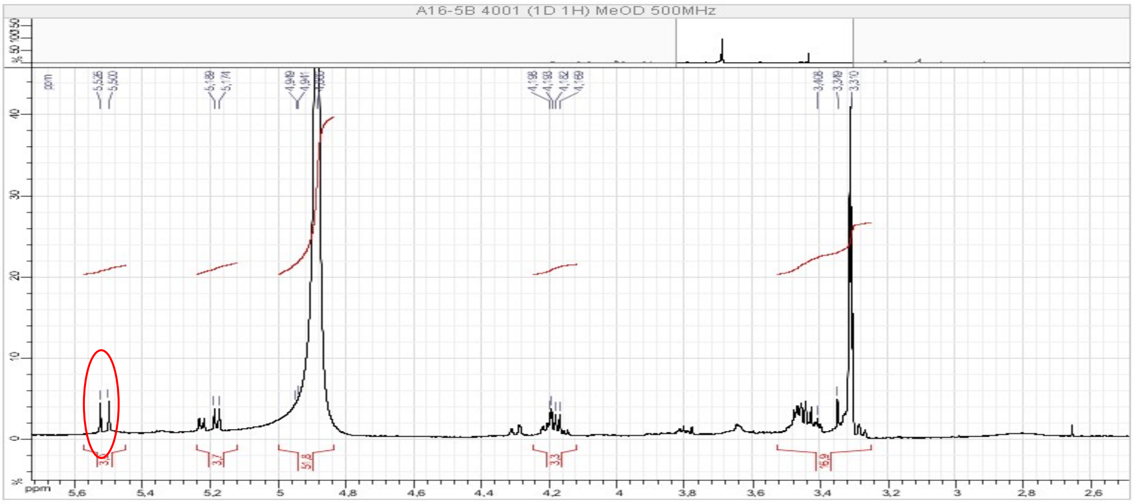

Figure.44. ¹H NMR spectrum (spreading out 2) (500MHz, CD_3OD, δppm) of *cis*-tiliroside

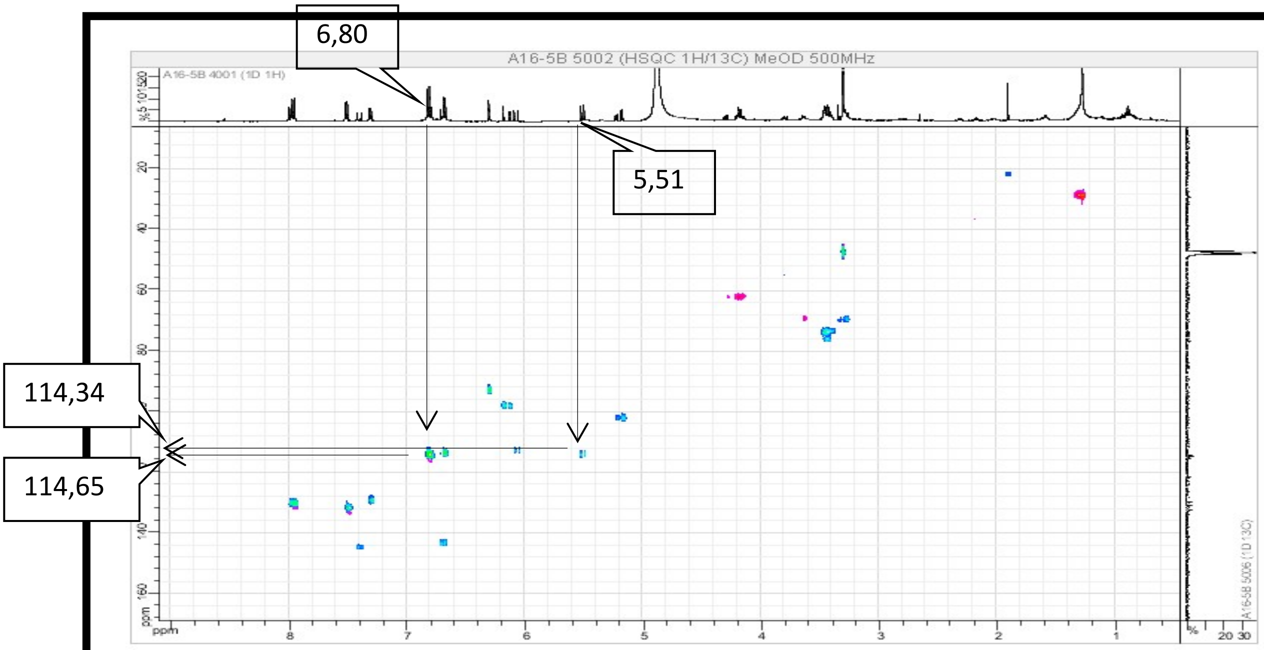

Figure.45. HSQC NMR spectrum (500MHz, CD_3OD, δppm) of *cis*-tiliroside

Molecule 7 : 3-oxo-α-ionol-β-D-glucopyranoside

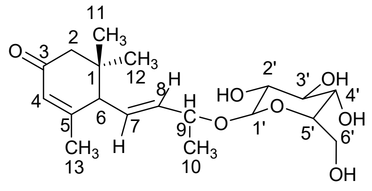

(7)

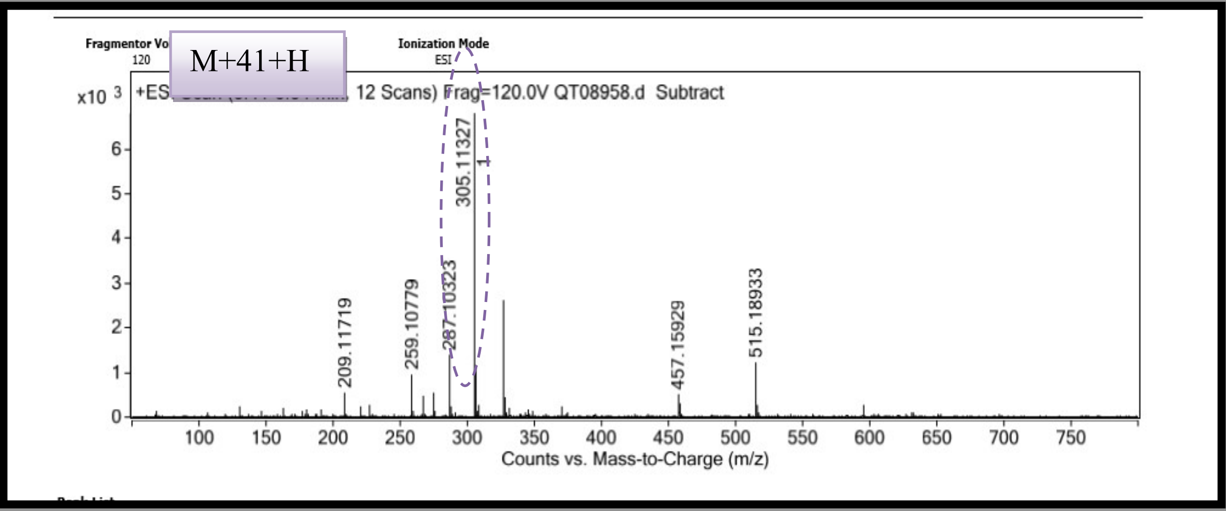

Figure.46.HRESI-MS (+) of 3-oxo-α-ionol-β-D-glucopyranoside

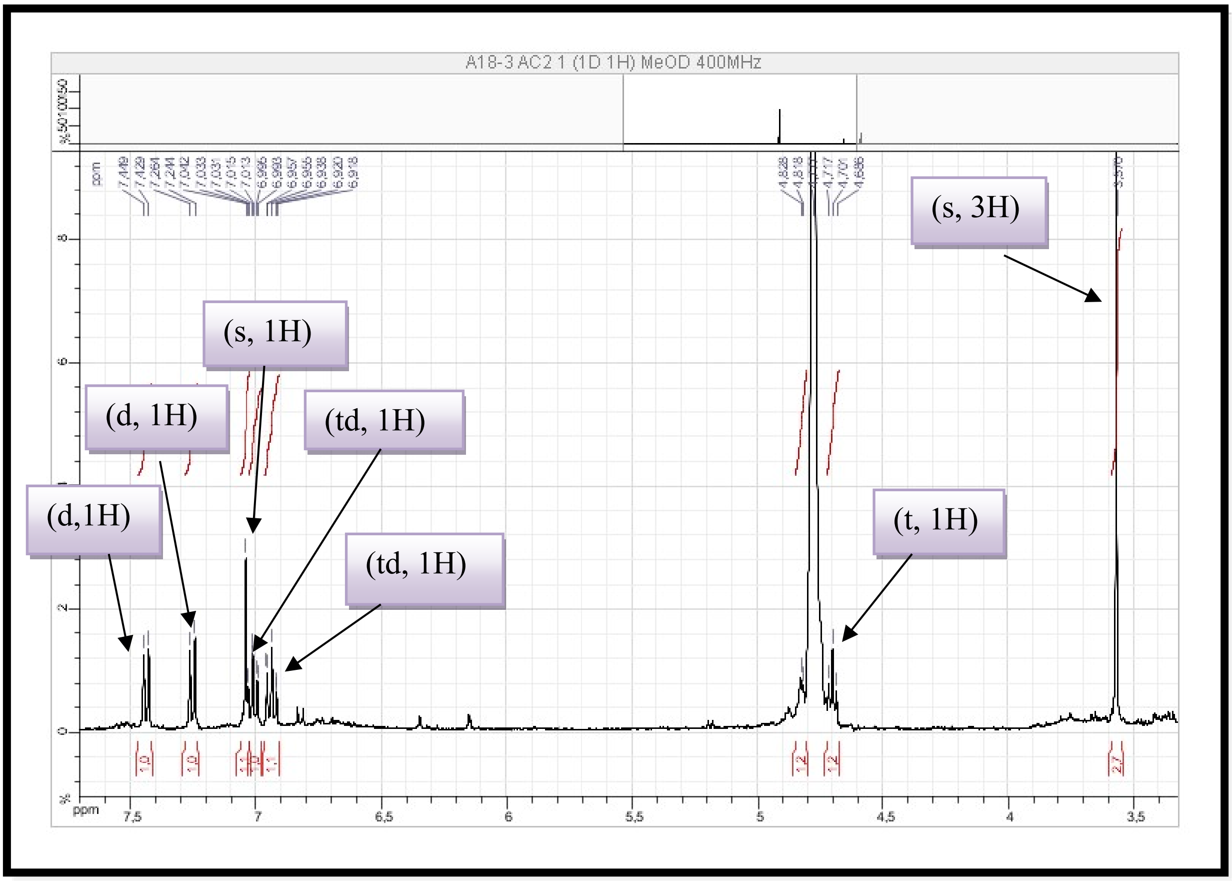

Figure.47-1. ¹H NMR spectrum (spreading out1) (400MHz, CD_3OD, δ_{ppm}) of 3-oxo-α-ionol-β-D-glucopyranoside

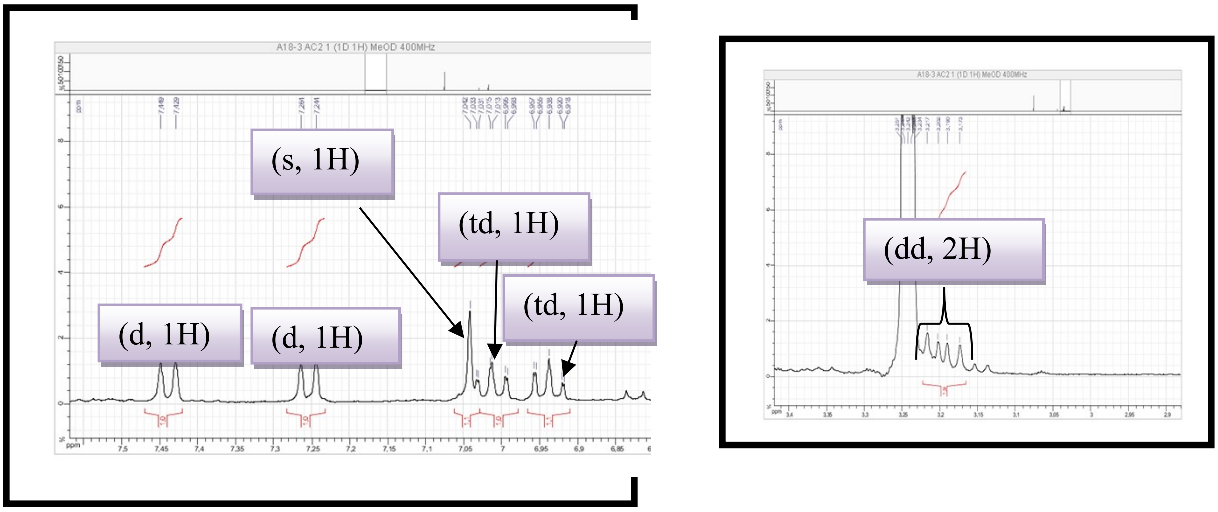

Figure.47-2. ¹H NMR spectrum (spreading out2) (400MHz, CD_3OD, δ_{ppm}) of 3-oxo-α-ionol-β-D-glucopyranoside

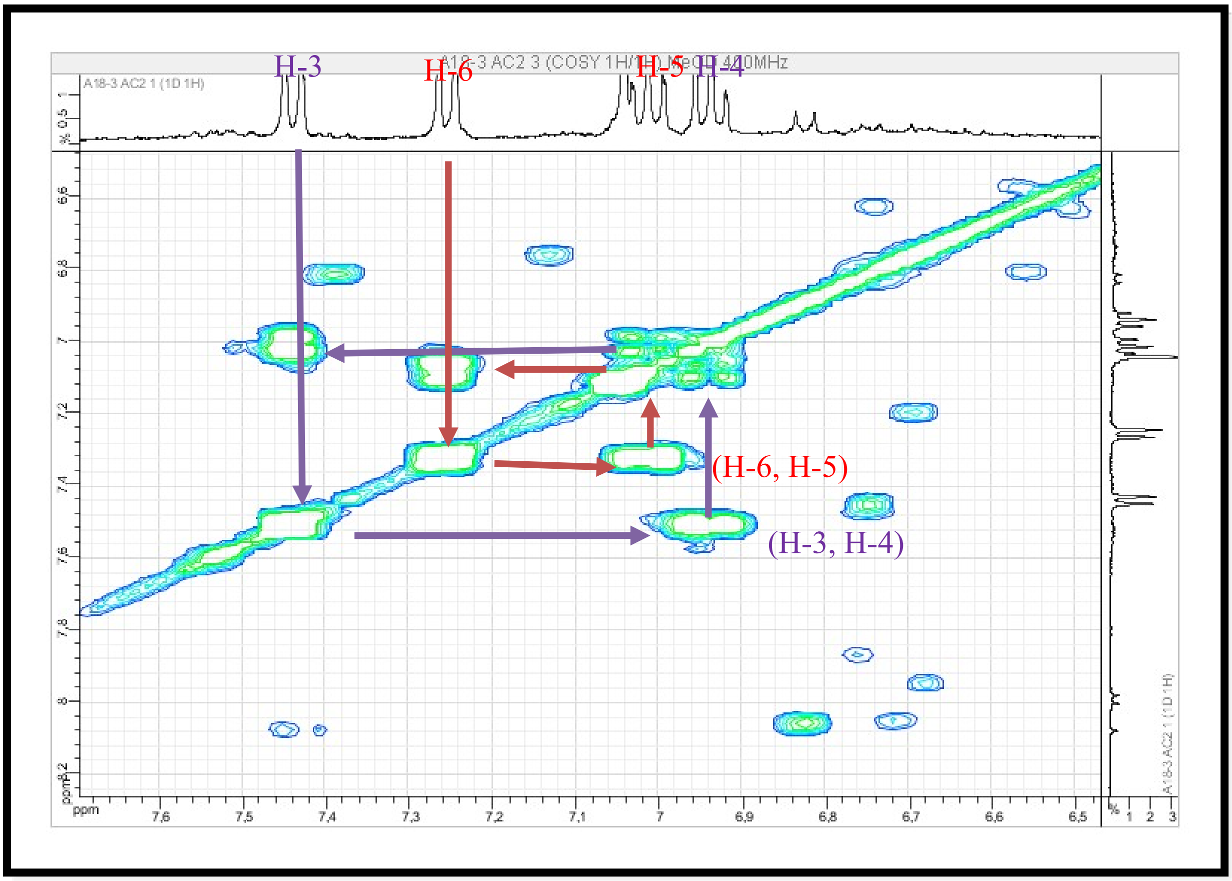

Figure.48-1. COSY spectrum (spreading out 1) (400MHz, CD_3OD, δ_{ppm}) of 3-oxo-α-ionol-β-D-glucopyranoside

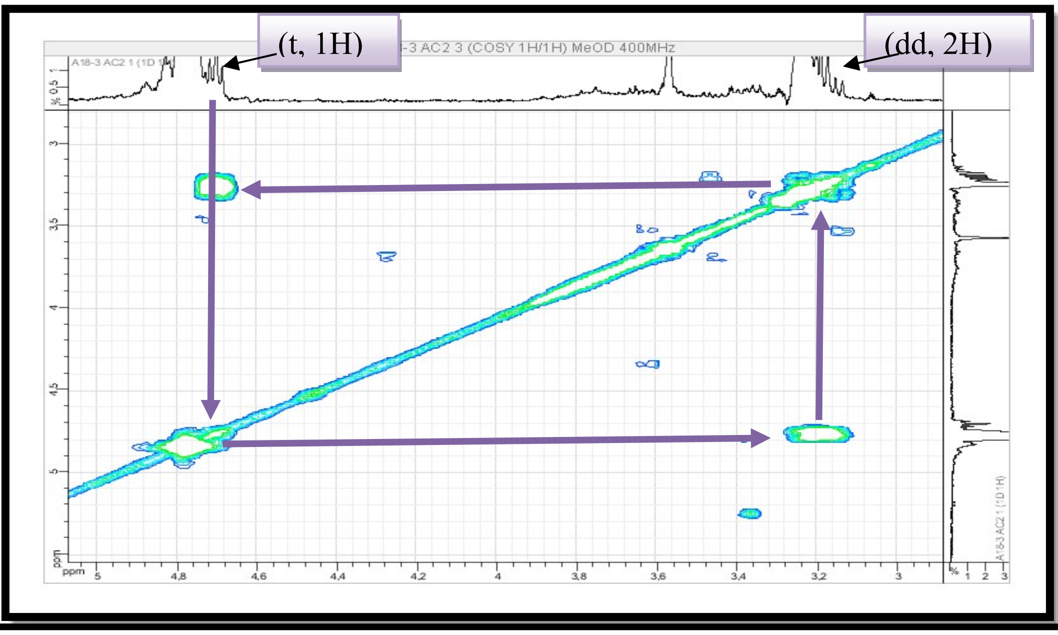

Figure.48-2. COSY spectrum (spreading out 2) (400MHz, CD_3OD, δ_{ppm}) of 3-oxo-α-ionol-β-D-glucopyranoside

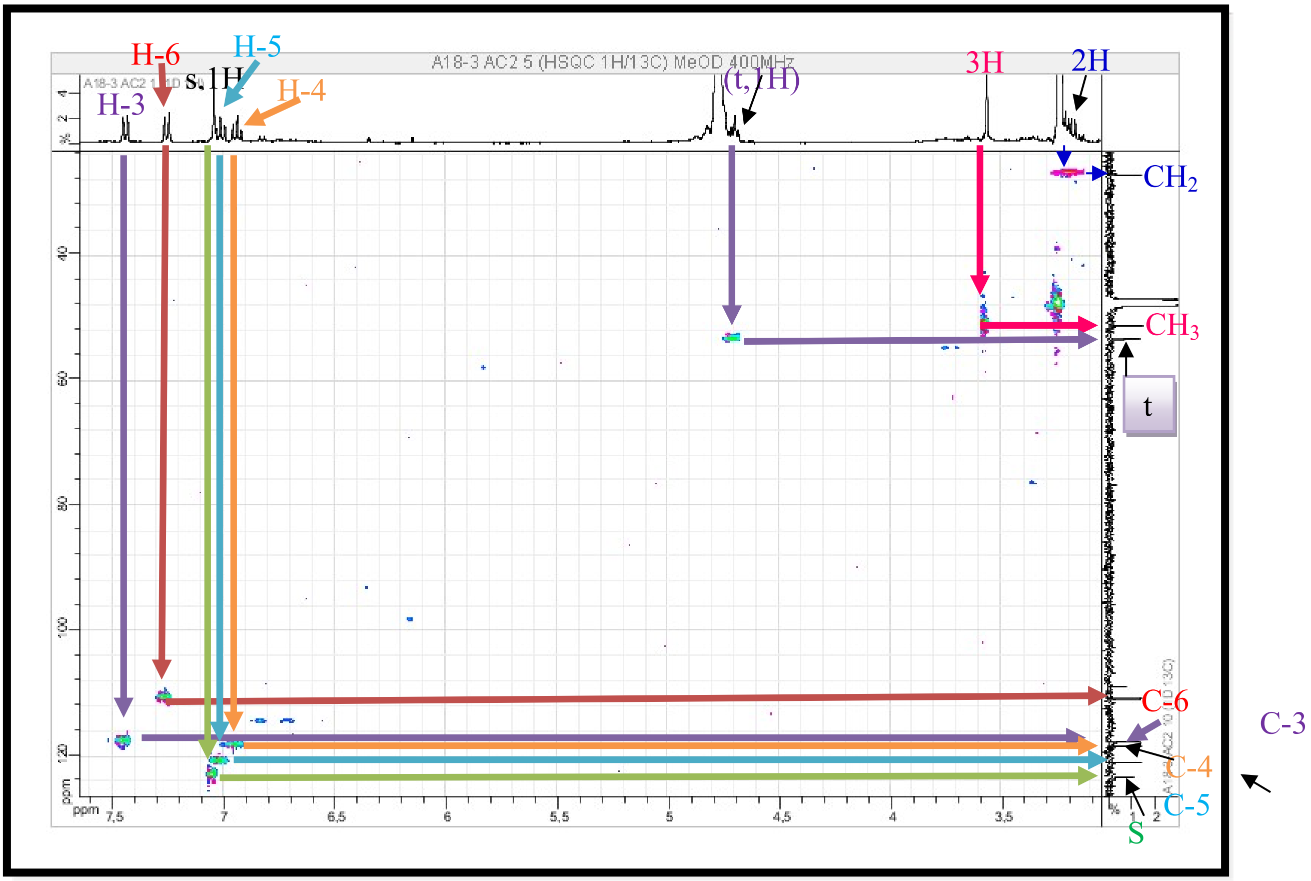

Figure.49. HSQC spectrum (spreading out 1) (400MHz, CD_3OD, δ_{ppm}) of 3-oxo-α-ionol-β-D-glucopyranoside

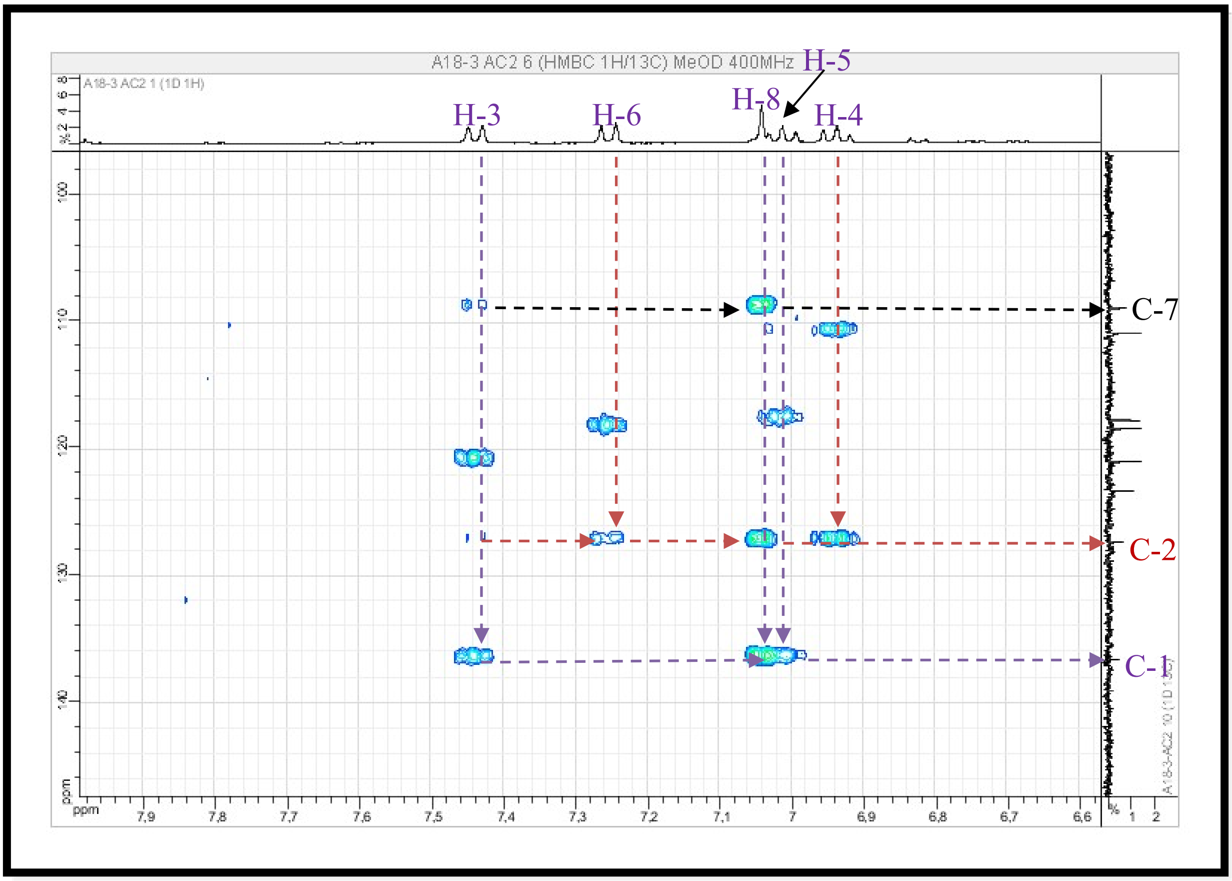

Figure.50-1. HMBC spectrum (spreading out 1) (400MHz, CD_3OD, δ_{ppm}) of 3-oxo-α-ionol-β-D-glucopyranoside

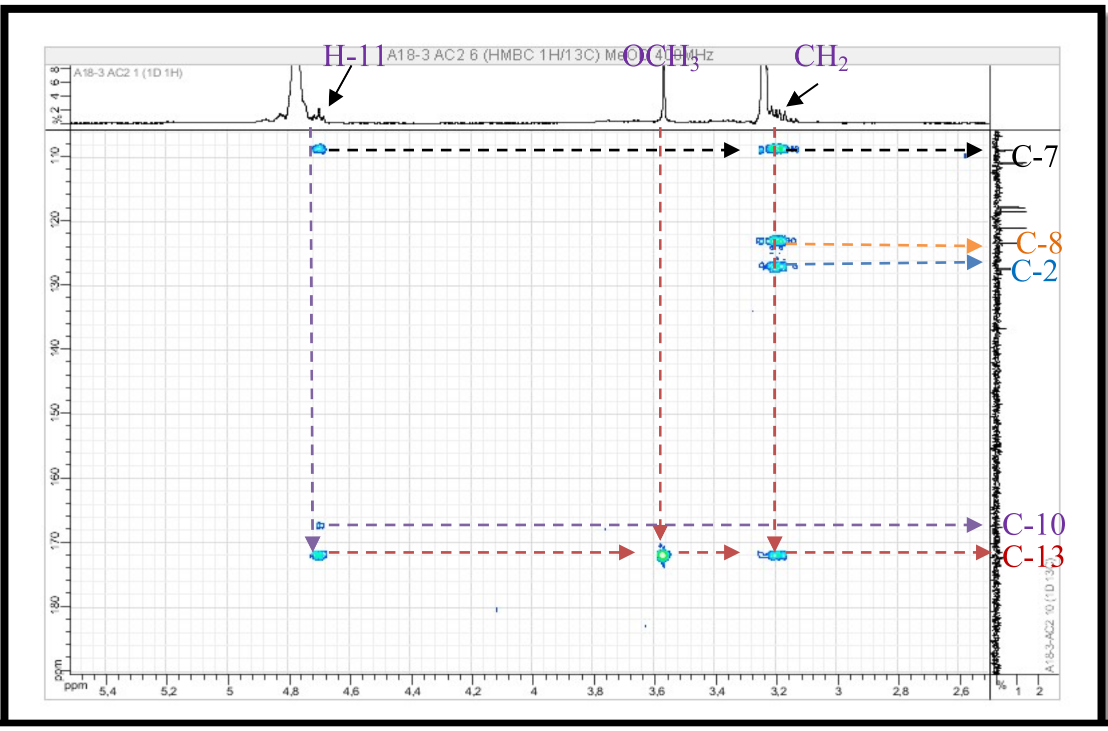

Figure.50-2. HMBC spectrum (spreading out 2) (400MHz, CD_3OD, δ_{ppm}) of 3-oxo-α-ionol-β-D-glucopyranoside

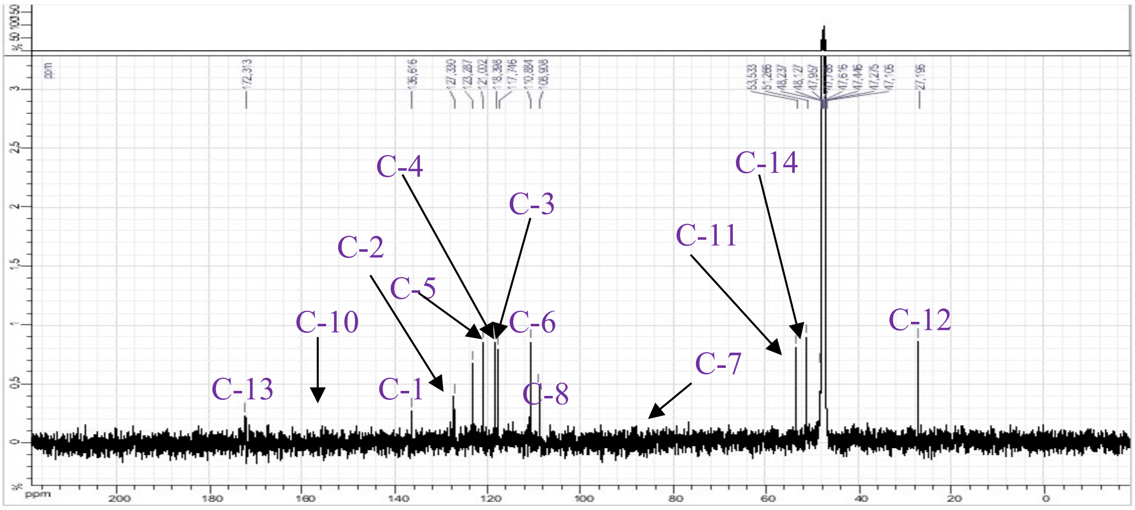

Figure.51. ^{13}C NMR spectrum (100MHz, CD_3OD, δ_{ppm}) of 3-oxo-α-ionol-β-D-glucopyranoside
